# An anti-HER2 biparatopic antibody that induces unique HER2 clustering and complement-dependent cytotoxicity

**DOI:** 10.1038/s41467-023-37029-3

**Published:** 2023-03-13

**Authors:** Nina E. Weisser, Mario Sanches, Eric Escobar-Cabrera, Jason O’Toole, Elizabeth Whalen, Peter W. Y. Chan, Grant Wickman, Libin Abraham, Kate Choi, Bryant Harbourne, Antonios Samiotakis, Andrea Hernández Rojas, Gesa Volkers, Jodi Wong, Claire E. Atkinson, Jason Baardsnes, Liam J. Worrall, Duncan Browman, Emma E. Smith, Priya Baichoo, Chi Wing Cheng, Joy Guedia, Sohyeong Kang, Abhishek Mukhopadhyay, Lisa Newhook, Anders Ohrn, Prajwal Raghunatha, Matteo Zago-Schmitt, Joseph D. Schrag, Joel Smith, Patricia Zwierzchowski, Joshua M. Scurll, Vincent Fung, Sonia Black, Natalie C. J. Strynadka, Michael R. Gold, Leonard G. Presta, Gordon Ng, Surjit Dixit

**Affiliations:** 1 Zymeworks BC Inc., 114 East 4th Avenue, Suite 800, Vancouver, BC Canada; 2 Department of Microbiology and Immunology, University of British Columbia, Vancouver, BC Canada; 3 Department of Biochemistry and Molecular Biology, University of British Columbia, Vancouver, BC Canada; 4 Centre for Blood Research, University of British Columbia, Vancouver, BC Canada; 5 HRMEM Facility, University of British Columbia, Vancouver, BC Canada; 6 Human Health Therapeutics Portfolio, NRC-CNRC, Montreal, QC Canada; 7 Department of Mathematics and Institute of Applied Mathematics, University of British Columbia, Vancouver, BC V6T 1Z2 Canada; 8 AbbVie, 1N. Waukegan Road, North Chicago, IL 60064 USA; 9 Present Address: Department of Urologic Sciences (Vancouver Prostate Centre) and the Institute of Applied Mathematics, University of British Columbia, Vancouver, BC Canada

**Keywords:** Biotechnology, Antibody therapy, Cancer immunotherapy, Cancer therapy, Biologics

## Abstract

Human epidermal growth factor receptor 2 (HER2) is a receptor tyrosine kinase that plays an oncogenic role in breast, gastric and other solid tumors. However, anti-HER2 therapies are only currently approved for the treatment of breast and gastric/gastric esophageal junction cancers and treatment resistance remains a problem. Here, we engineer an anti-HER2 IgG1 bispecific, biparatopic antibody (Ab), zanidatamab, with unique and enhanced functionalities compared to both trastuzumab and the combination of trastuzumab plus pertuzumab (tras + pert). Zanidatamab binds adjacent HER2 molecules in *trans* and initiates distinct HER2 reorganization, as shown by polarized cell surface HER2 caps and large HER2 clusters, not observed with trastuzumab or tras + pert. Moreover, zanidatamab, but not trastuzumab nor tras + pert, elicit potent complement-dependent cytotoxicity (CDC) against high HER2-expressing tumor cells in vitro. Zanidatamab also mediates HER2 internalization and downregulation, inhibition of both cell signaling and tumor growth, antibody-dependent cellular cytotoxicity (ADCC) and phagocytosis (ADCP), and also shows superior in vivo antitumor activity compared to tras + pert in a HER2-expressing xenograft model. Collectively, we show that zanidatamab has multiple and distinct mechanisms of action derived from the structural effects of biparatopic HER2 engagement.

## Abstract (web summary)

The success of HER2-targeted cancer therapy is limited by treatment resistance. Here, the authors engineer an anti-HER2 biparatopic antibody with multiple mechanisms of action including induction of HER2 clustering to trigger complement dependent cytotoxicity, signal inhibition, antibody dependent cellular cytotoxicity and phagocytosis.

## Introduction

HER2 is overexpressed in approximately 20% of breast and gastric/gastroesophageal junction (GEJ) cancers and is a validated therapeutic target for antitumor therapy^1–3^. HER2 overexpression activates a variety of signaling pathways leading to cellular proliferation and tumorigenesis^4^. Unlike other members of the EGFR family, HER2 is capable of constitutive activity, initiating signaling pathways without the need for a bound, activating ligand. HER2 overexpression further leads to HER2 homodimerization and heterodimerization with other members of the EGFR family which can subsequently activate signaling pathways leading to tumor growth. Trastuzumab (Herceptin®) is a classical monoclonal (monospecific) antibody (Ab) that binds to the extracellular domain 4 (ECD4) of HER2 and was the first FDA-approved HER2-targeted therapy for the treatment of HER2-overexpressing breast cancer as well as gastric/ gastric esophageal junction (GEJ) adenocarcinoma^5^. Trastuzumab mediates its antitumor activity via the inhibition of tumor cell proliferation^6^, suppression of cell signaling ^7^ and antibody-dependent cellular cytotoxicity (ADCC)^8^. Pertuzumab (Perjeta®) is another HER2-targeting monospecific Ab that binds to the ECD2 and blocks ligand-induced heterodimer-driven activation^9^. Trastuzumab and pertuzumab have also been evaluated in combination (tras + pert), which has shown enhanced antitumor activity pre-clinically^10^ and clinically^11^ for HER2-positive breast cancer.

Several anti-HER2 agents, including trastuzumab, pertuzumab, lapatinib, neratinib, trastuzumab emtansine (T-DM1), and trastuzumab deruxtecan have been approved for the treatment of HER2-positive breast cancer. Outside of breast cancer, trastuzumab and trastuzumab deruxtecan are approved for the treatment of gastric cancers^3^. However, the clinical benefit of other approved anti-HER2 therapies, such as lapatinib, T-DM1, and pertuzumab, have not universally translated to HER2-positive gastric cancer. In particular, tras + pert plus chemotherapy did not significantly improve OS compared to trastuzumab plus chemo in HER2-expressing gastric/GEJ cancer^12^.

Beyond breast and gastric cancer, HER2 overexpression has been described in other solid tumors, including biliary tract cancer, colorectal cancer, pancreatic cancer, and ovarian cancer, among others^3^, but HER2 therapies have yet to have achieved approval in those cancers. Moreover, intrinsic and acquired resistance to approved HER2 therapies remains a clinical issue as many patients show disease progression over time^13^. Thus, to extend clinical benefit of HER2-targeted therapies to other HER2-expressing solid tumors, and to treat patients with recurrent or metastatic disease that have progressed after standard of care therapy, there is a need for novel anti-HER2 therapies that have greater antitumor activity and differentiated mechanisms of action (MOA)^3^.

In efforts to engineer anti-HER2 therapies with increased antitumor activity, several anti-HER2 bispecific (biparatopic) Abs have been reported to have enhanced antitumor activity compared to trastuzumab or parental Ab combinations^14–18^. Biparatopic Abs are a class of bispecific Ab that target two non-overlapping epitopes on the same target. These anti-HER2 biparatopic Abs show increased receptor internalization and receptor degradation^17,19^, likely promoted by receptor crosslinking^20–22^, which results in enhanced tumor growth inhibition activity. However, none of these biparatopic Abs demonstrated unique cytotoxic antitumor mechanisms that were absent from the parental Ab or parental Ab combinations. As a result, biparatopic Abs to date have been described as a type of ‘combinatorial’ bispecific since they do not provide additional functionalities compared to combinations of parental Abs with the same specificities^23^.

Antitumor Abs often show a variety of MOAs that may operate simultaneously and overlap with each other, such as neutralization of the target antigen and activation of cell-mediated cytotoxicity. Engagement of the complement-dependent cytotoxicity (CDC) pathway has been proposed as a mechanism to increase the therapeutic efficacy of antitumor Abs^24^ and is one of the reported MOA for B-cell targeting monoclonal Abs including rituximab and ofatumumab^25–28^. Trastuzumab, pertuzumab and tras + pert mediate antitumor activity through multiple MOAs but are incapable of eliciting CDC in HER2-expressing cells in the presence of human serum^29–31^. Engagement of the classical complement pathway is governed by a number of factors, including antigen size and density, that impact the ability of the antigen-Ab complex to assume a geometry that allows efficient C1q binding^32^. Optimal CDC activity requires hexameric organization of Ab Fc domains in the Ab-antigen clusters^33^. Among the CDC competent B-cell (CD20) targeting Abs, ofatumumab has been shown to have greater CDC activity compared to rituximab^28,34^, which may be due to the Abs’ distinct membrane proximal epitope and CD20 binding kinetics that enables a geometry that facilitates greater C1q binding and complement activation^27,34^. Strategies that enhance CDC, including Ab hexamerization^33,35^ and Fc mutations^36^ have shown promise for enhancing antitumor activity in preclinical studies. For example, hexamerization was used to generate a B-cell targeting anti-CD37 biparatopic Ab with enhanced in vitro CDC activity^37^.We hypothesized that a biparatopic Ab engineered to enhance receptor crosslinking and clustering may provide the receptor-Ab organization required to bind C1q and engage this potent antitumor mechanism, and maintain and/or enhance all other measurable antitumor MOA ascribed to approved anti-HER2 Ab therapeutics.

We present a comprehensive mechanistic evaluation of zanidatamab (previously referred to as ZW25), a humanized biparatopic IgG1-like Ab directed against ECD4 and ECD2 of HER2. We show that zanidatamab mediates antitumor activity in vitro and in vivo via multiple MOAs in HER2-expressing tumors, including increased tumor cell binding, HER2 internalization, suppression of cell signaling and tumor growth, as well as ADCC, and ADCP. Additionally, we show that zanidatamab elicits CDC in HER2-overexpressing cells, a functionality that is not observed with trastuzumab, pertuzumab or the combination tras + pert. We demonstrate that the geometry of zanidatamab binding and its ability to co-operatively engage adjacent HER2 molecules in *trans*, results in an improved capacity to cluster HER2 and has the ability to induce CDC.

## Results

### Engineering and construction of zanidatamab

**Fig. 1:**
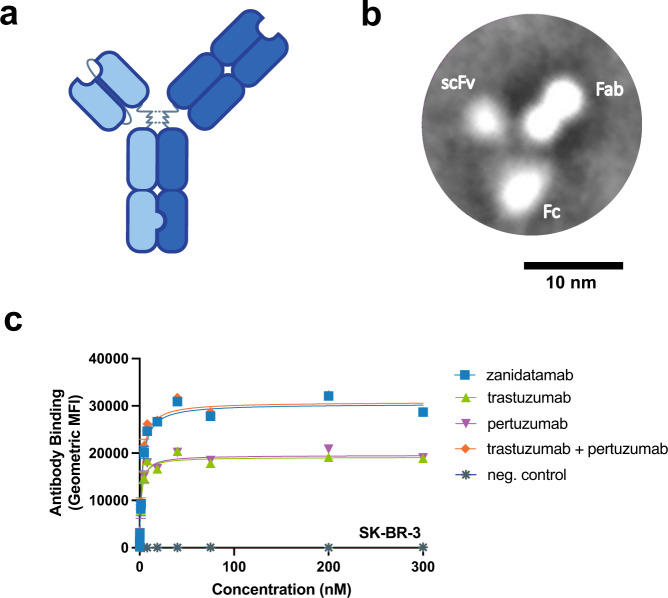
Zanidatamab is a biparatopic anti-HER2 Ab that binds HER2-expressing tumor cells with greater Ab saturation than trastuzumab or pertuzumab. **a** Zanidatamab is a humanized, biparatopic, immunoglobulin 1 (IgG1)-like Ab with an scFv (light blue) that binds the juxtamembrane ECD4 of HER2 and a Fab (dark blue) that binds the ECD2 dimerization domain of HER2, the same domains targeted by trastuzumab and pertuzumab, respectively. **b** Representative class-averaged TEM image, derived from 216 images, showing the 3-lobed structure of zanidatamab with putative assignments of the scFv, Fab, and Fc region from a single experiment. **c** Zanidatamab binds with greater Ab saturation to tumor cell lines compared to trastuzumab or pertuzumab. Flow cytometry was used to quantify the binding of zanidatamab, trastuzumab, pertuzumab, and tras + pert (1:1) to SK-BR-3 tumor cells. In **c**, data are mean ± SEM from *n* = *3* independent experiments. Gating strategy for Ab binding to SK-BR-3 cells (**c**) is shown in Supplementary Fig. 11a. Source data are provided in the Source Data file.

Zanidatamab was constructed using the IgG1-like heterodimeric Azymetric^TM^ Fc platform^38^, with an anti-HER2-ECD4 single chain variable fragment (scFv) linked to heavy chain 1 and an anti-HER2-ECD2 fragment antigen-binding (Fab) domain on heavy chain 2 (Fig. 1a). The scFv and Fab arms of the original precursor biparatopic molecule bound to HER2 ECD with a *K*_D_ of 1 nM and 15 nM, respectively, as determined by surface plasmon resonance (SPR) using monovalent one-armed Ab (OAA) constructs (Supplementary Table 1).

It has been previously reported that Ab combinations and biparatopic Abs can induce target antigen crosslinking, leading to increased receptor-driven internalization and receptor downregulation^20–22^. We hypothesized that our biparatopic Ab, which binds to two non-overlapping epitopes on HER2, would crosslink HER2 and that affinity enhancements to either the anti-HER2-ECD2 Fab or the anti-HER2-ECD4 scFv would further enhance HER2 crosslinking. Computational modeling revealed that increasing the affinity of the weaker anti-ECD2 Fab arm would have the highest impact on the crosslinking ability of the biparatopic Ab (Supplementary Results; Supplementary Fig. 1). Based on these modeling results, we engineered a series of mutations into the lower affinity parental anti-ECD2 Fab using a structure guided approach and screened them as OAA to identify candidates with increased anti-HER2 binding affinity. One variant exhibited similar thermal stability as the parental Ab, as determined by Differential Scanning Calorimetry (DSC) (Supplementary Fig. 1e), and importantly, had an 8.8-fold greater affinity with a *K*_D_ of 1.7 nM for binding the HER2 ECD2 (Supplementary Table 1). This affinity-improved Fab was incorporated into the final anti-HER2 biparatopic Ab, zanidatamab. Zanidatamab bound human HER2 with subnanomolar apparent *K*_D_ of 0.74 nM, as determined by SPR (Supplementary Table 2).

Zanidatamab was imaged by transmission electron microscopy (TEM) negative stain to study its structural characteristics. Zanidatamab is mono-disperse in solution (Supplementary Fig. 1g, h), and TEM imaging revealed that zanidatamab did not oligomerize or aggregate. Two-dimensional averaging of TEM images showed that zanidatamab appears to have the typical Ab “Y” shape with 3 distinct lobes, one appearing like a dumbbell, another as a smaller lobe and the third as a ring, consistent with the Fab-scFv-Fc composition of zanidatamab (Fig. 1a, b, Supplementary Results).

### Zanidatamab binds HER2-expressing tumor cells with increased Ab saturation compared to trastuzumab or pertuzumab

Due to differences in the binding stoichiometry, biparatopic Abs have been shown to bind tumor cells with increased saturation compared to canonical monospecific Abs^15^ (Supplementary Fig. 3a). We assessed cell surface binding and saturation of zanidatamab compared to trastuzumab, pertuzumab and the equimolar tras + pert (1:1 ratio) by flow cytometry to a panel of tumor cell lines which express a range of cell surface HER2 levels, including SK-BR-3 (HER2 3+; Fig. 1c), NCI-N87 (HER2 3+), JIMT-1 (HER2 2+) and MCF7 (HER2 1+) cells (Supplementary Fig. 2). Zanidatamab’s apparent K_d_ values ranged from 2–5 nM and bound with 1.3 to 1.6-fold higher maximum binding capacity (Bmax) compared to trastuzumab or pertuzumab in all cell lines tested. Similar to zanidatamab, tras + pert also bound all HER2-expressing cell lines with higher Bmax compared to trastuzumab or pertuzumab.

### Zanidatamab binds HER2 in *trans* and forms multimeric Ab:HER2 complexes in solution

**Fig. 2:**
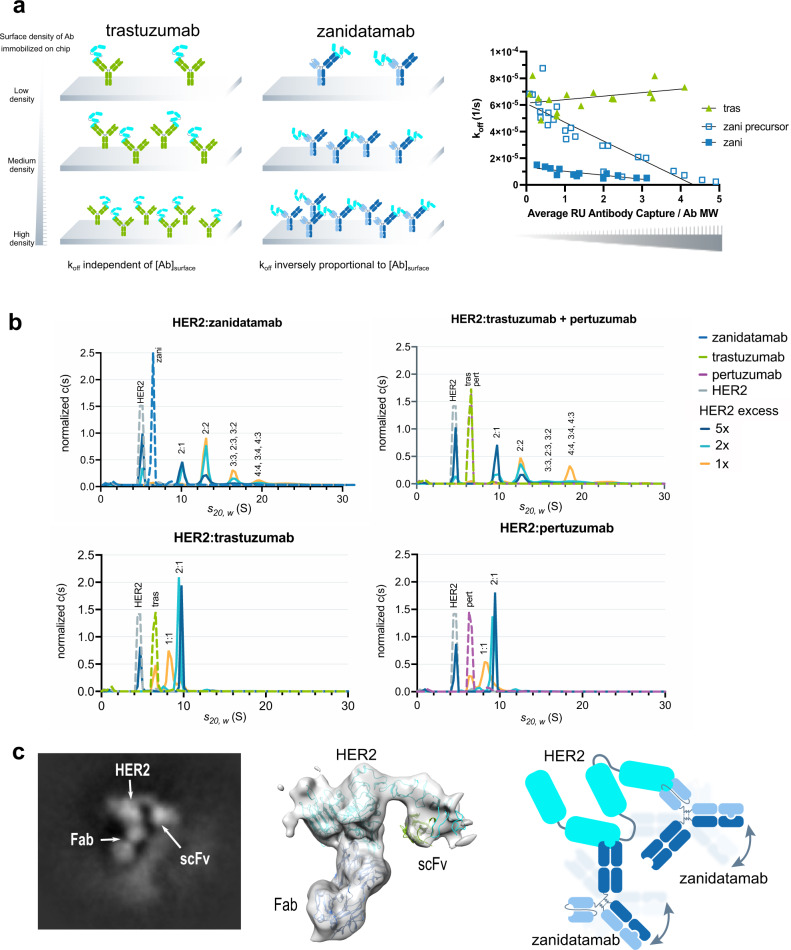
Zanidatamab binds HER2 in *trans* and forms large Ab:HER2 complexes in solution. **a** Cartoon depicts the SPR experimental set up to distinguish between *cis* and *trans* Ab:receptor binding. The dissociation constant *k*_off_ for the monospecific anti-HER2 Ab control (left) is expected to be independent of Ab surface density; the same is expected for a biparatopic Ab binding in *cis*. For a biparatopic Ab binding in *trans*, crosslinking of receptor is expected to increase with increasing Ab chip densities (right), causing *k*_off_ to decrease. Reduction in *k*_off_ observed with increasing zanidatamab and zanidatamab precursor surface concentrations, but not for the control trastuzumab, shows ability of zanidatamab and zanidatamab precursor to bind HER2 in *trans* (right, see Supplementary Table 3). Data from two independent experiments are shown. **b** AUC results for mixtures of HER2 ECD with anti-HER2 Abs at 1-, 2- and 5-fold HER2 excess. For reference, HER2 ECD and Abs were run separately and plotted with the HER2:Ab mixtures. For zanidatamab and tras + pert, higher order complexes were detected with increasing amounts when the excess of HER2 ECD was reduced. Tras or pert formed mainly 1:1 and 2:1 HER2:Ab complexes and only trace amounts of higher order complexes were detected. Complex compositions larger than 2:1 (HER2:Ab) are approximate. **c** Representative cryo-EM 2D class average, 3D reconstruction at 7.6 Å; resolution (middle) showing a core HER2 molecule bound to zanidatamab Fab and scFv, and a cartoon representation of the zanidatamab:HER2 complex observed by cryo-EM (right). The fuzzy halo observed in the 2D images is likely due to the Fc domain in multiple conformations. A model of HER2 (cyan) in complex with zanidatamab Fab (green) and scFv (blue) is also shown. The distance between the C-termini of the two antigen-binding domains cannot be spanned by a single zanidatamab molecule (see Supplementary Fig. 4). Because this structural constraint prevents zanidatamab from binding to HER2 in *cis*, a single zanidatamab molecule can only bind in *trans* and crosslink two HER2 molecules by binding to the ECD2 on one and the ECD4 on another. Source data are provided in the Source Data file.

To understand the mechanism of our biparatopic Ab binding to HER2 ECD and gain information on the ability for *cis* and *trans* receptor binding (binding a single HER2 molecule *vs*. binding two distinct molecules, Supplementary Fig. 3a), we used SPR to assess the stoichiometry and kinetics of HER2 ECD binding. We compared the HER2 binding kinetics of zanidatamab, our anti-HER2 biparatopic precursor molecule (with the 8-fold lower affinity anti-ECD2 binding arm; zanidatamab precursor) and trastuzumab over increasing densities of immobilized Ab. The dissociation rate (*k*_off_) of HER2 ECD from trastuzumab would be independent of Ab density because of its obligate 1:1 binding mode; HER2 ECD is bound by trastuzumab only at ECD4 regardless of Ab concentration (Fig. 2a cartoon, left). If the biparatopic zanidatamab binds to HER2 ECD in *cis*, its dissociation rate *k*_off_ would also be expected to be independent of Ab density, as for trastuzumab, because of its 1:1 binding mode. If the biparatopic Ab binds to HER2 ECD in *trans*, its dissociation rate *k*_off_ would be expected to decrease with increasing Ab density on the chip surface because HER2 ECD can be crosslinked by two molecules of zanidatamab through ECD2 and ECD4 at high Ab density, but not at very low Ab density (Fig. 2a cartoon). SPR assay results showed the dissociation rate *k*_off_ decreased with increasing density of captured zanidatamab and zanidatamab precursor, whereas the dissociation rate *k*_off_ for trastuzumab remained constant at all surface densities tested (Fig. 2a, right; Supplementary Table 3). Additionally, a reduction in apparent *K*_D_ (increased binding affinity) was observed as a consequence of k_off_ decrease over increasing Ab capture concentrations with the zanidatamab and zanidatamab precursor but not with trastuzumab (Supplementary Fig. 3b, Supplementary Table 3). These observations support the hypothesis that biparatopic antibodies such as zanidatamab can bind HER2 ECD in *trans* and that the frequency of *trans*-binding is increased at higher Ab concentrations.

To determine the nature of complexes formed by zanidatamab and HER2 ECD in solution, analytical ultracentrifugation (AUC) with different mixed molar ratios of Ab and HER2 was employed. Zanidatamab (125 kDa), trastuzumab (145 kDa), pertuzumab (145 kDa) or tras + pert (at 1:1 ratio) were mixed in solution phase with HER2 ECD (72 kDa) at 1-, 2- and 5-fold molar excess and each component and mixtures were characterized by AUC in a sedimentation velocity experiment. The sedimentation coefficient S_20, w_ of peaks in the AUC experiment correlates with molecular weight, while the area under the peaks in the normalized sedimentation coefficient distribution (c(s)) correlates with the relative amount of the species detected (Fig. 2b). Zanidatamab formed 2:1 (HER2:Ab) and higher order multimeric complexes with HER2 ECD and only trace amounts of 1:1 complex. Multimeric complex formation for zanidatamab was observed even when HER2 ECD was in molar excess. With HER2 ECD in 5-fold molar excess, zanidatamab predominantly formed 2:1 complexes and a small amount of 2:2 complexes. When the molar excess of HER2 ECD relative to zanidatamab was decreased from 5-fold to 1-fold, the relative quantity of multimeric complexes increased to approximately 95% of the total peak area, presenting 2:2, 3:3 (or 2:3, 3:2) and 4:4 (or 4:3, 3:4) complexes. Complexes larger than 4:4 were detected at approximately 10% of the total peak area at equimolar concentration of HER2 ECD and zanidatamab. HER2 ECD mixed with tras + pert behaved similarly with increasing higher order multimeric complexes when the molar excess of HER2 was decreased, with some differences in the peak distribution. Trastuzumab or pertuzumab formed mainly 2:1 complexes when HER2 ECD was in excess, and only small quantities (<3% of the total peak area) of larger assemblies. At equimolar concentrations of HER2 with trastuzumab or pertuzumab, 1:1 complexes predominated, which is not the case for zanidatamab or tras + pert. Evidence for the formation of larger complexes in solution driven by anti-HER2 biparatopic molecules has been presented before^17,19^. Oganesyan et al.^39^ similarly employed AUC to demonstrate the formation of large biparatopic-driven HER2 complexes, which were not observed with the parental Ab.

We further examined zanidatamab in complex with HER2 ECD by cryo-EM. 2D class averaging revealed a core HER2 molecule bound to zanidatamab Fab and scFv (Fig. 2c and Supplementary Fig. 4a). The diffuse background observed on the periphery of the images is likely formed by the multiple conformations of Fc and the second antigen-binding domain of the two Abs bound to the resolved HER2 ECD (Fig. 2c, cartoon). Cryo-EM 3D reconstruction at 7.6 Å; resolution was used to fit the model of HER2 complexed with zanidatamab’s Fab and scFv (Fig. 2c). Our model reveals that the orientation and distance between the Fab and scFv domains cannot be bridged by a single zanidatamab hinge (Supplementary Fig. 4b) and must belong to two distinct zanidatamab molecules. Since HER2 ECD adopts a rigid conformation^40^ these structural constraints preclude zanidatamab from binding to HER2 in *cis*, as previously illustrated by Oh and Bang^3^. This further demonstrates that zanidatamab must bind HER2 in *trans* and crosslink two HER2 molecules by binding to the ECD2 on one and the ECD4 on another. Similar conclusions for the inability of a biparatopic Ab to bind in *cis* based on a crystal structure model have been previously presented by Oganesyan et al.^39^.

Collectively, these results support the concept that zanidatamab binds HER2 ECD in *trans* leading to zanidatamab:HER2 multimerization.

### Zanidatamab binding induces the formation of HER2 caps on the cell surface

**Fig. 3:**
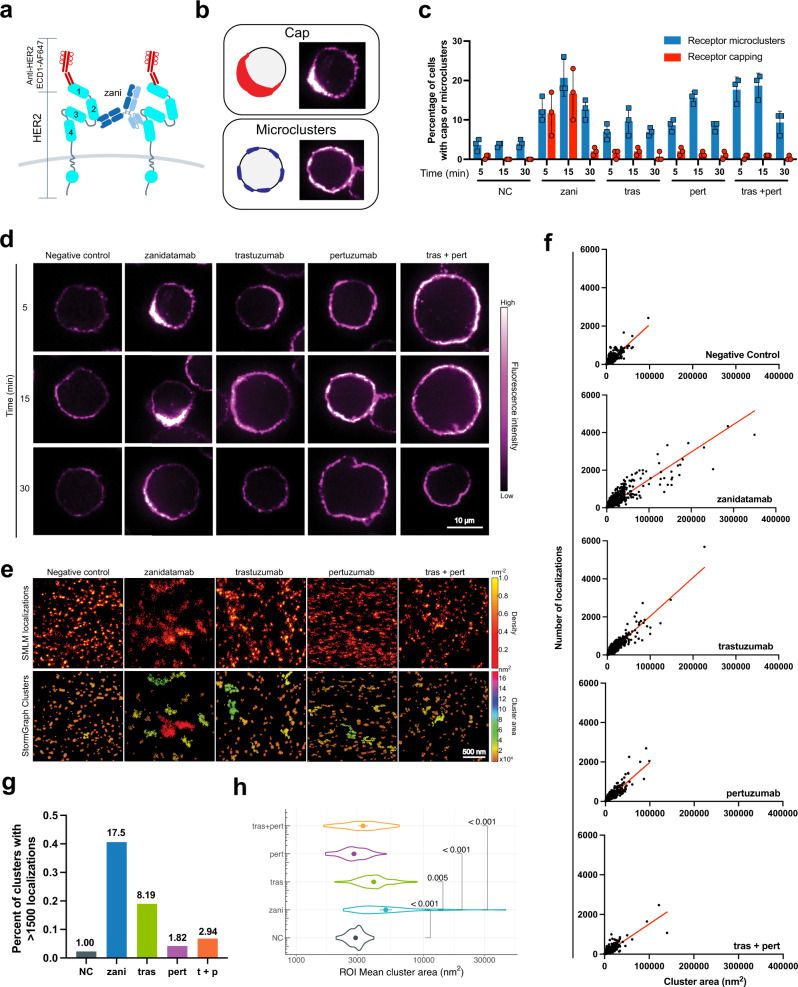
Zanidatamab binding induces HER2 capping and the formation of large HER2 clusters on the cell surface. **a** Illustration showing zanidatamab bound in *trans* to ECD2 and ECD4 of two HER2 molecules along with the anti-HER2 ECD1-AF647 used to detect HER2 localization following Ab treatment. HER2 extracellular domains ECD1-4 and tyrosine kinase (TK) domains are labeled. **b** Graphical representation of cell surface HER2 caps vis-a-vis microclusters and representative 3D reconstructed confocal microscopy images. **c**, **d** SK-BR-3 cells were treated with 200 nM anti-HER2 or negative control (NC) Ab palivizumab for the indicated times. Cells where then imaged by confocal microscopy following HER2 detection with 74 nM anti-HER2 ECD1-AF647 OAA (fluor to Ab ratio of 8.9). Percent of cells exhibiting microclusters or caps is shown as the mean ± SEM for three independent experiments (**c**). Representative confocal images are shown from 30-43 images (185-351 cells) per Ab treatment condition from three independent experiments, scale bar = 10 μm (**d**). (**e**) SK-BR-3 cells were treated with 200 nM anti-HER2 Abs or control Ab for 15 min and imaged by dSTORM super-resolution microscopy. Upper row shows HER2 dSTORM localizations (detected with 148 nM anti-HER2 ECD1-AF647 OAA) in representative ROIs. Color bars = localization density. Lower row shows HER2 clusters identified by the StormGraph clustering algorithm and color-coded by cluster areas (nm^2^). Scale bar = 500 nm. For each Ab treatment condition, 55-124 images (ROI) from multiple cells were imaged. **f** The number of localizations per cluster is graphed versus cluster area and the best-fit linear regression is shown. **g** Percent of clusters with > 1500 localizations. Numbers above the bars are fold-difference versus NC. **h** The mean cluster area was determined for each ROI. The violin plot shows the distributions of mean cluster areas for each Ab treatment. Pairwise comparisons were performed using a two-sided non-paired *t*-test on a log_10_ scale and *p* values were corrected using the Bonferroni method. Black brackets indicate comparison and *p* value (zani *vs*. tras df = 133, *p* = 0.005; zani *vs*. pert df = 135, zani *vs*. tras + pert (1:1) df = 147, and zani *vs*. NC df = 116, *p* < 0.001). Source data are provided in the Source Data file.

To determine whether HER2 crosslinking induced by the *trans* binding of zanidatamab translates into distinct clustering geometries of HER2 on the surface of tumor cells, we used confocal microscopy to image Ab-induced HER2 spatial organization (detected using the monovalent anti-HER2 ECD1-AF647 OAA) in high HER2-expressing SK-BR-3 cells (Fig. 3). We used 3D spinning disk confocal microscopy to visualize the microscale organization of HER2 on randomly selected cells and to determine whether the different anti-HER2 Abs caused observable differences in HER2 organization or clustering. SK-BR-3 cells treated with the negative control Ab palivizumab (anti-RSV protein F), which does not bind HER2, exhibited a relatively uniform cell surface distribution of HER2 with occasional HER2 patches or microclusters (Fig. 3b, c, d). In contrast, 3D reconstructions of cells that had been treated with zanidatamab, trastuzumab, pertuzumab, or tras + pert revealed Ab-induced reorganization and clustering of HER2 on the cell surface, with a significantly greater fraction of cells exhibiting distinct microclusters (Fig. 3b–d, Supplementary Movie 1, Supplementary Fig. 6). Within 5 min of addition to cells, all anti-HER2 Abs induced HER2 microcluster formation (Fig. 3c, d), although to varying degrees. The percentage of cells with microclusters peaked at 15 min for all Ab treatments and declined by 30 min after Ab addition.

Although all anti-HER2 Abs tested induced the formation of microclusters, approximately 10-15% of the zanidatamab-treated SK-BR-3 cells displayed large, polarized aggregates of HER2 on one side of the cell, which we refer to as a HER2 ‘cap’ (Fig. 3b–d, Supplementary Fig. 6 and Supplementary Movie 1). These HER2 caps were readily distinguishable from smaller microclusters in both 3D reconstructed images (Supplementary Movie 1) and in 2D confocal slices showing the plane of the cell with the greatest HER2 fluorescence (Fig. 3c, d). The percent of zanidatamab-treated cells with HER2 caps increased from 5 min to 15 min but the HER2 caps were largely absent after 30 min., although fluorescence polarized to one side of the cell was still observed. The HER2 caps were not observed with trastuzumab, pertuzumab, or the tras + pert combinations (Fig. 3c, d) at any time point tested. Pairwise comparisons using a binomial logistic regression model showed that only zanidatamab binding induced significant cap formation at the 5 and 15 min (*p* < 0.001) time points in SK-BR-3 cells (Supplementary Fig. 6c). The ability of zanidatamab to induce HER2 cap formation was confirmed in an additional HER2-expressing cell line (NCI-N87), with the level of HER2 caps formation facilitated by zanidatamab greater than that observed with trastuzumab or pertuzumab alone. Tras + pert also induced HER2 cap formation in NCI-N87 cells but to a lesser extent than zanidatamab (Supplementary Fig 6d–f). In summary, these data show that zanidatamab binding significantly induces distinct cell surface HER2 clustering, observed as a polarized ‘HER2 cap’, that is not observed with trastuzumab or pertuzumab.

### Zanidatamab binding induces large cell surface HER2 clusters

Having observed that zanidatamab binding induced significantly different microscale organization of HER2 on the cell surface, we hypothesized that zanidatamab would also induce distinct changes to HER2 clustering at the nanoscale level. To test this, we performed single molecule localization microscopy (SMLM) and used stochastic optical reconstruction microscopy (dSTORM) to determine the localization coordinates of HER2 molecules on the surface of Ab-treated SK-BR-3 cells. Quantitative analysis of HER2 clustering was performed using StormGraph^41^, a graph theory-based clustering algorithm.

SMLM and StormGraph analysis revealed that zanidatamab altered the local nanoscale clustering of HER2 to a greater extent than trastuzumab, pertuzumab, or tras + pert (Fig. 3e). Detection of HER2 on SK-BR-3 cells (with the anti-ECD1-AF647 Ab) following treatment with the negative control Ab, which does not bind HER2, showed many small HER2 clusters. In contrast, HER2 clusters on zanidatamab-treated cells were larger, with a corresponding decrease in the number of small HER2 clusters (Fig. 3e). Importantly, zanidatamab treatment induced the formation of larger clusters, as well as a greater number of large clusters, than trastuzumab, pertuzumab, or tras + pert.

To quantify these differences in HER2 clusters sizes, for each Ab treatment we generated graphs of cluster area versus the number of HER2 localizations per cluster (Fig. 3f). In dSTORM, fluorescent molecules undergo multiple cycles of excitation and emission (i.e. blinking) such that a single fluorophore molecule yields multiple localizations^42,43^. Moreover, each molecule of the anti-HER2 ECD1-AF647 OAA detection reagent that we used was labeled with an average of 8-9 AF647 molecules. Hence, although they are related, it is difficult to determine the extent of ‘overcounting’, i.e., the number of dSTORM localizations per individual molecule. Mathematical approaches have been used to estimate the local degree of overcounting (described in^44,45^) but they often rely on multiple assumptions. Therefore, we report the experimentally determined number of HER2 localizations per cluster.

For each Ab treatment, we plotted the number of localizations per cluster versus cluster area for each cluster (>2900 clusters per condition) (Fig. 3f). Note that there was a largely linear relationship between cluster area and the number of localizations per cluster for all Ab treatments (*R*^2^ = 0.765–0.855) (Fig. 3f). For all Ab treatments, 97–99% of the HER2 clusters detected (>2900 clusters per condition) contained fewer than 500 localizations (Fig. 3f). Importantly, consistent with the dSTORM images in Fig. 3e, SK-BR-3 cells treated with zanidatamab, and to a lesser extent trastuzumab, had a greater number of large clusters (Fig. 3f). Relative to the control Ab, zanidatamab treatment increased the percent of clusters with >1500 localizations by 17.5-fold whereas trastuzumab caused an 8.2-fold increase while pertuzumab and tras + pert caused much smaller increases in the frequency of these large clusters (Fig. 3g).

To analyze the local effects of the different Ab treatments on HER2 clustering, we calculated the mean cluster area for all of the clusters within each 2 µm × 2 µm region of interest (ROI) that we imaged for each Ab treatment (55-124 ROIs from multiple cells) and then compared the distribution of these values (Fig. 3h). On SK-BR-3 cells treated with the negative control Ab, HER2 localizations were present mainly in small clusters (Fig. 3e, f) and the ROI mean cluster areas ranged from 2055–4061 nm^2^ (geometric mean: 2921 nm^2^) (Fig. 3h). The mean cluster areas for the ROIs on pertuzumab-treated cells ranged from 1699–5075 nm^2^ with a geometric mean of 2835 nm^2^, similar to that of control Ab-treated cells. Trastuzumab treatment increased this geometric mean value to 4052 nm^2^, with the 2009–8846 nm^2^ range of cluster areas reflecting a modest increase in the frequency of ROIs with larger mean cluster areas. Interestingly, the ROI mean cluster areas (1641–6425 nm^2^ range) observed after tras + pert treatment had a geometric mean of 3343 nm^2^, which was intermediate in size between the geometric mean values for either trastuzumab or pertuzumab alone. Zanidatamab treatment caused the largest increase in ROI mean cluster area with a resulting geometric mean of 4919 nm^2^ and a range of 2343–43,915 nm^2^. Pairwise comparisons of the means confirmed that zanidatamab significantly increased the ROI mean HER2 cluster area compared to trastuzumab (*p* = 0.005), pertuzumab or tras + pert (*p* < 0.001) (Fig. 3h). Moreover, only zanidatamab treatment resulted in ROIs with mean cluster areas >10,000 nm^2^. These data indicate that zanidatamab induced the formation of large HER2 clusters in many ROIs and did so to a significantly greater extent than trastuzumab, pertuzumab, or tras + pert.

### Zanidatamab promotes potent CDC and increases deposition of C1q binding and C3 fragments on HER2-expressing tumor cells

**Fig. 4:**
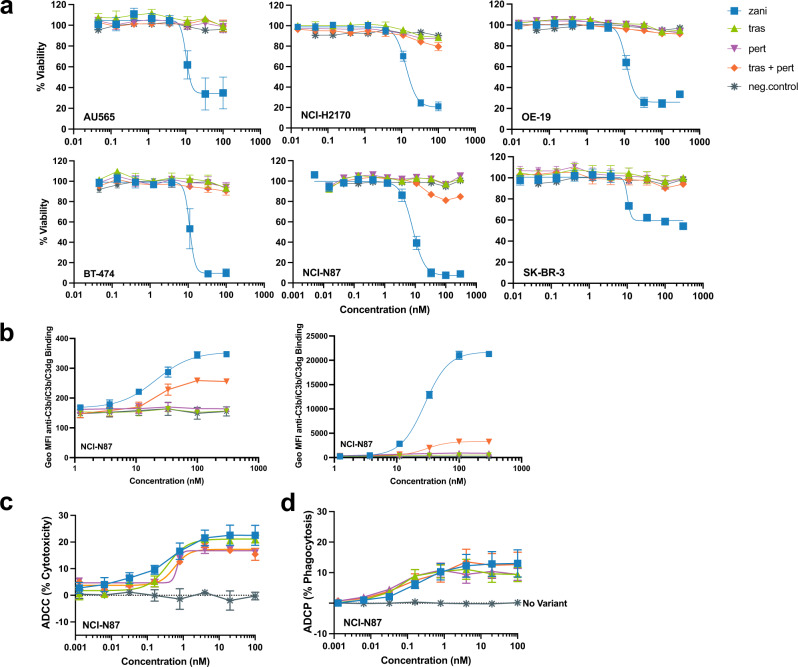
Zanidatamab mediates Fc effector functions including potent CDC, and ADCC and ADCP. **a** Zanidatamab mediated CDC with normal human serum (NHS) in high HER2-expressing tumor cells (AU565, NCI-H2170, OE-19, NCI-N87, BT-474, SK-BR-3; HER2 3+)**;** trastuzumab, pertuzumab, and tras + pert (1:1) are inactive. **b** Zanidatamab mediated the highest C1q and C3 fragment (C3b/iC3b/C3dg) deposition on NCI-N87 cells in the presence of NHS. Zanidatamab and tras + pert binding in presence of NHS resulted in enhanced C1q (left) C3b/iC3b/C3dg (right) deposition in NCI-N87 cells compared to trastuzumab, pertuzumab or negative control. Zanidatamab mediated concentration-dependent ADCC (**c**) and ADCP (**d**) in NCI-N87 cells with comparable activity to trastuzumab, pertuzumab and tras + pert. In **a**–**d**, data are mean ± SEM from *n* = 3 (AU565, NCI-H2170, OE-19, BT-474, SK-BR-3) or *n* = 6 (NCI-N87) independent experiments. In **b**–**d** data are mean ± SEM from *n* = 3 independent experiments. In **c**, **d** *n* = 3 independent experiments performed with biologically independent PBMC samples (**c**), or with macrophage derived from three biologically independent PBMC samples (**d**). Gating strategy for **b**, **c** and **d** is shown in Supplementary Fig. 12d, a, b, respectively. Source data are provided as a Source Data file.

Next, we asked whether the increased saturation and HER2 clustering observed with zanidatamab would translate into higher Fc-effector function activity, such as CDC, compared to trastuzumab, pertuzumab, or tras + pert. We selected a panel of high HER2-expressing (HER2 3+) cell lines (Table 1) derived from breast (BT-474, SK-BR-3, ZR-75-30, AU565, HCC1419), gastric (NCI-N87), esophageal (OE-19), and lung (NCI-H2170) tumors, and evaluated CDC by measuring cell viability following Ab treatment in the presence of normal human serum (NHS) as a complement source. For all high HER2-expressing cell lines, zanidatamab-mediated concentration-dependent CDC with mean IC_50_ values ranging from 7 to 13 nM and induced approximately 40 to 95% cell death (i.e. decreased the percent of viable cells ranging from 4 to 60%) (Fig. 4a, Table 1). Tras + pert mediated weak CDC in NCI-N87 cells at the highest antibody concentrations tested, and to a much lower extent than zanidatamab, but did not induce CDC-mediated cell death in any other HER2-expressing cancer cell lines (Table 1, Fig. 4a, Supplementary Fig. 7f, g). To verify that the antitumor activity observed was CDC-dependent, the complement cascade components in NHS were heat inactivated or tested in the presence of EDTA (NHS + 0.01 M EDTA). CDC was not observed with the heat-inactivated NHS, or with NHS + 0.05 M EDTA for any of the Abs tested (Supplementary Fig. 7a, h, i). We then evaluated CDC activity in four low-HER2-expressing cancer cell lines including JIMT-1, ZR-75-1, MCF7, and MDA-MB-175-VII and did not observe CDC with any anti-HER2 Abs including zanidatamab (Supplementary Table 4). CDC was not observed for trastuzumab or pertuzumab in any of the cell lines tested (Table 1, Supplementary Table 4). We further evaluated CDC activity by adding NHS to the tumor cells (5 min, 37°C), followed by antibody treatment and observed similar activity where only zanidatamab elicited CDC in all cell lines tested (Supplementary Results, Supplementary Fig. 7f, g). Thus, zanidatamab uniquely supported substantial CDC in multiple high HER2-expressing cancer cell lines but not in the limited number of low-HER2-expressing cancer cell lines that were evaluated.

**Table 1:** Percent complement-dependent cytotoxicity at maximal effect values of anti-HER2 Abs in HER2 expressing cancer cell lines

Cell line	Cancer type	HER2 IHC overall score^a^	Average HER2 receptors/cell^b^	Percent of viable cells remaining following treatment at maximal effect (95% CI)^c^				
				Zanidatamab	Trastuzumab	Pertuzumab	Tras + pert	Negative control
HCC1954	Breast	3+	7.03 × 10^6^	49.8 (46, 54)	Inactive	Inactive	Inactive	Inactive
HCC1419	Breast	3+	6.58 × 10^6^	6.18 (2.3, 10)	Inactive	Inactive	Inactive	Inactive
ZR-75-30	Breast	3+	5.36 × 10^6^	4.07 (1.7, 6.4)	Inactive	Inactive	Inactive	Inactive
AU565	Breast	3+	4.54 × 10^6^	34.3 (28, 40)	Inactive	Inactive	Inactive	Inactive
NCI-H2170	Lung	3+	4.40 × 10^6^	20.6 (16, 26)	Inactive	Inactive	Inactive	Inactive
OE-19	Esophageal	3+	3.44 × 10^6^	24.6 (21, 28)	Inactive	Inactive	Inactive	Inactive
BT-474	Breast	3+	3.43 × 10^6^	9.45 (4.2, 15)	Inactive	Inactive	Inactive	Inactive
SK-BR-3	Breast	3+	3.40 × 10^6^	59.7 (56, 64)	Inactive	Inactive	Inactive	Inactive
NCI-N87	Gastric	3+	2.67 × 10^6^	10.7 (6.4, 15)	Inactive	Inactive	76.6 (48, 110)	Inactive

*95% CI* 95% confidence interval.

^a^HER2 expression by IHC was determined with HercepTest (Dako, Carpinteria, California). Microscopic scoring of tumor cells was interpreted as described in the package insert.

^b^ Values are mean from 3 to 6 separate experiments. Values extrapolated from standard curve generated with four bead standards, each with a different Ab binding capacity (ABC), the highest was 5.34 × 10^5^ ABC/bead.

^c^ Values are mean and 95% CI of 3–7 separate experiments. Percent viability (% viability) of treated cells at maximal effect determined by Best-fit Bottom value derived from the 4-parameter log(inhibitor) vs. response sigmoidal curve model fits using GraphPad Prism 9.2.0 (GraphPad Software, Inc., La Jolla, CA).

To elucidate the basis for the enhanced ability of zanidatamab to induce CDC, we analyzed early steps of complement engagement and classical pathway activation. We used flow cytometry to assess the deposition of C1q, the first component of the complement cascade, and C3 fragments (C3b/iC3b/C3dg), subsequent cascade components upstream of the membrane attack complex formation, on a representative HER2 3+ cell line, NCI-N87, following Ab binding in the presence of NHS. Zanidatamab mediated the highest deposition of C1q and C3 fragments of all anti-HER2 Ab treatments examined (Fig. 4b). Zanidatamab and tras + pert mediated concentration-dependent C1q deposition, whereas no C1q deposition was detected with trastuzumab or pertuzumab. Zanidatamab and tras + pert mediated concentration-dependent C3 fragment deposition, whereas trastuzumab and pertuzumab showed weak C3 fragment deposition on NCI-N87 cells. Compared to tras + pert, zanidatamab demonstrated 1.4 and 6.5-fold higher maximum geometric MFI of C1q and C3 fragments including C3b/iC3b and C3dg, respectively. We further compared C3b/iC3b/C3dg and C3b/iC3b deposition on SK-BR-3 cells in the presence of NHS. As observed in NCI-N87 cells, zanidatamab mediated the highest C3b/iC3b/C3dg or C3b/iC3b deposition compared to tras + pert, whereas trastuzumab or pertuzumab mediated no C3 fragment deposition. (Supplementary Results, Supplementary Fig. 8a).

We also evaluated the CDC activity of zanidatamab and tras + pert in the presence of rabbit complement serum to compare to the published CDC data with tras + pert in high HER2-expressing (HER2 3+) tumor cells^46^. Zanidatamab and tras + pert showed CDC activity in NCI-N87, BT-474 and OE-19 cells with rabbit complement serum. Zanidatamab showed an approximate 3 to 4-fold lower IC_50_ compared to tras + pert, while trastuzumab or pertuzumab were inactive (Supplementary Fig. 7b).

We also evaluated the effects of varying pre-opsonization (zanidatamab incubated with tumor cells for 5, 15, 30 min, and 1 h prior to serum addition) and NHS incubation time points (5, 15, 30 min, and 1 h), as well as the effects of varying the percent of NHS (0-80% NHS), on CDC activity with zanidatamab in both SK-BR-3 and NCI-N87 cells. Under all conditions tested with NHS, zanidatamab elicited CDC activity (Supplementary Results, Supplementary Fig. 7c–e). Together, these data establish CDC as a rapid and consistently differentiating cytotoxic mechanism of action of zanidatamab, which is not observed with trastuzumab, pertuzumab, or tras + pert.

### Zanidatamab mediates ADCC and ADCP

To evaluate additional Fc-mediated effector functions, we evaluated the ability of zanidatamab to mediate ADCC and ADCP of HER2-expressing cancer cells using flow cytometry with human PBMCs and PBMC-derived macrophage as effector cells, respectively. Zanidatamab-mediated concentration-dependent ADCC and ADCP of the HER2-expressing NCI-N87 cell line (Fig. 4c, d, respectively), with comparable activity to trastuzumab, pertuzumab and tras + pert. ADCC and ADCP of SK-BR-3 (HER2 3+) and JIMT-1 (HER2 2+) were also evaluated and showed equivalent concentration-dependent activity between with zanidatamab, trastuzumab, pertuzumab and tras + pert (Supplementary Fig. 8b, c).

### Zanidatamab promotes Ab internalization and surface HER2 downregulation

**Fig. 5:**
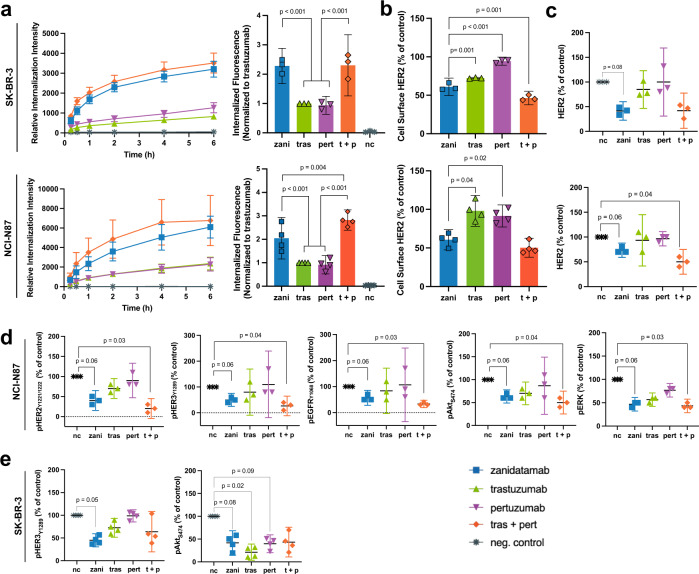
Zanidatamab promotes Ab internalization, surface and total HER2 downregulation and signal inhibition in SK-BR-3 and NCI-N87 cells. **a** Zanidatamab showed increased receptor-mediated internalization compared to trastuzumab or pertuzumab (15 min to 6 h), measured by high content microscopy (*n* = 3 independent experiments). (Right) Zanidatamab conferred significantly greater receptor-mediated internalization compared to trastuzumab or pertuzumab (adjusted *p* < 0.001) in SK-BR-3 cells or NCI-N87 cells (24 h), measured by flow cytometry (*n* = *3* SK-BR-3 *or n* = 4 NCI-N87 independent experiments). A two-way ANOVA controlling for experiment with Bonferroni correction for multiple comparisons was performed, data normalized to trastuzumab, df = 3 for ANOVA and df = 4 for *t*-tests. **b** Zanidatamab showed significantly greater surface HER2 downregulation compared to trastuzumab (*p* = 0.001) and pertuzumab (*p* < 0.001) in SK-BR-3 cells and compared to trastuzumab (*p* = 0.04) and pertuzumab (*p* = 0.02) in NCI-N87 cells, evaluated by flow cytometry. Tras + pert mediated significantly greater HER2 downregulation compared to zanidatamab (*p* = 0.001) in SK-BR-3 cells (*n* = 3 SK-BR-3 or *n* = 4 NCI-N87 independent experiments, two-sample two-sided t tests with Bonferroni correction for multiple comparisons, df = 4). **c** Zanidatamab reduced total HER2 (24 h) when compared to untreated cells (adjusted *p* = 0.08, SK-BR-3; adjusted *p* = 0.06, NCI-N87). **d** Zanidatamab mediated inhibition of pHER2, pHER3, pEGFR, pAKT and pERK (adjusted *p* = 0.06) in NCI-N87 cells compared to untreated cells (24 h). **e** Zanidatamab mediated inhibition pHER3 (adjusted *p* = 0.047) and pAKT (adjusted *p* = 0.082) in SK-BR-3 cells compared to untreated cells (15 min), evaluated by immunoblotting. In **c** (df = 2), **d** (df = 2) and **e** (df = 3), evaluation performed by immunoblotting. In **c** and **d** (*n* = *3)* and in **e** (*n* = 4) independent experiments, one sample two-sided *t*-test compared to untreated cells value of 100% with *p* values adjustment using Benjamini & Hochberg false discovery, comparisons with adjusted *p* values < 0.1 shown. Data in **a** (left) is mean ± SEM, **a** (right), **b**–**e**, mean ± 95% CI. Gating strategy for **a** (right) and **b** is shown in Supplementary Fig. 12e, f. Source data are provided in the Source Data file.

Anti-HER2 biparatopic Abs with increased internalization and receptor degradation over basal rates have been reported^17^, likely facilitated by antigen crosslinking^20–22^. We investigated HER2 internalization in SK-BR-3 (HER2 3+) and NCI-N87 (HER2 3+) cells using high content imaging and flow cytometry. High content imaging showed that zanidatamab treatment resulted in increased levels of receptor-mediated internalization from 15 min to 6 h (Fig. 5a, left). Zanidatamab and tras + pert had similar effect and induced greater internalization compared to trastuzumab or pertuzumab. Internalization evaluated by flow cytometry similarly showed that zanidatamab treatment (24 h) resulted in significantly greater internalization (approximately twofold) than trastuzumab or pertuzumab in SK-BR-3 and NCI-N87 cells (Fig. 5a, right, adjusted *p* < 0.001). Internalization was also evaluated in JIMT-1 cells (HER2 2+), where zanidatamab induced a similar increased internalization compared to trastuzumab (adjusted *p* = 0.007) and pertuzumab (adjusted *p* = 0.02) and was equivalent to tras + pert (Supplementary Fig. 9f).

We then used flow cytometry to measure changes in cell surface HER2 levels following anti-HER2 Ab treatment. Zanidatamab and tras + pert downregulated cell surface HER2 in SK-BR-3 and NCI-N87 (Fig. 5b), BT-474, and JIMT-1 cells (Supplementary Table 5; Supplementary Fig. 9g). Trastuzumab downregulated cell surface HER2 in SK-BR-3 (Fig. 5b), BT-474, and JIMT-1 cells (Supplementary Table 5; Supplementary Fig. 9g), and mediated a weaker effect in NCI-N87 cells. Pertuzumab mediated a weak effect in all cell lines tested. Together these results show that zanidatamab and tras + pert induce robust Ab:HER2 complex internalization with a resultant reduction of HER2 surface levels.

### Zanidatamab reduces total cellular HER2 and inhibits intracellular signaling

Having observed HER2 internalization and reduction in cell surface HER2 levels following zanidatamab treatment, we studied the effects on total HER2 and intracellular signaling levels in SK-BR-3 and NCI-N87 cells by immunoblotting. Following 24 h incubation, zanidatamab and tras + pert reduced total HER2 to similar levels compared to control in SK-BR-3 and NCI-N87 cells (Fig. 5c). No significant reduction in total HER2 levels was observed following trastuzumab or pertuzumab treatment.

Trastuzumab has been reported to inhibit the downstream signaling events, particularly pHER3 and pAKT in high HER2-expressing tumor cells^7^. Therefore, we evaluated intracellular signaling levels (including phosphorylated and total HER2, HER3, EGFR, AKT, and ERK) following zanidatamab treatment (15 min or 24 h) in NCI-N87 and SK-BR-3 cells. Treating NCI-N87 cells with zanidatamab or tras + pert for 24 h caused a reduction in pHER2, pHER3, pEGFR, pAKT, and pERK levels compared to control cells (Fig. 5d, Supplementary Fig. 9a). In SK-BR-3 cells that were treated with Abs for 15 min, only zanidatamab caused a reduction in pHER3, compared to control cells, whereas all of the anti-HER2 Abs tested reduced pAKT levels (Fig. 5e, Supplementary Fig. 9b). A 24-h treatment with zanidatamab resulted in decreased levels of HER2, pHER3 and pAKT in SK-BR-3 cells (Fig. 5c, Supplementary Fig. 9b, d and Supplementary Data 1). We also investigated the effect of zanidatamab treatment in the low-HER2 expressing JIMT-1 (HER2 2+) cell line. Both 15 min and 24-h treatments with zanidatamab reduced the levels of total HER2, pHER3 and pAKT levels similar to what was observed in SK-BR-3 cells (Supplementary Fig. 9c, e Supplementary Data 1, Supplementary Data 2). None of the anti-HER2 Abs caused significant changes in pHER2, HER3, EGFR, pEGFR, EGFR, ERK or pERK levels in SK-BR-3 or JIMT-1 cells at either 15 min or 24 h time points. In summary, zanidatamab treatment reduced total HER2, pHER3, and pAKT levels in HER2-expressing SK-BR-3 and JIMT-1 breast cancer cells, and reduced HER2, pHER2, pHER3 pEGFR, pAKT and pERK in NCI-N87 gastric cancer cells.

### Zanidatamab inhibits ligand-independent and EGF-driven growth of high HER2-expressing tumor cells

**Fig. 6:**
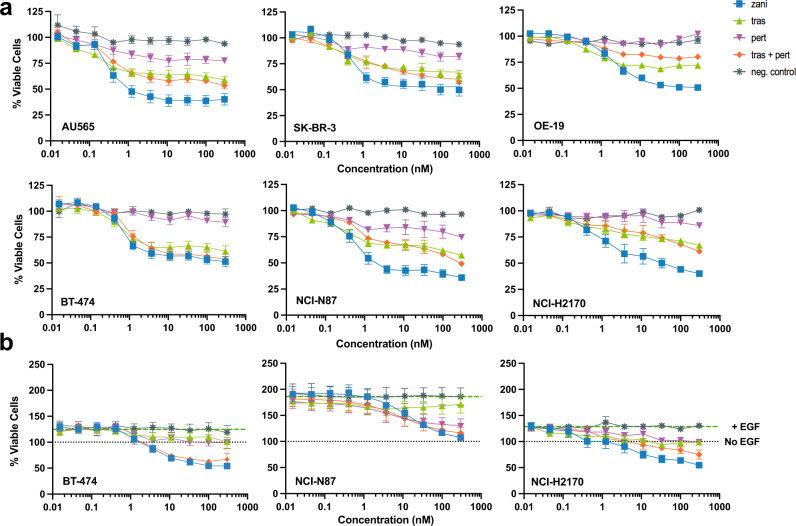
Zanidatamab mediates ligand-independent and EGF-dependent growth inhibition in HER2-expressing tumor cells. **a** Zanidatamab mediated ligand-independent tumor growth inhibition in HER2-expressing tumor cells including AU565, NCI-H2170, OE-19, BT-474, NCI-N87 and SK-BR-3 (HER2 3+). Data are mean ± SEM from *n* = 3 (SK-BR-3), *n* = 4 (OE-19), *n* = 6 (AU565, BT-474, NCI-H2170) or *n* = 12 (NCI-N87) independent experiments. See Supplementary Tables 6, 7 for complete data and pairwise comparisons, respectively. **b** Zanidatamab mediated EGF-dependent tumor growth inhibition in HER2-expressing cell lines, including BT-474, NCI-N87 and NCI-H2170 (HER2 3+). Percent viability is plotted relative to non-treated cells (no EGF, no Ab). Upper horizontal dashed line (green) represents % viable cells upon EGF stimulation (% Viability_(+EGF)_). Lower horizontal dotted line (gray) represents viability of non-treated cells referenced to 100%. Data are  mean ± SEM from *n* = 3 (NCI-N87, NCI-H2170) or *n* = 5 (BT-474) independent experiments. See Supplementary Tables 8, 9 complete data and pairwise comparisons, respectively. Source data are provided in the Source Data file.

We next evaluated whether the effects of HER2 internalization, downregulation, and reduction of phospho-signaling would translate into antiproliferative responses in high HER2-expressing cell lines. We first evaluated ligand-independent antitumor activity of zanidatamab by measuring tumor cell viability following Ab treatment. Zanidatamab mediated concentration-dependent reduction in cell viability in high HER2 expressing breast (BT-474, SK-BR-3, ZR-75-30, HCC2218, AU565, HCC1419), gastric (NCI-N87), esophageal (OE-19), and lung (NCI-H2170), and in the HER2-low MDA-MB-175-VII cancer cell lines (Fig. 6a, Supplementary Table 6). Zanidatamab’s effect was greater or comparable to trastuzumab, pertuzumab, or tras + pert. Pairwise comparisons showed significantly greater growth inhibition (*p* values < 0.03) with zanidatamab compared to tras + pert in ZR-75-30, AU565, NCI-H2170, OE-19, SK-BR-3 and NCI-N87 cells, and compared to trastuzumab in all 10 cell lines, and compared to pertuzumab in all cell lines except MDA-MB-175-VII (Supplementary Table 7).

We then evaluated antitumor activity under conditions driven by epidermal growth factor (EGF)-mediated growth (Fig. 6b). Ligand-mediated HER2-heterodimer formation (*e.g*. HER2-EGFR) drives compensatory signaling and proliferation and is believed to play a role in driving trastuzumab resistance^47^. We first identified cell lines, media conditions and ligand concentrations in which EGF-mediated growth proliferation was observed (Supplementary Table 8). Under the conditions identified, addition of EGF resulted in a 12% to 86% increase in cell proliferation compared to non-ligand treated BT-474, NCI-H2170, NCI-N87, OE-19, and ZR-75-30 cells (Supplementary Table 8). Importantly, zanidatamab mediated a significant concentration-dependent reduction of viability compared to the EGF-treated control in all cell lines tested (Fig. 6b, Supplementary Tables 8 and 9). Zanidatamab mediated significantly greater EGF-driven growth inhibition than trastuzumab or pertuzumab in all 5 cell lines and compared to tras + pert in NCI-H2170 cells. Zanidatamab and tras + pert mediated comparable effects in BT-474, NCI-N87, OE-19, and ZR-75-30 cells (Supplementary Table 9).

In summary, zanidatamab mediates ligand-independent and EGF-driven growth inhibition in several HER2-expressing cell lines including breast, gastric, esophageal and lung cancer. In many cell lines, the effect of zanidatamab was significantly greater than trastuzumab, pertuzumab or tras + pert.

### Zanidatamab promotes potent antitumor activity in high HER2-expressing in vivo models of gastric cancer

**Fig. 7:**
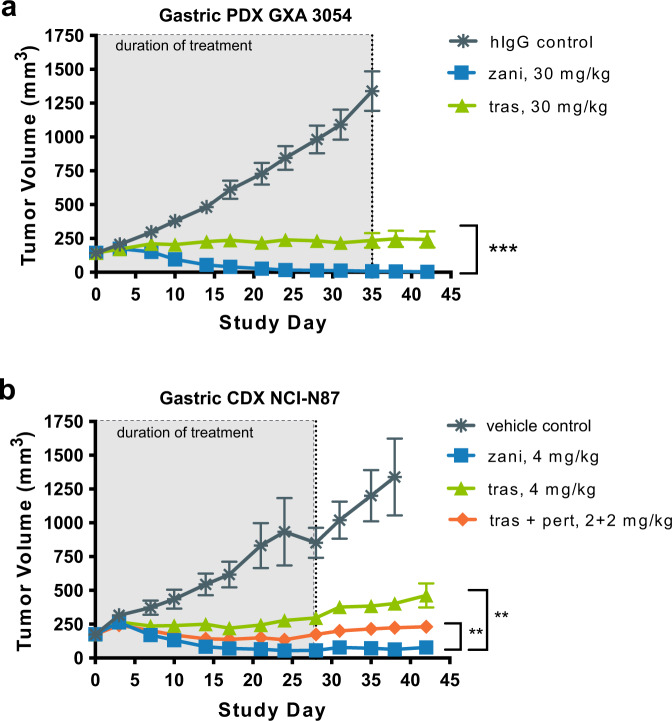
Zanidatamab mediates antitumor activity in HER2-expressing xenograft tumors. **a** Mean tumor volume of patient-derived gastric xenograft model GXA 3054 implanted in nude mice. Tumor bearing mice treated with indicated test articles at 30 mg/kg, IV, twice weekly for five weeks, *n* = 10 per group. ***p value = 3.17e−07. **b** Mean tumor volume of cell-derived gastric xenograft model NCI-N87 implanted in nude mice. Tumor bearing mice treated with; single agent zanidatamab or trastuzumab at 4 mg/kg; or trastuzumab + pertuzumab combination at 4 mg/kg total (2 + 2 mg/kg of each agent), IV, twice weekly for four weeks, *n* = *7* per group. Statistical significance for both models was determined by fitting a linear mixed-effects model on log-transformed tumor growth data and comparing growth rates among all treatment groups, derived from the fitted model. A one-sided *F*-test is used as an omnibus test on the null hypothesis that all growth rates are equal. A post-hoc two-sided Tukey’s test was used to infer differences in growth rates between treatment groups and account for multiple comparisons. **p < 0.01 vs. trastuzumab or tras + pert. In **a** and **b** data are mean ± SEM. Source data are provided as a Source Data file.

The in vivo antitumor activity of zanidatamab and trastuzumab was evaluated in the GXA 3054 model, a high HER2-expressing (HER2 3+; Supplementary Table 10) patient-derived human tumor xenograft (PDX) model of gastric cancer. Antitumor activity was assessed using the rate of tumor volume change relative to control, with tumor growth rate inhibition greater than 100% indicating regression. While both zanidatamab and trastuzumab significantly reduced GXA 3054 growth rate relative to control (307.6% and 110.5%, respectively), zanidatamab treatment induced a greater tumor regression compared to trastuzumab (Fig. 7a).

We next sought to determine whether zanidatamab had greater antitumor activity compared to trastuzumab or a combination of tras + pert in the HER2 3+ NCI-N87 gastric cell line derived xenograft (CDX) model (Fig. 7b; Supplementary Table 10). Zanidatamab (4 mg/kg) induced greater inhibition of tumor growth rate of NCI-N87 xenografts than trastuzumab (4 mg/kg) and tras + pert (2 + 2 mg/kg) (268%, 86%, 155% tumor growth rate inhibition, respectively).

Collectively, these data show that zanidatamab has superior tumor growth inhibition activity than trastuzumab or a combination of tras + pert in gastric cancer models in vivo.

## Discussion

Targeting HER2 has improved outcomes in patients with HER2-positive metastatic breast and gastric/GEJ cancers. However, because the cancers in these patients ultimately progress, there remains a need for anti-HER2 therapies with greater efficacy and differentiated MOAs^3^. To address limitations of the current standard of care, we engineered a biparatopic anti-HER2 Ab, zanidatamab, a human IgG1-like bispecific Ab that recognizes two non-overlapping epitopes (ECD2 and ECD4) and promotes receptor crosslinking. Here, we demonstrated that zanidatamab has unique and enhanced functionalities compared to regulatory approved anti-HER2 monoclonal Abs trastuzumab and pertuzumab. We show that zanidatamab binds HER2 in *trans* and forms distinct and large cell surface HER2 clusters. We hypothesized that zanidatamab’s distinct Ab:HER2 crosslinking leads to potent CDC, an activity that is not observed for trastuzumab, pertuzumab, or tras + pert. Zanidatamab also mediated HER2 internalization and downregulation, inhibition of cell signaling and tumor growth, as well as ADCC, and ADCP. When translated to an in vivo model, zanidatamab showed superior in vivo antitumor activity compared to trastuzumab and tras + pert in HER2-expressing xenograft models.

Biparatopic Abs have been shown to bind tumor cells with increased saturation compared to canonical monospecific Abs^15^ and induce receptor crosslinking^20–22^ leading to cell intrinsic effects including receptor internalization and downregulation, decreased receptor signaling, and growth inhibition. We hypothesized that the biparatopic Ab zanidatamab would increase Ab saturation compared to trastuzumab or pertuzumab and form distinct receptor clusters on HER2-expressing tumor cells that could additionally promote cell extrinsic effects of Fc-mediated cytotoxicity, including CDC.

Evaluation of cell surface binding to HER2-expressing cancer cells by flow cytometry showed that zanidatamab had 1.3 to 1.6-fold higher maximum binding capacity (saturation) compared to the monospecific Abs trastuzumab or pertuzumab. The SPR experiment and cryo-EM-derived model demonstrated the mechanism of HER2 engagement and showed that zanidatamab binds HER2 ECD in *trans*. The *trans* binding of zanidatamab led to the formation of large crosslinked HER2:zanidatamab complexes, or clusters, both in solution as observed by AUC and on-cell surfaces as shown by microscopy experiments. Overall, the formation of clusters, driven by *trans* receptor binding and crosslinking, can be seen as one of the defining characteristics of biparatopic Abs such as zanidatamab.

Using dSTORM, we showed that treatment of HER2-expressing breast cancer cells (SK-BR-3) with zanidatamab induced the formation of large HER2 clusters to a much greater extent than trastuzumab, pertuzumab, or tras + pert treatment. This contrasts with the AUC data, where both zanidatamab and tras + pert promote similarly large oligomers in solution. We believe that the clustering observed by AUC with free HER2 ECD in solution is not fully representative of the binding constraints imposed by HER2 molecules in the cell membrane. Moreover, HER2 is expected to be present as monomers, homodimers, or heterodimers with other HER receptor family members on the cell surface^48^, which is not represented in the AUC study in a homogenous HER2 environment.

The distinct clustering properties of zanidatamab were also observed on a macroscopic level. Zanidatamab promoted the formation of large, polarized clusters of HER2, or caps, a unique property of this biparatopic Ab that was not recapitulated by tras + pert or either Ab alone (Fig. 3a–d). This capping, which was observed at 5 and 15 min following zanidatamab addition to the cells, likely reflects a redistribution of cell surface HER2 into a highly crosslinked receptor aggregate that polarizes to one side of the cell. We hypothesize that the cap structure is a consequence of the nanoscale changes in HER2 organization that were observed by dSTORM.

The absence of HER2 caps at 30 min after zanidatamab addition may be a consequence of the kinetics of receptor internalization following Ab binding. Indeed, we observed zanidatamab internalization as early as 15 min after addition to SK-BR3 cells (Fig. 5a). To our knowledge, we are the first to report the observations of Ab-induced HER2 capping on tumor cells.

Hexameric organization of Ab Fc regions in the Ab-antigen clusters has been shown to be required for optimal CDC activity^33^. Because we had observed that zanidatamab binding induced large cell surface HER2 clusters, we asked whether the oligomeric Fc organization of zanidatamab on the cell surface could facilitate hexamerization and enable complement binding and activation. Indeed, evaluation of CDC in a panel of HER2-expressing cell lines showed that zanidatamab mediated potent antitumor activity in all high HER2-expressing (HER2 3+) cell lines tested. The uniqueness of the zanidatamab-induced HER2 clusters is apparent in the dSTORM imaging (Fig. 3e) and in confocal microscopy (Fig. 3d). Our data suggests that the disappearance of HER2 caps at 30 min in the confocal microscopy does not impact CDC activity since zanidatamab pre-incubation with SK-BR-3 and NCI-N87 cells from 30 min to 1 h did not result in lower CDC activity than was observed for earlier time points. In fact, CDC potency increased with longer duration of zanidatamab:tumor cell pre-incubation (Supplementary Fig. 7c). Increasing the percent of NHS to 80% showed increased CDC with zanidatamab, whereas all other anti-HER2 treatments remained inactive (Supplementary Fig. 7d). Our data suggests that zanidatamab-induced HER2 clusters facilitate a distinct organization of cell surface HER2, and thus a distinctive organization of zanidatamab Fc, that meets the hexameric Fc requirements needed to promote CDC activity. Furthermore, our modeling suggests that zanidatamab bound to HER2 is capable of forming a hexamer (Supplementary Fig. 10) and provides a rationale for why HER2 clustering by zanidatamab appears to provide high avidity docking sites for C1q binding. Additionally, zanidatamab elicited rapid CDC as early as 5 min post serum addition in NCI-N87 and SK-BR-3 cells (Supplementary Fig. 7e), a timescale consistent with large HER2 cluster formation observed in the dSTORM (15 min) and confocal (HER2 caps at 5 and 15 min) data.

Unlike zanidatamab, tras + pert, trastuzumab, or pertuzumab did not mediate formation of HER2 caps, large HER2 clusters, or CDC. In particular, the cluster sizes formed by tras + pert treatment were not larger than those seen with trastuzumab alone. Despite the on-cell flow cytometry binding data that showed both zanidatamab and tras + pert binding with equivalent Ab densities, the tras + pert cluster profiles observed by dSTORM show differences in HER2 engagement among these treatments. Based on the tras + pert cluster profiles observed, it appears that the two antibodies may engage the HER2 receptors independently. Interestingly, the only cell line in which tras + pert elicited weak CDC (NCI-N87), also showed a modest increase in HER2 caps formation following tras + pert binding. While we and others^46^ have shown CDC activity with tras + pert in the presence of rabbit complement serum, it is important to note that tras + pert was inactive in the presence of human complement (Supplementary Fig. 7b, Fig. 4). Due to the capacity of rabbit serum to mediate high background CDC activity compared to human serum (as a result of functional pathway differences, including ability to lyse target cells without substantial C3 deposition^49^ and lack of cross-reactivity to, and inhibition by, human complement regulatory proteins^50^), caution should be taken when drawing conclusions about CDC results using rabbit serum. Overall, the distinct and large HER2 cluster formation we observed with zanidatamab correlated with its CDC activity. We propose that these large HER2 clusters may be sites where a high local density of C1q or Fc receptor binding interactions enables complement-mediated and cellular responses.

The observations we present with zanidatamab, including Ab-induced target receptor capping and clustering, and CDC in tumor cells with high target expression, parallels the observations of approved type I anti-CD20 Abs, such as rituximab. Rituximab binding has been shown to induce CD20 capping on the B-cell surface^51^ and CD20 clustering leading to stronger C1q binding and activation of CDC^52^ in tumor cells with high CD20 expression levels^53^. Moreover, the formation of receptor caps following rituximab binding to CD20 was associated with increased target killing by ADCC^51^. Rituximab has several mechanisms of action, including ADCC and CDC^54,55^. It has been proposed that ADCC and CDC may act cooperatively in heterogeneous tumor cell populations, for example, where complement eradicates cells with high CD20 expression and ADCC eliminates cells with low CD20 expression^56^. We hypothesize that the reasons for the observed differences in CDC with zanidatamab in high *vs*. low-HER2-expressing cell lines may be a function of the HER2 density required for large cluster formation and subsequent C1q engagement, as well as the relative levels of HER2 compared to complement regulatory protein (CD46, CD55, CD59) that inhibit CDC^57^. This requires further investigation and confirmation.

**Fig. 8:**
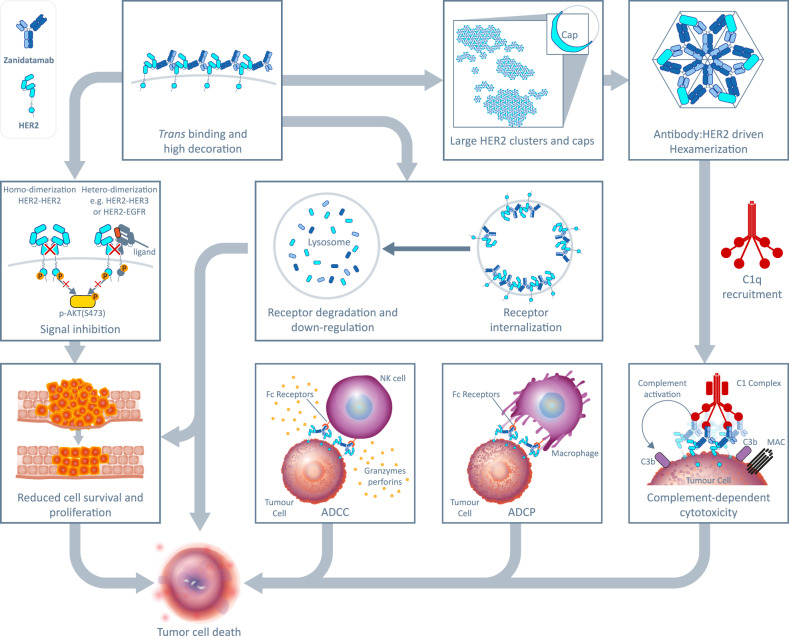
Zanidatamab has multiple mechanisms of action. We hypothesize that the *trans* biparatopic HER2 binding properties of zanidatamab forms the foundation of its multiple mechanisms of action. Zanidatamab binding leads to approximately 1.5-fold increased cell surface Ab saturation as well as to receptor crosslinking. The large cell surface HER2 clusters mediated by zanidatamab engagement facilitate C1q binding, likely by promoting hexamerization, and elicit potent CDC in HER2-high cell lines. In all HER2-expressing cells, including HER2-low tumors, ADCC and ADCP activities were also observed. It is also likely that receptor crosslinking and clustering prevent HER2 homodimerization and heterodimerization with other signaling partners, leading to the ligand-independent and ligand-dependent signal inhibition observed. Additionally, large receptor clusters are not efficiently recycled from early endosomes, culminating in increased receptor internalization and degradation. Together, these mechanisms contribute to the overall effect of tumor cell death and growth inhibition of zanidatamab in vitro and in vivo.

Obligate bispecific Abs, possessing unique functional activities that are not recapitulated by a combination of Abs, represent an emerging approach in antitumor therapy^23^. Distinct clustering properties and potent CDC activity define zanidatamab as an obligate biparatopic Ab since these activities are not recapitulated by tras + pert. To our knowledge, we describe the first example of an obligate biparatopic Ab. Beyond CDC, zanidatamab also promotes a multitude of other antitumor activities that are present in a range of HER2 expressing cells, as summarized in Fig. 8.

We hypothesize that the *trans* biparatopic HER2 binding properties of zanidatamab, which result in increased cell surface Ab saturation, HER2 crosslinking, and the formation of distinctive and large receptor clusters, form the foundation for the diverse MOA observed for this Ab. Extracellularly, these properties lead to ADCC and ADCP activities, and a robust CDC activity that is unique to zanidatamab, compared to trastuzumab, pertuzumab and tras + pert. Zanidatamab-driven crosslinking and clustering likely contribute, at least in part, to ligand-independent and ligand-dependent signal inhibition by preventing HER2 homodimerization and heterodimerization with other signaling partners, a phenomenon previously described for a DARPin biparatopic molecule^18^. Crosslinking impacts the recycling of HER2:zanidatamab complexes, leading to increased receptor internalization and degradation^21^ which, combined with direct inhibition of HER2 signaling pathways, promotes inhibition of growth of HER2-expressing cancer cells. There is likely an interplay among these mechanisms; for example, the opportunity for rapid Fc-mediated effector functions and intracellular mechanisms that together contribute to the overall effect of tumor cell death and growth inhibition. An antitumor framework could exist in zanidatamab-treated tumors, where certain mechanisms predominate in high HER2-expressing tumors and other mechanisms in HER2-low tumors; however, this requires further investigation.

Zanidatamab’s functionalities observed in vitro translate into potent tumor growth inhibition in HER2-expressing gastric cancer xenografts in vivo. In the PDX model GXA 3054, treatment with zanidatamab-induced tumor regression, whereas treatment with trastuzumab did not. In the CDX model NCI-N87, zanidatamab mediated significantly greater antitumor activity compared to trastuzumab or tras + pert, consistent with the in vitro observations, where zanidatamab also showed significantly greater growth inhibition compared to trastuzumab and tras + pert in NCI-N87 gastric cancer cells. Zanidatamab also showed significantly greater in vitro ligand-independent and EGF-driven growth inhibition compared to trastuzumab and tras + pert in cell lines including esophageal, gastric, lung, and breast cancer. (Fig. 6, Supplementary Tables 7, 9) Moreover, cell intrinsic antitumor MOAs including HER2 downregulation, internalization, and pHER3 and pAKT inhibition likely contribute to the antitumor activity observed with zanidatamab in vitro and in vivo. Because mouse models have impaired complement activity compared to human and other mammals^58,59^, the ability of zanidatamab treatment to cause significant regression in the PDX model suggests that the other antitumor mechanisms contribute significantly to the overall effect observed. Our data corroborates the impaired CDC activity with mouse serum reported by others, and zanidatamab did not elicit CDC activity in the high HER2-expressing NCI-N87 with mouse serum (Supplementary Fig. 7f). These data highlight the translational challenges in using mouse models, as they may underestimate antitumor activity driven by CDC. Given those limitations, the relative therapeutic contributions of each of the described mechanisms to zanidatamab’s antitumor activity in vivo are yet to be determined and are currently being evaluated clinically. While findings in rodent models do not always translate to clinical outcomes, the strong antitumor activity of zanidatamab in our PDX model along with its potency in vitro suggests that zanidatamab will function in the clinical setting.

Data from the ongoing phase 1 study (NCT02892123) demonstrate that zanidatamab is well tolerated and has single agent activity in patients with advanced HER2-expressing cancers, including breast, gastric, and biliary tract cancers, that have progressed after standard of care therapies, including administration of HER2-targeted agents such as trastuzumab, pertuzumab, and T-DM1^60,61^. The preclinical data presented herein supports mechanisms of action for the clinical antitumor activity observed with zanidatamab as monotherapy and the potential differentiation of zanidatamab from trastuzumab and tras + pert, which are part of the current standards of care for HER2-positive gastric and breast cancers, respectively. Zanidatamab is currently being evaluated in clinical trials in multiple HER2 overexpressing solid tumors, including a registration-enabling clinical trial in HER2 gene amplified biliary tract cancer (NCT04466891).

In summary, zanidatamab is a biparatopic anti-HER2 Ab with unique and enhanced functionalities compared to trastuzumab, pertuzumab and tras + pert. The strong antitumor activity observed in vivo and in vitro suggests that zanidatamab will show activity in the clinical setting, and perhaps even with greater efficacy than the current anti-HER2 therapies and in indications outside of breast and gastric cancer.

## Methods

All studies were carried out in accordance with all applicable international, national, and local laws and guidelines as indicated. All reagents and suppliers are listed in Supplementary Table 13.

### Cell culture, antibodies, and serum

Cell lines were obtained from ATCC (HCC2218, HCC1954, HCC1419, ZR-75-30, AU565, NCI-2170, BT-474, SK-BR-3, NCI-N87, SKOV3, MCF7), Sigma (OE-19), DSMZ (JIMT-1), Cedarlane (ZR-75-1) and AddexBio (MDA-MB-175-VII, MDA-MB-468). Cell lines were authenticated by supplier and were used from source and expanded for a maximum of 20 passages. Cell lines were routinely spot check tested for mycoplasma, all tests were negative. Cell line supplier and catalog numbers are provided in Supplementary Table 14. Trastuzumab (Herceptin®) and pertuzumab (Perjeta®) were purchased from Crown Bio and Xentech, respectively. Zanidatamab was prepared according to standard manufacturing procedures (CMC Bio). A non-specific IgG1 Ab that binds to respiratory syncytial virus (RSV) protein F, palivizumab, was used as a negative control (neg. control or NC) and was produced under standard expression and purification conditions. Human complement serum, baby rabbit complement serum and pooled murine complement serum were obtained from Cedarlane. Mouse strain serum (male and female Balb/c, male and female CB17 SCID) was collected under protocols that were approved by the Animal Care Committee at University of British Columbia.

### Tumor cell binding by flow cytometry

#### Binding to HER2-expressing tumor cells

For on-cell binding by flow cytometry, tumor cells were resuspended in PBS + 2% FBS and seeded in 96-well plates at 4 × 10^4^ cells/well. Cells were treated with anti-HER2 Abs and incubated at 4 °C for 1 h. Cells were washed and treated with 5 µg/mL AF647-conjugated goat anti-human IgG-Fc secondary Ab in PBS + 2% FBS and incubated at 4 °C for 1 h. After incubation, cells were washed and resuspended in PBS + 2% FBS. The geometric MFI values for the AF647-conjugated secondary Ab was determined using a BD LSRFortessa X-20 flow cytometer, data collection and analysis was performed with FACSDiva v9 and FlowJo v10 software, respectively. Data was analyzed using one site–specific binding analysis in GraphPad Prism 9.2.0 (GraphPad Software, Inc., La Jolla, CA).

In Supplementary Fig. 5d, antibodies were diluted in PBS + 5% FBS and 0.01% sodium azide. SK-BR3 cells were pre-treated with 200 nM of respective anti-HER2 antibodies for 30 min on ice. Cells were washed with PBS + 5% FBS and 0.01% sodium azide and incubated with 7 nM one-armed anti-HER2 ECD1-AF647 for 30 min at 4°C. After washing, cell surface HER2 was detected using LSR II cytometer (Becton Dickinson) and the data were analyzed using FlowJo software (Treestar).

#### C1q and C3b/iC3b/C3dg Detection following anti-HER2 Ab binding

Flow cytometry detection of C1q and C3b/iC3b/C3dg binding was performed as previously described^62^. Tumor cells were suspended in culture media and seeded in 96-well plates at 4 × 10^4^ cells/well at 37 °C, 5% CO_2_ for 24 h. Culture media was removed, and cells were treated with Abs in serum-free media at 37 °C, 5% CO_2_ for 15 min. NHS (Cedarlane) was added at a final concentration of 25% and  plates were incubated at 37 °C, 5% CO_2_ for 15 min. In Supplementary Fig 8a, tumor cells were first incubated with 25% NHS (15 min, 37°C, 5% CO2), followed bytitrated amounts of anti-HER2 or negative control Abs (15 min 37°C, 5% CO2). Following Ab and serum incubation, cells were washed, detached, and resuspended in PBS + 2% FBS and incubated with FITC-conjugated anti-C1q, FITC-conjugated anti-C3/C3b/iC3b/C3dg Abs (clone IH8, specificity C3, C3b, iC3b, C3dg)^63^ or FITC-conjugated anti-C3/C3b/iC3b (clone 5G9, specificity C3, C3b, iC3b)^63^ Abs at 4 °C for 1 h. Cells were washed and resuspended in PBS + 2% FBS. The geometric MFI values for the anti-C1q, anti-C3/C3b/iC3b/C3dg or anti-C3/C3b/iC3b Abs were determined using a BD LSRFortessa X-20 Flow Cytometer using FACSDiva v9 and data analysis performed using FlowJo v10. Data was analyzed by non-linear regression using the four-parameter variable slope "log(agonist) vs. response” model in GraphPad Prism 9.2.0.

### Transmission electron microscopy (TEM)

TEM data collection and processing was performed by NanoImaging Services (San Diego, CA). Samples of zanidatamab were prepared using a carbon grid method and negative stain grids were transferred into a FEI Tecnai T12 electron microscope operating at 120 keV equipped with an FEI Eagle 4k × 4k CCD camera, using a room temperature stage. A total of 216 high magnification (×110,000 and ×67,000) images of zanidatamab were acquired. Generation of the 2D class average images of zanidatamab was performed. Images at magnification of 67,000× were used for particle selection, alignment, and classification.

### Differential scanning calorimetry (DSC)

Antibody thermal stability was measured using DSC as follows: Ab samples in PBS were used for DSC analysis with a VP-Capillary DSC (GE Healthcare, Chicago, IL). At the start of each DSC run, 5 buffer blank injections were performed to stabilize the baseline, and a buffer injection was placed before each sample injection for referencing. Each sample was scanned from 20 to 100 °C at a 60 °C/h rate, with low feedback, 8 s filter, 3 or 5 min pre-scan thermostat, and 70 psi nitrogen pressure. The resulting thermograms were referenced and analyzed using Origin 7 software (OriginLab Corporation, Northampton, MA) to determine melting temperature (Tm) as an indicator of thermal stability.

### Surface plasmon resonance

#### Cis/trans binding

Recombinant human HER2 ECD (Acro Inc.) binding affinity and kinetics were determined by SPR using a Biacore™ T200 (Cytiva, Marlbourough, MA). A goat anti-human Fc polyclonal Ab (Jackson Immunoresearch Inc.) was immobilized on a CM5 sensor chip to 2000 RUs using standard amine coupling, and this surface was used for capture of either zanidatamab, zanidatamab precursor or trastuzumab Abs onto the SPR surface. Increasing densities of these Abs were captured, between 15 and 400 RUs on individual flow cells, by flowing increasing concentrations between between 0.25 and 1 μg/mL for 90 s injections at 10 μL/min. At each capture level, this was followed by HER2 ECD injections between 3.75 and 60 nM over a blank reference and the captured antibody surfaces to generate the binding sensograms. A flow rate of 100 μL/min was used for the HER2 injections to minimize mass transport, with a 200 s association and a long 2000 s dissociation for off-rate determination of the high affinity interactions. The monospecific anti-HER2 Ab trastuzumab was used as a *cis*-binding control capable of only 1:1 binding. Binding parameters were obtained from fitting the double-referenced single-cycle kinetics binding sensorgrams to a 1:1 Langmuir interaction model using the BIAevaluation software on the Biacore™ T200. Average response units (RU) of Ab captured was corrected for molecular weight (MW) by dividing the average RU of captured Ab on the chip by MW of the captured Ab variant (average Ab RU captured corrected by MW = RU captured/MW (kDa) of Ab). Kinetic values that had chi-squared values > 10% of the maximum binding capacity (Rmax), U-values > 25 or t_c_ values (*i.e*. the flow rate-independent component values, a modification of the mass transfer constant) in the range of 10E7 – 10E9 with low SE (indicating mass transport) were excluded from the linear regression analysis. Linear regression analysis was performed using GraphPad Prism 9.4.1.

#### HER2 binding

A Biacore T200 surface plasmon resonance instrument (Cytiva, Inc.) was used to assess the Ab-HER2 interaction. A goat anti-human Fc polyclonal Ab was immobilized to approximately 2000 RUs by amine coupling on a CM5 sensor chip. Zanidatamab or anti-HER2 antibodies were captured onto this surface followed by increasing concentrations of HER2 ECD injected over the captured Ab. Single-cycle binding sensorgrams were double referenced and kinetic binding parameters for the HER2 Ab interaction were determined from a 1:1 binding model using the Biacore™ T200 BIAevaluation software.

### Analytical ultracentrifugation

HER2 ECD at 5×, 2× or 1× molar excess was mixed with trastuzumab, pertuzumab, tras + pert (1:1) or zanidatamab with a final protein concentration of 1 mg/mL in DPBS. Reference buffer and samples of HER2 ECD, Abs, and their respective HER2 ECD complexes were loaded into cells with sapphire windows and two sector, 3 mm charcoal-filled Epon centerpieces and sapphire windows. The samples were centrifuged in an AN50-TI 8-hole rotor in a Beckman-Coulter ProteomeLab XL-I analytical ultracentrifuge at 163,000 × *g* and 20 °C. Absorbance at 280 nm was measured every 4 min and data were processed using a continuous distribution (c(s)) model in Sedfit^64^ with peak integration in GUSSI^65^.

### Cryo-electron microscopy (Cryo-EM)

Cryo-EM was used to evaluate complexes of zanidatamab:HER2 ECD. Using a Vitrobot Mark IV (Thermo Fisher), 3 μL of samples at 0.4 mg/mL were applied to glow-discharged (60 s on carbon side), 2/2 quantifoil holy carbon grids (EMS). The grids were blotted for 1 second (blot force -3) at 100% humidity, 4 °C and immediately plunged into liquid ethane. Grids were imaged on a 300 keV Titan Krios cryo-EM (Thermo Fisher) equipped with a Falcon 3 direct electron detector. Images were taken in linear mode at a calibrated magnification of 59,000×, corresponding to 1.4 Å; per physical pixel with a total dose of 110 electrons/Å^2^. Fully automated data collection was carried out using EPU software (Thermo Fisher) with a nominal defocus range set from -1.5 to -3.5 μm. A total of 524 movie series were collected. Processing was carried out with cryoSPARC v2^66^. 2D classification revealed a core structure corresponding to HER2 bound to the antigen-binding domains of zanidatamab (Fab or scFv), with the reminder of the Ab molecule forming a diffuse background. Ab initio model calculation and subsequent 3D refinement using 27,462 particles generated a reconstruction to 7.6 Å; according to a Fourier shell correlation (FSC) threshold of 0.143. A model of HER2 in complex with zanidatamab’s Fab and scFv based on the structure of HER2 + tras/pert complexes (PDB ID: 1N8Z, 1S78, 6OGE) was fitted into a 3D reconstruction in Chimera^67^. See Supplementary Table 11 for Cryo-EM data collection, refinement and validation statistics.

### Alexa-fluor 647 Ab conjugation

A monovalent anti-HER2 ECD1 antibody, comprising of a single ‘098’ Fab^68,69^ and Azymetric™ IgG1 Fc^38^ was cloned, expressed, and purified. An Alexa-Fluor 647-labeled one-armed anti-HER2 ECD1 Ab was prepared using Alexa-Fluor 647 dye functionalized with an N-Hydroxysuccinimide-ester. Briefly, the Ab was incubated with 21 molar equivalents of N-Hydroxysuccinimide-AF647 (dissolved in DMSO, 10 mM) for approximately 4 h at room temperature protected from light. To remove excess dye following conjugation, the crude reaction mixture was purified over a 7 kDa Zeba desalting column, pre-equilibrated with PBS, pH 7.4. Absorbance values at 280 nm and 650 nm were measured using a Nanodrop 8000 (ThermoFisher) to determine the extent of labeling and concentration of the labeled Ab. See Supplementary Table 12 for fluorophore to antibody ratio determination of AF647-anti-HER2-ECD1 OAA.

### Confocal imaging of HER2 caps and microclusters

SK-BR-3 cells were detached using enzyme-free dissociation buffer (0.5 mM EDTA, 100 mM NaCl, 1 mM glucose, pH 7.4) and warmed to 37 °C in complete RPMI medium before incubating ~5 × 10^5^ cells with 200 nM zanidatamab, trastuzumab, pertuzumab, or tras + pert (1:1 ratio) for 5–30 min at 37 °C in complete RPMI medium. After incubation, an equal volume of 4% paraformaldehyde (PFA) in PBS was added to the cell suspension, yielding a final PFA concentration of 2%. The cells were fixed for 10 min at room temperature. Cells were then washed with PBS and incubated with 74 nM Alexa-Fluor 647-labeled monovalent one-armed anti-ECD1 OAA (Supplementary Fig. 5) for 30 min at 4 °C. Cells were further washed with PBS and settled onto acid cleaned coverslips coated with 0.01% poly-L-lysine. After 15 min, the cells were fixed again using 2% PFA in PBS. Imaging was performed using a spinning disk confocal microscope system (Intelligent Imaging Innovations) consisting of a Zeiss Axiovert 200 M microscope with a 100× NA 1.45 oil Plan-FLUAR objective and a QuantEM 512SC Photometrics camera. 3D optical reconstructions of confocal slices were generated for randomly chosen cells, which were then scored for the presence of HER2 caps or microclusters, as shown in Fig. 3b and Supplementary Fig. 6. For the confocal data, a binomial logistic regression model was fit to the discrete arrangements of “capping” versus “no capping.” Variation across time points and experimental replicates for each treatment was modeled by the following equation:

1 $${{{{{\rm{arrangement}}}}}} \sim {{{{{\rm{experiment}}}}}}+{{{{{\rm{treatment}}}}}}\times {{{{{\rm{time}}}}}}$$

*P* values for pairwise comparisons of model-predicted arrangement were corrected using the Bonferroni method.

### Analysis of nanoscale HER2 clustering by dSTORM

As described above, SK-BR3 cells were detached, resuspended in complete RPMI medium and incubated at 37 °C with 200 nM zanidatamab, trastuzumab, pertuzumab, or tras + pert for 15 min. The cells were fixed with 2% PFA in PBS for 10 min at room temperature, washed with PBS, and then incubated with 148 nM of the AF647-anti-HER2 ECD1 OAA for 30 min at 4 °C. After PBS washes, the cells were settled onto 0.01% poly-L-lysine-coated coverslips and fixed again using 2% PFA in PBS. The coverslips were washed thoroughly with PBS and fiducial markers (ThermoFisher Scientific) and allowed to settle onto the coverslips overnight at 4 °C. Unbound beads were removed by PBS washes and the stuck particles were used for real-time drift stabilization^70^.

dSTORM was performed using a custom-built microscope with a sample drift-stabilization system described previously^70,71^. A 639 nm laser (Genesis MX639, Coherent) was used to excite AF647 and a 405 nm laser (LRD 0405, Laserglow Technologies) was used to reactivate AF647. Both lasers were coupled into an inverted microscope equipped with an apochromatic TIRF oil-immersion objective lens (60×; NA 1.49; Nikon). The emission fluorescence was separated using appropriate dichroic mirrors and filters (Semrock) and detected by EM-CCD cameras (Ixon, Andor). A feedback loop was employed to lock the position of the sample during image acquisition using immobile fiducial markers. Sample drift was controlled to be less than 1 nm laterally and 3 nm axially. Imaging was performed in an oxygen-scavenging GLOX-thiol buffer consisting of 50 mM Tris-HCl, pH 8.0, 10 mM NaCl, 0.5 mg/mL glucose oxidase, 40 μg/mL catalase, 10% (w/v) glucose and 5 mM mercaptoethylamine (Fluka)^72^. The coverslip with attached cells was mounted onto a depression slide filled with imaging buffer and sealed with Twinsil two-component silicone-glue. For imaging, a laser power density of 1 kW/cm^2^ for the 639 nm laser was used to excite AF647. For each sample, 4 × 10^4^ images were acquired for each color channel at 50 frames/s. dSTORM images were reconstructed using custom MATLAB software^71^ that incorporates the image processing pipeline depicted in Figure 3.2 in https://open.library.ubc.ca/soa/cIRcle/collections/ubctheses/24/items/1.0343233. Localization coordinates and their associated uncertainties were computationally determined simultaneously, as described previously by Scurll et al. 2020^41,70,71^ by fitting a function to the intensity profile of each fluorescence event using the MATLAB fit function (https://www.mathworks.com/help/curvefit/fit.html?searchHighlight=fit&s_tid=srchtitle_fit_1) and the error function described by Huang et al.^73^, as described previously^70^. Expressed as standard deviations, lateral uncertainties were typically 10 nm.

Reconstructed SMLM images were analyzed using StormGraph, a graph-based clustering algorithm that can accommodate heterogeneous clusters of diverse sizes and shapes while accounting for localization uncertainties^41,67^. Briefly, for each cell imaged one or more 2 µm × 2 µm ROIs that did not include cell edges were identified for clustering analysis. The clustering of anti-HER2 ECD1-AF647 OAA localizations, with their positional uncertainties taken into account, were then analyzed by StormGraph using the *k* nearest neighbor method with *k* = 15. The minimum cluster size was set to 5 localizations and the other StormGraph parameter, α, was set to 0.05. Clusters containing less than 5 localizations were excluded from the cluster area analysis to avoid uncertainties introduced by randomly distributed localizations, such exclusions accounting for only ~10–15% of localizations in most treatments. Cluster areas, the number of anti-HER2 ECD1-AF647 OAA localizations per cluster, and the mean cluster area per ROI were then compared for the different Ab treatments.

For the dSTORM data, a one-way ANOVA was used to test for a significant treatment effect on cluster area. *P*-values for pairwise comparisons were computed using a two-sided, non-paired t-test for whether treatment of cells with zanidatamab resulted in larger cluster areas. Multiple comparisons were accounted for using the Bonferroni correction.

### Receptor quantification

Receptor density on cells was determined by Quantum 647 MESF (Bang Laboratories) following the manufacturer’s recommended protocol. Briefly, tumor cells (1.5 x 10^5^) were resuspended in PBS + 2% FBS at a pre-determined saturating concentration of labeled Ab and stained on ice. Each cell line was stained with Alexa-Fluor 647-labeled trastuzumab (30 μg/mL) for HER2 receptor quantification. After staining, cells were washed and resuspended in PBS + 2% FBS at 1.6 × 10^6^ cells/mL. Samples were analyzed by flow cytometry and FlowJo software along with Bang Laboratories’ standard beads. Relative receptor density was calculated based on MFI values and the standard curve generated from Bang Laboratories’ standard beads.

### HER2 protein expression by IHC

Untreated formalin fixed paraffin embedded (FFPE) tumor cell line pellets and FFPE tumor samples of GXA 3054 and NCI-N87 were assessed for HER2 protein expression with the FDA-approved HercepTest (Dako, Carpinteria, California). The subjective microscopic scoring of tumor cells is interpreted as 0, 1+, 2+, and 3+, depending on the proportion of tumor cells and the intensity of immunostaining, as described in the package insert.

### HER2 gene amplification by FISH

HER2 gene amplification was determined using the Abbott-Molecular Inc (Des Plaines, Illinois), formerly Vysis Inc (Downers Grove, Illinois), PathVysion HER2 FISH assay, according to the manufacturer’s instructions.

### Complement-dependent cytotoxicity (CDC)

Tumor cells were suspended in serum-free media and seeded in 96-well plates at 5 × 10^4^ cells/well. Abs were added to cells for 15 min at 37 °C, 5% CO_2_. NHS was then added for a final concentration of 25% and plates were incubated at 37 °C, 5% CO_2_ for 4 h or other time points as indicated. CDC was determined using Cell Titer-Glo™ (CTG) luminescent cell viability assay following the manufacturer’s instructions. Percent CDC was calculated relative to non-treated cells. CDC in the presence of heat-inactivated human complement serum served as negative control. The data was analyzed by non-linear regression using the “four-parameter log(inhibitor) vs. response” model in GraphPad Prism 9.2.0.

#### Serum titration CDC assay

Tumor cells were suspended in serum-free media and seeded in 96-well plates at 5 × 10^4^ cells/well. Cells were treated with zanidatamab (10 nM) for 15 min at 37 °C, 5% CO_2_. NHS was added at final concentrations of 80%, 60%, 40% 25%, or 10% in serum-free media. The plates were incubated at 37 °C, 5% CO_2_ for 4 h.

#### Complement CH50 assay

To determine the complement activity of murine serum, samples were tested using a complement CH50 assay (HaemoScan, K002) as per manufacturer’s guidelines. Briefly, serum samples were serially diluted (0-50%) and incubated with erythrocyte suspension in 96-well plates at 37°C, 5% CO_2_ for 30 min. Stop solution was added and samples were centrifuged at 400 × *g* for 10 min, supernatant (100 µL) was transferred to a flat bottom microplate and OD was measured at 415 nm. Percent lysis was calculated relative to maximum and minimum lysis controls (lysis buffer and dilution buffer, respectively).

#### NHS preincubation CDC assay

Tumor cells were resuspended in serum-free media and seeded in 96-well white flat bottom plates at 5 × 10^4^ cells/well. Cells were incubated with pooled NHS for 5 min at 37 °C, 5% CO_2_ in the presence or absence of 0.01 M EDTA. Serial dilutions of antibody were added to the wells such that the final concentration of NHS was 25%. Plates were incubated for 2.5 h at 37 °C, 5% CO_2_. Cell viability was assessed by Cell Titre-Glo® (Promega) luminescence using the BioTek Synergy plate reader.

#### CDC evaluation by flow cytometry

Tumor cells were resuspended in serum-free media and seeded in 96-well v-bottom plates at 5 × 10^4^ cells/well. Cells were incubated with pooled NHS for 15 min at 37 °C, 5% CO_2_ in the presence or absence of 0.01 M EDTA. Serial dilutions of antibody were added to the wells such that the final concentration of NHS was 25%. Plates were incubated for 3 h at 37 °C, 5% CO_2_. Cells were washed and stained with Propidium Iodide (PI) for 10 mins at 4 °C and assessed for viability by flow cytometry using the LSRFortessa flow cytometer (Becton Dickinson). Data were analyzed using FlowJo v10 software (Treestar).

### Ab-dependent cellular cytotoxicity (ADCC) and cellular phagocytosis (ADCP)

For the ADCC assay, human PBMC from 3 donors were rested overnight in RPMI + 10% ultra-low FBS + 100 U/mL recombinant human IL-2. Tumor cells were stained with Cell Tracker™ Green following the manufacturer’s protocol (Invitrogen) and mixed with human PBMC effector cells at an effector:target ratio of 5:1. The cell mixture was added to a serial dilution of anti-HER2 Ab and control treatments at 37 °C and 5% CO_2_ for 4 h. A LIVE/DEAD™ stain was performed with a fixable violet viability dye following the manufacturer’s protocol (Invitrogen). Cells were washed once with PBS, centrifuged at 400 × *g* for 5 min and resuspended in Qsol buffer. Cancer cell cytotoxicity was assessed using the Intellicyte iQue®3 flow cytometer by gating for single, dead cancer cells that expressed green fluorescence compared to the total number of cancer cells that expressed green fluorescence. The percent ADCC (% Cytotoxicity) was determined by subtracting the percent cytotoxicity of the no Ab-treated cocultures from the percent cytotoxicity of the Ab-treated cocultures.

For the ADCP assay, human monocytes were differentiated into macrophages, from human PBMC isolated from 3 healthy donors, by culturing in RPMI + 10% FBS + 10 ng/mL recombinant human M-CSF for 8 days; culture medium was supplemented every 3 days. After 8 days of cultivation, macrophage cellular phenotype (CD14 and CD11b expression) and morphology were confirmed by flow cytometry and microscopy, respectively. Differentiated macrophages were stained with Cytolight Rapid Red and cancer cells were stained with Cell Tracker Green. Cytolight Rapid Red-stained human-macrophages (red) and Cell Tracker^TM^ Green-labeled cancer cells (green) were mixed at an effector (macrophage cell) to target (cancer cell) ratio of 2:1. The cell mixture was added to a serial dilution of anti-HER2 Ab and control treatments and incubated at 37 °C and 5% CO_2_ for 1.5 h. A live/dead stain was performed using the violet fixable viability dye following the manufacturer’s protocol. Cells were washed once with PBS with 2% FBS, centrifuged and resuspended in Qsol buffer. Phagocytic activity was assessed using the Intellicyte iQue®3 flow cytometer by gating for single, live cells that were double positive for green and red fluorescence. ADCP (%) was determined by subtracting the percent double positive cells of the no Ab-treated cocultures from the percent double positive cells of the Ab-treated cocultures. In both ADCC and ADCP assays, data was analyzed by non-linear regression using the “four-parameter variable slope log (agonist) response” model in GraphPad Prism 9.2.0. Data analysis for ADCC and ADCP assay was performed with Forecyt (Intellicyte iQue®3), gating strategies are shown in Supplementary Fig. 12e, d, respectively.

### Internalization

#### Internalization by high content imaging

Tumor cells were labeled with Cell Tracker^TM^ Blue CMAC and seeded in 384-well plates (Perkin Elmer). Abs (50 nM) were fluorescently labeled by coupling to a goat anti-human IgG-Fc Fab-Alexa-Fluor 488 (Jackson ImmunoResearch) at a 1:2 molar ratio overnight at 4 °C. HER2-expressing cancer cells were seeded and incubated overnight at 37 °C in 5% CO_2_ in 384-well plates. Coupled single agent Abs were added to cancer cells at final concentration of 25 nM, and tras + pert at 12.5 nM + 12.5 nM, and cells were incubated at 37 °C for 15 min to 6 h. At endpoint, the wells were washed with PBS and fixed with 4% PFA in PBS. An imaging solution of PBS containing a final concentration of 15 μM Hoechst 33342 and 100 nM anti-Alexa-Fluor 488 (quench) Ab was added and incubated at room temperature for 1 h protected from light. Imaging was performed using an Operetta CLS^TM^ high content analysis system (Perkin Elmer) with confocal acquisition and ×40 magnification water objective. Each field of view included a Z stack of four sections. Image analysis was performed using Harmony® 4.5 software (Perkin Elmer). Briefly, quantification consisted of cytoplasm and membrane region identification. A membrane mask was applied, and spot identification was performed on the remaining cytosol area. Relative internalized intensity was calculated by using the corrected spot intensity – sum per well divided by number of cells for all fields of view per Z stack section. For each well an average of the four Z stack sections was created and averaged with two additional replicate wells.

#### Internalization by flow cytometry

Abs internalization was determined based on the method previously described by Austin et al.^74^. Briefly, Abs were fluorescently labeled by coupling to a goat anti-human IgG-Fc Fab fragment AF488 conjugate (Jackson ImmunoResearch) at a 1:1 molar ratio for 24 h at 4 °C. HER2-expressing SK-BR-3 cancer cells were seeded and incubated overnight at 37 °C in 5% CO_2_ in 48-well plates. For the assessment of internalization, single agent coupled Abs were added to the SK-BR-3 cells at 100 nM, and tras + pert at 50 nM + 50 nM, and incubated at 37 °C for 24 h. To determine quenching efficiency of cell-surface AF488 fluorescence, coupled Abs were added to the cancer cells at 100 nM and incubated at 4 °C for 15 min. For both 37 °C (24 h) and 4 °C (15 min) samples, cancer cells were dissociated, washed, and for a subset of samples, surface AF488 fluorescence was quenched using a rabbit anti-488 Ab (Life Technologies) at 200 nM incubated at 4 °C for 45 min. For all four samples, the geometric mean of AF488 fluorescence was determined by flow cytometry (BD LSRFortessa X-20). Quenching efficiency was first determined with the 4 °C (15 min) sample as

2 $$1-({{{{{\rm{quenched}}}}}}\,{{{{{\rm{AF488}}}}}}\,{{{{{\rm{GeoMean}}}}}}/{{{{{\rm{unquenched}}}}}}\,{{{{{\rm{AF488}}}}}}\,{{{{{\rm{GeoMean}}}}}})$$

Surface fluorescence was then determined with the 37 °C (24 h) as

3 $$({{{{{\rm{unquenched}}}}}}\,{{{{{\rm{AF}}}}}}488\,{{{{{\rm{GeoMean}}}}}} - {{{{{\rm{quenched}}}}}}\,{{{{{\rm{AF}}}}}}488 \,{{{{{\rm{GeoMean}}}}}})/\\ ({{{{{\rm{quenching}}}}}}\,{{{{{\rm{efficiency}}}}}})$$

Finally, the internalized fluorescence at 24 h was calculated by subtracting surface fluorescence (37 °C) from the total unquenched AF488 GeoMean (37 °C). Flow cytometry data collection and analysis was performed with FACSDiva v9 and FlowJo v10 software, respectively

### HER2 cell surface downregulation

Tumor cells were suspended in culture media and seeded in a flat bottom 96-well plate at 4 × 10^4^ cells/well overnight (18–24 h) at 37 °C, 5% CO_2_. Cancer cells were treated with 100 nM single agent Abs, and tras + pert at 50 nM + 50 nM, and incubated for 24 h at 37 °C, 5% CO_2_. Untreated cells were used as a control. Cells were washed with PBS, detached with TrypLE Express and resuspended in PBS + 2% FBS. Cancer cells were incubated with 50 nM of a one-armed anti-HER2 ECD1-AF647 labeled Ab for 1 h at 4 °C. Cells were washed and resuspended in PBS + 2% FBS and analyzed on a BD LSRFortessa X-20 flow cytometer with FACSDiva v9 software, using the APC laser to detect AF-647 fluorescence. All gating was performed wtih FlowJo v10 software, gating on tumor cells based on forward and side scatter profile, followed by determination of the AF-647 geometric mean fluorescence intensity (MFI) for each treatment point. Treatment mediated relative cell surface HER2 was calculated as a percentage of no Ab treatment control and analyzed by non-linear regression using the “four-parameter variable slope log(agonist) response” function in GraphPad Prism 9.2.0.

### Immunoblotting

SK-BR3, NCI-N87 and JIMT-1 cells were pre-treated with 200 nM of anti-HER2 single agent and negative control Abs, and tras + pert at 100 nM + 100 nM, for 15 min or 24 h time points. Cell lysates were generated and analyzed by immunoblotting as described previously^75^. Briefly, cells were lysed with RIPA buffer (30 mM Tris-HCl, pH 7.4, 150 mM NaCl, 1% Igepal, 0.5% sodium deoxycholate, 0.1% SDS, 2 mM EDTA, 1 mM PMSF, 10 µg/mL leupeptin, 1 µg/mL aprotinin, 25 mM β-glycerophosphate, 1 µg/mL pepstatin A, 1 mM Na_3_MoO_4_, 1 mM Na3VO4). Protein concentrations were determined using the BCA protein assay kit (ThermoFisher). Proteins were separated on 8% SDS-PAGE gels in running buffer (25 mM Tris, 192 mM glycine, 0.1% SDS) and transferred to nitrocellulose membranes for 90 min at 100 V in transfer buffer (25 mM Tris, 192 mM glycine, 20% methanol). The membrane blots were blocked with 5% BSA in TBS (20 mM Tris-HCl, pH 8, 150 mM NaCl) for 30 min at room temperature, then incubated overnight at 4 °C with the indicated primary Abs followed by a 1-h incubation at room temperature with horseradish peroxidase-conjugated goat anti-rabbit IgG (Supplementary Table 13). TBS with 0.1% Tween-20 was used for washing the blots. Blots were imaged using a Li-Cor C-DiGit imaging system (Li-Cor, 3600-00) and quantified using Image Studio software. The ratio of signal intensity of the treated to signal intensity of negative control Ab was calculated and normalized to actin within each experiment. The negative control Ab was compared to itself, giving the negative control normalized value of 100%. Each treatment was compared to negative control Ab with a mean comparison value of 100% with a one sample t-test. The p-values were adjusted using Benjamini & Hochberg false discovery rate within each treatment and cell line. For an example of presentation of full scan blots, see the Source Data file.

### Growth inhibition

Tumor cells (1–2 × 10^3^ cells/well) were incubated with a 1:3 serial dilution of Abs (0–300 nM) in 384-well plate (ThermoFisher) for 4–5 days in culture media at 37 °C, 5% CO_2_. After treatment, Ab-mediated growth inhibition was determined using CTG luminescent cell viability assay following the manufacturer’s instructions. Plates were protected from light and incubated at room temperature prior to luminescence measurements using a Synergy H1 microplate reader (BioTek). For ligand-dependent growth inhibition, tumor cells were stimulated with 50 ng/mL EGF (*i.e*. approximate EC_50_ to maximum response range resulting in increased cell viability in cell line panel) in culture media containing 10% FBS. In the ligand-independent and ligand-dependent assays, percent viability was calculated relative to non-treated cells and the data was analyzed by non-linear regression using the “four-parameter log(inhibitor) vs. response” model in GraphPad Prism 9.2.0. With the ligand-independent and EGF-dependent growth inhibition data, two-sample paired *t* tests, where pairing was based on experiment, were performed to compute p-values for therapy comparisons among values of percent of viable cells at 300 nM. *P* values were adjusted for multiple comparisons using Benjamini & Hochberg’s false discovery rate.

### In vivo tumor studies

All animal studies were carried out in accordance with all applicable international, national, and local laws and guidelines and protocols approved by the regional council Committee of Ethics of Animal Experts (Oncotest GmBh) and Institutional Animal Care and Use Committee (CrownBio). The in vivo efficacy of zanidatamab was compared to trastuzumab in the patient-derived (PDX) HER2 3+ gastric cancer model GXA 3054 provided by Oncotest GmbH (Freiburg, Germany). Five- to seven-week-old female NMRI nude mice were housed in individually ventilated cages under a 14–10 h light–dark cycle and 22–26 °C with 40–70% humidity and were subcutaneously implanted with single GXA 3054 tumor fragments (3–4 mm edge length) in PBS from serially passaged xenografts. Animals bearing GXA 3054 tumors were randomly assigned to treatment groups (10 animals per group) when mean tumor volume reached ~140 mm^3^. The in vivo efficacy of zanidatamab, trastuzumab, and tras + pert was evaluated in the HER2 3+ gastric cancer model NCI-N87 (CDX) provided by CrownBio (Beijing, China). Six- to eight-week-old female BALB/c nude mice were housed under a 12 h light–dark cycle at 22–26 °C with 40–70% humidity, and were allowed to access water and food ad libitum. Mice were subcutaneously implanted with a single injection of NCI-N87 tumor suspension of 10^7^ cells in 0.1 mL of PBS with Matrigel (1:1). Animals bearing NCI-N87 tumors were randomly assigned to treatment groups (7 animals per group) when mean tumor volume reached ~175 mm^3^. All tumor volumes were measured twice weekly in two dimensions using a caliper, and the volume was expressed in mm^3^ using the formula:

4 $${V}=({L}\times {W}\times {W})/2$$

where *V* is tumor volume, *L* is tumor length (the longest tumor dimension) and *W* is tumor width (the longest tumor dimension perpendicular to *L*). In both the GXA 3054 and NCI-N87 in vivo studies, animals were euthanized when tumor size exceeded the 2000 mm^3^ volume endpoint, the maximal tumor size was not exceeded except in one case where tumor grew rapidly after previous measurement in vehicle control group of NCI-N87 study. Tumor volumes were graphed using GraphPad Prism 9.2.0.

For in vivo studies a linear mixed-effects model was fit to log-transformed tumor volumes over time in order to compare differences in tumor growth rate between treatment groups^76,77^. Tumor growth rate differences between treatment groups were assessed using a Wald test and the resulting *p*-values were adjusted for multiple tests according to the Tukey procedure. Adjusted *p* values <0.05 were considered statistically significant. Percent tumor growth rate inhibition (% TGRI) for a given treatment group relative to the control was computed using the following expression:

5 $$\%{{{{{\rm{TGRI}}}}}}=[1\mbox{-}({{k}}_{{{{{{\rm{treatment}}}}}}}{/{k}}_{{{{{{\rm{control}}}}}}})]\times 100\%$$

where *k*_treatment_ is the growth rate constant of a given treatment group, and *k*_control_ is the growth rate constant of the control group

### Statistical analysis

Statistical analyses were performed in R v4.2.1 and are described in the corresponding method or figure legends. All studies were repeated at least three times, except for the TEM, AUC and in vivo studies and in few cases noted in supplementary data tables. Meta-analysis on experimental replicate data was performed for CDC, receptor depletion and ligand-independent growth inhibition. The mean estimate from *n* experiments for the meta-analysis was calculated as follows, where *x*_*i*_ is the maximal effect in the *i*th experiment:

6 $$\bar{x}=\frac{1}{n}\mathop{\sum }\limits_{i}^{n}{x}_{i}$$

The mean standard error for the meta-analysis was calculated with the following formula, where *σ*_*i*_ is the standard deviation in the *i*th experiment:

7 $$\overline{{{{{{\rm{SE}}}}}}}=\frac{1}{n}\mathop{\sum }\limits_{i}^{n}\frac{{\sigma }_{i}}{\sqrt{{n}_{i}}}$$

The 95% confidence interval was calculated assuming the mean follows a Normal distribution using:

8 $$\bar{x}\pm 1.96\times \overline{{{{{{\rm{SE}}}}}}}$$

### Reporting summary

Further information on research design is available in the Nature Portfolio Reporting Summary linked to this article.

## Supplementary information

Supplementary Information

Description of Additional Supplementary Files

Reporting Summary

Supplementary Data 1

Supplementary Data 2

Supplementary Movie 1

## Data Availability

The atomic models generated in this study have been deposited into the Protein Data Bank with accession code PDB 8FFJ. The corresponding cryo-EM density maps generated in this study have been deposited into the Electron Microscopy Data Bank with accession numbers EMD-29044. Other atomic models used in this study are available with accession codes 1N8Z, 1S78, 6OGE. The remaining data are available within the Article, Supplementary Information, Source Data file. Source data are provided with this paper.

## Source data

Source Data

## Code availability

The StormGraph code used in this study is available on the Bitbucket database: https://bitbucket.org/jscurll/stormgraph_public2022/src/master/.
