# Supplementary material for: An anti-HER2 biparatopic antibody that induces unique HER2 clustering and complement-dependent cytotoxicity
Source: Nat Commun. 2023 Mar 13;14:1394. doi: 10.1038/s41467-023-37029-3 (PMC10011572; doi:10.1038/s41467-023-37029-3)
Supplement: Supplementary file 1 — Supplementary Information [file 41467_2023_37029_MOESM1_ESM.pdf]

An Anti-HER2 Biparatopic Antibody That Induces Unique HER2 Clustering and Complement-Dependent Cytotoxicity

Nina E.Weisser[1*], Mario Sanches[1], Eric Escobar-Cabrera[1], Jason O'Toole[1], Elizabeth Whalen[1], Peter WY Chan[1], Grant Wickman[1], Libin Abraham[2], Kate Choi[2], Bryant Harbourne[1], Antonios Samiotakis[1], Andrea Hernández Rojas[1], Gesa Volkers[1], Jodi Wong[1], Claire E. Atkinson[4,5,6], Jason Baardsnes[3], Liam J Worrall[4,5,6], Duncan Browman[1], Emma E. Smith[3], Priya Baichoo[1], Chi Wing Cheng[1], Joy Guedia[1], Sohyeong Kang[1], Abhishek Mukhopadhyay[1], Lisa Newhook[1], Anders Ohrn[1], Prajwal Raghunatha[1], Matteo Zago-Schmitt[1], Joseph D. Schrag[3], Joel Smith[1], Patricia Zwierzchowski[1], Joshua M. Scurll[7], Vincent Fung[1], Sonia Black[1], Natalie CJ Strynadka[4,5,6], Michael R. Gold[2], Leonard G. Presta[1], Gordon Ng[8], Surjit Dixit[1]

[1] Zymeworks Inc., 114 East 4th Avenue, Suite 800, Vancouver, BC, Canada.
[2] Department of Microbiology and Immunology, University of British Columbia, Vancouver, BC, Canada.
[3] Human Health Therapeutics Portfolio, NRC-CNRC, Montreal, QC, Canada.
[4] Department of Biochemistry and Molecular Biology, University of British Columbia, Vancouver, BC, Canada.
[5] Centre for Blood Research, University of British Columbia, Vancouver, BC, Canada.
[6] HRMEM Facility, University of British Columbia, Vancouver, BC, Canada.
[7] Department of Mathematics and Institute of Applied Mathematics, University of British Columbia, Vancouver, British Columbia V6T 1Z2 Canada. *Current address*: Department of Urologic Sciences (Vancouver Prostate Centre) and the Institute of Applied Mathematics, University of British Columbia
[8] AbbVie, 1 N. Waukegan Road, North Chicago, Illinois, 60064, United States

* Nina Weisser, nweisser@zymeworks.com (604) 678 1388

Supplementary Results

Kinetic computational model simulates crosslinking ability of biparatopic antibodies

To evaluate the crosslinking potential of biparatopic molecules, we developed a kinetic computational model that simulates the concentrations of the different Ab-receptor complex binding modes at steady state (Supplementary Fig. 1a). The model simulates crosslinked species up to a cluster of 4 receptors with 3 Ab molecules. In this model, arm A has a 10-fold higher affinity to the receptor than arm B. We evaluated different simulation conditions wherein the affinity of either arm of the bispecific was increased by 10, 50, and 100-fold with respect to its starting wild-type affinity.

Using this model, we determined that the maximum bispecific clustering for the precursor biparatopic Ab with the parental scFv or Fab, was 3.92% (Supplementary Fig. 1b). Simulations in which the affinity of the high affinity arm A increased by 100-fold yielded a maximum receptor clustering of 4.99% (Supplementary Fig. 1b). In contrast, increasing the affinity of the lower affinity arm B by 100-fold increased the percentage of clustered receptors to 14.2% (Supplementary Fig. 1c).

CDC

To understand potential differences in CDC activity based on the duration of zanidatamab pre-incubation with tumor cells, we examined CDC activity by measuring cell viability after 5, 15, 30 min, and 1 h zanidatamab:SK-BR-3 or NCI-N87 tumor cell pre-incubations followed by incubation with 25% NHS. All zanidatamab:tumor cells pre-incubation conditions elicited CDC activity; a slight increased potency was observed from 5 (IC_{50} = 23 nM in NCI-N87 and 9.5 nM in SK-BR-3) to 1 h (IC_{50} = 7 nM in NCI-N87 and 3.0 nM in SK-BR-3) (Supplementary Fig. 7c).

To examine the effect of increasing NHS levels on CDC potency, we tested the anti-HER2 antibodies at 10 nM (approximate IC_{50} of zanidatamab, Fig. 4a) in the presence of 0-80% NHS on the NCI-N87 cell line. We observed an increase in cytotoxicity with zanidatamab with increasing percentage of NHS, with up to 100% cytotoxicity being observed at 80% NHS. Trastuzumab, pertuzumab and tras + pert were inactive across all % NHS tested (Supplementary Fig. 7d).

To examine the effect of NHS incubation time on CDC activity induced by zanidatamab (following the standard Ab and tumor cell pre-incubation of 15 min), we evaluated cytotoxicity following NHS incubation at 5, 15, 30 min, and 1 h compared to the standard 4 h incubation. Zanidatamab elicited CDC as early as 5 min in SK-BR-3 and NCI-N87 cells, cell viability decreased with increasing incubation time with maximum effect of 100 % cytotoxicity (0% viability) achieved at 30 min. (Supplementary Fig. 7e).

To examine the effects of incubating tumor cells with NHS prior to Ab addition (compared to the standard CDC assay with Ab pre-incubation with tumor cells for 15 min, followed by NHS addition and incubation for 4 h at 37°C), we evaluated cytotoxicity following NHS and tumor cell pre-incubation (for 5 or 15 min at 37°C for plate and flow cytometry methods, respectively) followed by Ab incubation (2.5 to 3 h) at 37°C. In the plate-based CDC assay, zanidatamab elicited CDC in OE-19, BT-474, SK-BR-3, and NCI-N87 cells similar to that observed with standard CDC assay shown in Fig. 4 (Supplementary Fig. 7f). In the flow cytometry CDC assay, zanidatamab elicited CDC in SK-BR3 and NCI-N87 cells similar to that observed with the standard CDC (Fig. 4a) and NHS pre-incubation CDC (Supplementary Fig. 7f) plate-based assays (Supplementary Fig. 7g). Tras + pert mediated weak CDC in NCI-N87 cells at the highest antibody concentrations tested, and to a much lower extent than

zanidatamab, but did not induce CDC-mediated cell death in any other HER2-expressing cancer cell lines (Supplementary Fig.7f,g). Trastuzumab, pertuzumab and the negative control Abs were inactive in all cell lines tested in both the plate and flow cytometry methods.

C3 fragment deposition

We compared C3 fragment deposition by staining with Abs with specificity for C3b/iC3b/C3dg (clone IH8) or C3b/iC3b (clone 5G9) on SK-BR-3 cells in the presence of NHS. Additionally, with zanidatamab treatment we compared the effect of incubating with NHS first then Ab, compared to Ab then serum (Ab, serum). As observed in NCI-N87 cells (Fig. 4b), zanidatamab mediated the highest C3 fragment deposition (C3b/iC3b/C3dg or C3b/iC3b) compared to tras + pert, whereas trastuzumab or pertuzumab mediated no C3 fragment deposition. Detection of C3b/iC3b/C3dg or C3b/iC3b deposition showed similar results indicating that C3 fragment deposition, as detected with the anti-C3b/iC3b/C3dg Ab, had not fully decayed to C3dg (Supplementary Fig. 8a). Incubating SK-BR-3 cells with zanidatamab followed by NHS (Ab, serum), or NHS incubation followed by zanidatamab showed similar results.

Modeling of zanidatamab:HER2 Hexamerization

To evaluate if zanidatamab bound to HER2 can form hexameric Ab structures, we built a model structure of zanidatamab bound to two copies of HER2 (zanidatamab:2xHER2, Supplementary Fig. 9a) by employing the cryo-EM structure of HER2 in complex with zanidatamab (Fig. 2c, main) and the Fc from the PDB structure 1HZH. The hinge connecting the Fab and scFv of zanidatamab to the Fc was built into the model and geometry optimized. A cyclic hexameric model was built by super-imposing the zanidatamab:2xHER2 complex to

the Fc of 1HZH and applying the crystallographic symmetry from the 1HZH structure (Space Group H 3 2) to the zanidatamab:2xHER2 complex, with the IgG hexamer of zanidatamab bound in a bridging configuration to 6 copies of HER2 (Supplementary Fig. 9b). Interestingly, the orientation of the six bound HER2 molecules are aligned parallel to each other such as may be expected if they were on a cell membrane (Supplementary Fig. 9b, right panel). Also, the hexameric IgG configuration of zanidatamab positions the six Fc domains in a configuration that matches the hexameric Fc-C1q co-complex structure [1].

Supplementary Table 1. Expression yield, DSC melting temperature, Equilibrium Dissociation Constant (K_D), and Kinetic Rate-Constants for One-Armed Antibody (OAA) Constructs.

			Equilibrium Dissociation Constant and Kinetic Rate-Constants by SPR					
**Sample**	**Yield (mg/L)**	**Tm (Fab or scFv)**	**n**	**k_{on} (1/Ms)**	**k_{off} (1/s)**	**K_D (nM)**	**K_D Standard Error**	**K_D-Fold vs.WT**
Parental anti-ECD4 (OAA-scFv)	27	69.4	3	1.0E+05	1.1E-04	1.1	2.94E-10	1.0
Parental anti-ECD2 (OAA-Fab)	20	77.9	12	6.3E+04	8.9E-04	15	1.1E-09	1.0
High Affinity (engineered) anti-ECD2 (OAA-Fab)	22	77.2	5	7.4E+04	1.2E-04	1.7	7.7E-11	8.8

Used abbreviations: association rate constant (k_{on} or ka), dissociation rate constant (k_{off} or kd) and equilibrium dissociation (binding) constant K_D (=k_{off}/k_{on}).

Supplementary Table 2. Equilibrium Dissociation Constant (K_D) and Kinetic Rate-Constants of Zanidatamab Binding to Recombinant Human HER2 ECD by SPR.

Analyte	k_{on} (1/Ms)	k_{off} (1/s)	Apparent K_D (nM)
Human HER2 ECD	7.02E+04	5.22E-05	0.74

Used abbreviations: association rate constant (k_{on} or ka), dissociation rate constant (k_{off} or kd) and equilibrium dissociation (binding) constant K_D (=k_{off}/k_{on}). Representative data shown from $n = 5$ independent experiments.

Supplementary Table 3. Linear Regression R-squared Values for Zanidatamab and Trastuzumab from *cis* vs. *trans* SPR Experiment (Fig. 2A).

Anti-HER2 Antibody	r^2 value		
	**k_{on} (1/Ms) (p-value[1])**	**k_{off} (1/s) (p-value[1])**	**K_D (M) (p-value[1])**
zanidatamab	0.13 (0.2)	0.67 (0.0006)	0.70 (0.0004)
zanidatamab precursor	0.01 (0.6)	0.78 (<0.0001)	0.59 (< 0.0001)
trastuzumab	0.07 (0.3)	0.14 (0.2)	0.37 (0.01)

Used abbreviations: association rate constant (k_{on} or ka), dissociation rate constant (k_{off} or kd) and equilibrium dissociation (binding) constant K_D (=k_{off}/k_{on}).

An anti-human Fc antibody was immobilized by amine coupling on a CM5 sensor chip. Zanidatamab, zanidtamab precursor, or trastuzumab were captured on the anti-human Fc surface at various surface densities, human HER2 extracellular domain analyte was injected over the chip surface and binding parameters determined using a Biacore™ T200. Binding parameters were obtained from fitting the double-referenced single-cycle kinetics binding sensorgrams to a 1:1 Langmuir interaction model using the BIAevaluation software on the Biacore™ T200. Average response units (RU) of antibody captured was corrected for molecular weight (MW) by dividing the average RU of captured antibody on the chip by MW of the captured antibody variant (antibody RU captured corrected by MW = RU captured/MW of antibody). Linear regression analysis was performed using Prism 9.4.1 (GraphPad Software Inc., La Jolla, CA).

[1]An one-sided F-test was used to determine if the slopes were significantly non-zero for zanidatamab, zanidatamab precursor or trastuzumab. Source data are provided in the Source Data file.

Supplementary Table 4. Percent Complement-dependent Cytotoxicity at Maximal Effect Values of Anti-HER2 Abs in Cancer Cell Lines

Cell Line	Cancer Type	HER2 IHC Overall Score[a]	Average HER2 Receptors/Cell[b]	Percent of viable cells remaining following treatment at maximal effect (95% CI)[c]				
				zanidatamab	trastuzumab	pertuzumab	tras + pert	Negative control
JIMT-1	Breast	2+	$5.27x10^5$	Inactive	Inactive	Inactive	Inactive	Inactive
ZR-75-1	Breast	2+	$3.50x10^5$	Inactive	Inactive	Inactive	Inactive	Inactive
MCF7	Breast	1+	$8.25x10^4$	Inactive	Inactive	Inactive	Inactive	Inactive
MDA-MB-175-VII	Breast	0	$1.99x10^5$	Inactive	Inactive	Inactive	Inactive	Inactive

95% CI = 95% confidence interval.

[a]HER2 expression by IHC was determined with HercepTest (Dako, Carpinteria, California). Microscopic scoring of tumor cells was interpreted as described in the package insert [4]

[b] Values are mean from 3 to 6 separate experiments. Values extrapolated from standard curve generated with four bead standards, each with a different Ab binding capacity (ABC), the highest was $5.34x10^5$ ABC/bead

[c] Data is from *n*=3 (JIMT-1, MCF7, MDA-MB-175-VII) or *n*=2 (ZR-75-1) independent experiments. Percent viability (% viability) of treated cells at maximal effect determined by Best-fit Bottom value derived from the 4-parameter log(inhibitor) vs. response sigmoidal curve model fits using GraphPad Prism 9.2.0 (GraphPad Software, Inc., La Jolla, CA)

Supplementary Table 5. Downregulation of Cell Surface HER2 in Cancer Cell Lines

Cell Line	HER2 IHC[a]	Cell Surface HER2 (% of negative control) at Maximal Effect (95% CI)[b]			
		**zanidatamab**	**trastuzumab**	**pertuzumab**	**tras + pert**
BT-474	3+	67 (66, 68)	77 (71, 84)	93 (94, 97)	48 (44, 51)
SK-BR-3	3+	61 (60, 62)	72 (71, 74)	94 (94, 97)	46 (45, 48)
NCI-N87	3+	61 (47, 74)	98 (78, 118)	92 (77, 106)	50 (38, 63)
JIMT-1	2+	27 (22, 32)	35 (32, 38)	71 (70, 73)	24 (21, 28)

95% CI = 95% confidence interval.

[a] HER2 expression by IHC was determined with the HercepTest (Dako, Carpinteria, California). Microscopic scoring of tumor cells was interpreted as described in the package insert [4]

[b] Values are mean and 95% CI from n=3 (BT-474, SK-BR-3, JIMT-1) or n=4 (NCI-N87) independent experiments. Source data are provided in the Source Data file.

Supplementary Table 6. Summary of Ligand-Independent Growth Inhibition for Zanidatamab, Trastuzumab, Pertuzumab, and the Combination of Trastuzumab Plus Pertuzumab in Cancer Cell Lines

Cell Line	HER2 IHC[a]	Percent Viable Cells Remaining at 300 nM (95% CI)[b]				
		zanidatamab	trastuzumab	pertuzumab	tras+pert	Negative control
HCC2218	3+	27 (25, 30)	58 (54, 63)	67 (60, 73)	29 (26, 32)	100 (88, 112)
HCC1419	3+	66 (56, 76)	82 (77, 87)	97 (93, 101)	69 (64, 75)	96 (93, 99)
ZR-75-30	3+	31 (26, 35)	51 (48, 55)	68 (63, 73)	39 (35, 42)	90 (83, 97)
AU565	3+	33 (32, 35)	64 (60, 67)	78 (67, 88)	57 (54, 59)	93 (84, 102)
NCI-H2170	3+	40 (38, 43)	67 (61, 73)	86 (81, 91)	61 (58, 64)	101 (96, 105)
OE-19	3+	51 (46, 56)	72 (69, 75)	102 (99, 106)	80 (76, 85)	96 (88, 105)
BT-474	3+	51 (46, 56)	61 (56, 66)	89 (84, 95)	53 (49, 58)	97 (88, 106)
SK-BR-3	3+	50 (47, 52)	65 (62, 67)	82 (80, 85)	58 (56, 59)	94 (91, 97)
NCI-N87	3+	36 (33, 39)	57 (52, 63)	75 (69, 80)	49 (45, 53)	97 (87, 107)
MDA-MB-175-VII	0	20 (18, 23)	62 (60, 65)	25 (23, 28)	22 (20, 25)	75 (69, 80)

95% CI = 95% confidence interval.

[a] HER2 expression by IHC was determined with the HercepTest (Dako, Carpinteria, California). Microscopic scoring of tumor cells was interpreted as described in the package insert [4]

[b] Values are mean percent viable cells at 300 nM and 95% CI from *n*=3 (SK-BR-3, MDA-MB-175VII), *n*=4 (HCC1419, OE-19), *n*=5 (HCC2218), *n*=6 (ZR-75-30, AU565, NCI-H2170, BT-474) or *n*=12 (NCI-N87) independent experiments.

Source data are provided in the Source Data file.

Supplementary Table 7. P-values for Pairwise Comparisons of Percent Viable Cells Remaining Estimates for Ligand-Independent Growth Inhibition in Cancer Cell Lines

Cell Line	df	Comparison[a]		
		zanidatamab - trastuzumab	zanidatamab – pertuzumab	zanidatamab - tras + pert
HCC2218	4	p < 0.001	p < 0.001	p = 0.4
HCC1419	3	p = 0.03	p = 0.005	p = 0.4
ZR-75-30	5	p < 0.001	p < 0.001	p = 0.02
AU565	5	p < 0.001	p < 0.001	p < 0.001
NCI-H2170	5	p < 0.001	p < 0.001	p < 0.001
OE-19	3	p = 0.004	p < 0.001	p = 0.003
BT-474	5	p = 0.02	p < 0.001	p = 0.4
SK-BR-3	2	p = 0.01	p = 0.003	p = 0.02
NCI-N87	11	p < 0.001	p < 0.001	p < 0.001
MDA-MB-175-VII	2	p = 0.002	p = 0.05	p = 0.3

df = degrees of freedom

[a] Two-sample two-sided paired t tests, where pairing was based on experiment, were performed to compute p-values for treatment comparisons among percent viable cells remaining following 300 nM antibody treatment. P-values were adjusted for multiple comparisons using the FDR correction. Comparisons with p-values < 0.05 were considered significantly different.

Supplementary Table 8. Summary of EGF-Dependent Growth Inhibition for Zanidatamab, Trastuzumab, Pertuzumab, and the Combination of Trastuzumab Plus Pertuzumab in Cancer Cell Lines

Cell Line	HER2 IHC[a]	% Viability (+EGF)[b]	Percent Viable Cells Remaining at 300 nM (95% CI)[c]				
			zanidatamab	trastuzumab	pertuzumab	tras + pert	Negative control
BT-474	3+	125 (119, 130)	54 (50, 59)	101 (95, 106)	99 (90, 107)	67 (55, 79)	119 (111, 127)
NCI-H2170	3+	129 (124, 133)	55 (50, 60)	99 (91, 107)	99 (91, 107)	75 (68, 82)	131 (116, 145)
NCI-N87	3+	186 (183, 189)	108 (102, 114)	171 (167, 176)	129 (126, 133)	116 (112, 120)	186 (175, 197)
OE-19	3+	112 (110, 115)	68 (65, 70)	88 (86, 90)	99 (95, 103)	86 (83, 89)	110 (104, 115)
ZR-75-30	3+	163 (156, 169)	30 (23, 37)	69 (63, 74)	61 (54, 68)	33 (32, 33)	141 (124, 158)

95% CI = 95% confidence interval.

[a] HER2 expression by IHC was determined with the HercepTest (Dako, Carpinteria, California). Microscopic scoring of tumor cells was interpreted as described in the package insert [4]

[b] Effect of EGF stimulation in the absence of test article. Values are the mean percent viable cells from at least two replicates. Untreated cells are defined as 100%

[c] Values are the mean percent viable cells remaining at 300 nM from n=2 (OE-19), n=3 (ZR-75-30, NCI-H2170, NCI-N87) or n=5 (BT-474) independent experiments.

Source data are provided in the Source Data file.

Supplementary Table 9. P-values for Pairwise Comparisons of Percent Viable Cells Remaining Estimates for EGF-Dependent Growth Inhibition in Cancer Cell Lines

Cell Line	df	Comparison[a]			
		zanidatamab - EGF	zanidatamab - trastuzumab	zanidatamab - pertuzumab	zanidatamab - tras + pert
BT-474	4	$p < 0.001$	$p = 0.001$	$p = 0.002$	$p = 0.05$
NCI-H2170	2	$p = 0.005$	$p = 0.01$	$p = 0.01$	$p = 0.03$
NCI-N87	2	$p = 0.005$	$p = 0.006$	$p = 0.02$	$p = 0.1$
OE-19	1	$p = 0.03$	$p = 0.05$	$p = 0.05$	$p = 0.06$
ZR-75-30	2	$p = 0.005$	$p = 0.01$	$p = 0.02$	$p = 0.4$

df = degrees of freedom

[a] Two-sample two-sided paired t tests, where pairing was based on experiment, were performed to compute p-values for treatment comparisons among percent viable cells remaining following 300 nM antibody treatment. P-values were adjusted for multiple comparisons using the FDR correction. Comparisons with p-values < 0.05 were considered significantly different.

Supplementary Table 10. HER2 IHC, FISH in GXA 3054 and NCI-N87 Xenograft Tumor Samples

Model	HER2 IHC Score[a]	HER2 Gene Amplification Ratio[b]
GXA 3054	3+	10
NCI-N87	3+	9

[a]HER2 expression by IHC was determined with the HercepTest (Dako, Carpinteria, California). Microscopic scoring of tumor cells was interpreted as described in the package insert [4]

[b] HER2 gene amplification was determined using the Abbott-Molecular Inc (Des Plaines, Illinois), PathVysion HER2 FISH assay, according to the package insert. A ratio of HER2 gene amplification is calculated by an average HER2 gene copy number divided by the average CEP17, a HER2 gene ratio greater than or equal to 2.0 were considered ‘amplified.

Supplementary Table 11. Cryo-EM Data Collection, Refinement and Validation Statistics

	Zanidatamab-HER2 EMDB-29044/PDB 8FFJ
**Data collection and processing**	
Magnification	59,000
Voltage (kV)	300
Electron exposure (e–/Å^2)	120
Defocus range (μm)	1.5 – 3.5
Pixel size (Å)	1.4
Symmetry imposed	C1
Initial particle images (no.)	141,556
Final particle images (no.)	27,462
Map resolution (Å)<br>FSC threshold	7.5<br>0.143
**Refinement**	
Initial model used	In silico model based on 1N8Z, 1S78, 6OGE; rigid body docked
Map sharpening *B* factor (Å^2)	-534
Model composition<br>Non-hydrogen atoms<br>Protein residues	 8970<br>1170
B factors (Å^2)	Not refined
R.m.s. deviations<br>Bond lengths (Å)<br>Bond angles (°)	 0.014<br>2.125
Validation<br>MolProbity score<br>Clashscore<br>Poor rotamers (%)	 1.84<br>1.19<br>3.41
Ramachandran plot<br>Favored (%)<br>Allowed (%)<br>Disallowed (%)	 88.10<br>10.52<br>1.38

Supplementary Table 12. Fluorophore to Antibody Ratio Determination of AF647-anti-HER2-ECD1 OAA by UV-Vis

Lot	Absorbance λ = 650 nm PL = 0.1 cm	EC_{AF647} ($M^{-1}cm^{-1}$)	% free NHS-AF647 (HPLC-SEC-fluor)	[AF647] (uM)	Absorbance λ = 280 nm PL = 0.1 cm	EC_{OAA} ($M^{-1}cm^{-1}$)	MW (g/mol)	OAA (uM)	Fluor to Ab Ratio
1	1.57	239,000	4.75%	66.2	0.161	157,455	98,876	7.4	8.9
	1.71				0.168				
	1.70				0.171				
2	4.17	239,900	5.06%	165	0.48	157,455	98,876	20.6	8.1
	4.20				0.47				
	4.08				0.39				

Supplementary Table 13. Assay and Reagents Used in this Study

Assay and Reagent (dilution, concentration, %)	Catalog #	Supplier
**Cell culture, antibodies, serum**		
• Normal human serum	ICSER	Cedarlane
• Baby rabbit complement serum	C12CA	Cedarlane
• Pooled murine complement serum	CLC3000	Cedarlane
• Mouse strain serum	NA	University of British Columbia
**Tumor cell binding by flow cytometry and Receptor depletion**		
• Phosphate buffered saline	10010	Gibco
• Fetal bovine serum, 2%	12483	Gibco
• AF647 conjugated-goat anti human IgG-Fc Ab (5 µg/mL)	109-605-098	Jackson Labs
• FITC-conjugated anti-C1q Ab (1:10)	Ab4223	Abcam
• FITC-conjugated anti-C3/C3b/iC3b/C3dg Ab, clone IH8 (5 µg/mL)	CL7637F	Cedarlane
• FITC-conjugated anti-C3/C3b/iC3b Ab, clone 5G9 (5 µg/mL)	CL7652F	Cedarlane
**Surface plasmon resonance**		
• CM5 sensor chip	BR100530	Cytiva Life Sciences
• Goat anti-human Fc polyclonal Ab	109-005-098	Jackson ImmunoResearch Labs
• Recombinant human HER2 ECD	BMS362	eBioscience
**Alexa-fluor 647 antibody conjugation**		
• Alexa-fluor 647 dye	A20006	Thermofisher
• 7 kDa Zeba desalting column	89882	Thermofisher
• Nanodrop 8000		Thermofisher
**Confocal imaging**		
• Acid cleaned coverslips	0107032	Marienfeld
• Poly-L-lysine (0.01%)	P4707	Sigma-Aldrich
**dSTORM**		
• Fluorescent beads,100 nm	F8799	ThermoFisher Scientific
• glucose oxidase (0.5 mg/mL)	G2133	Sigma-Aldrich
• catalase (40 ug/mL)	C100-50mg	Sigma-Aldrich
• Silicone glue	13001000	Picodent
**Immunoblotting**		
• Igepal, 1%	18896	Sigma-Aldrich
• Nitrocellulose membranes	1620115	BIORAD
• pHER3 (1:1000)	2842	Cell Signaling Technology
• HER3 (1:1000)	12708	Cell Signaling Technology
• pEGFR (1:1000)	2234	Cell Signaling Technology
• EGFR (1:500)	2232	Cell Signaling Technology
• pHER2 (1:1000)	2243	Cell Signaling Technology
• HER2 (1:1000)	2165	Cell Signaling Technology
• pAKT (1:1000)	8599	Cell Signaling Technology
• AKT (1:1000)	4691	Cell Signaling Technology
• pERK (1:1000)	4370	Cell Signaling Technology
• ERK (1:1000)	4695	Cell Signaling Technology
• beta actin (1:1000)	PA5-16914	Invitrogen
• HRP-conjugated goat anti-rabbit IgG (1:3000)	170–6515	Bio-Rad
• ECL detection	AC2010	Azure Biosystems
• Li-Cor C-DiGit imaging system	3600-00	Li-Cor
**ADCC, ADCP**		
• LIVE/DEAD Fixable Violet Dead Cell Stain	L34964	ThermoFisher Scientific
• Recombinant human M-CSF, 10 ng/ml	216-MC-025	R&D Systems
• Cytolight Rapid Red	4706	Sartorius Stedim North America
• CellTracker Green CMFDA Dye	C7025	ThermoFisher Scientific
• Human Recombinant IL-2	202-IL	R&D Systems
**Internalization**		
• Goat anti-human IgG-Fc Fab fragment AF488 conjugate	109-547-008	Jackson ImmunoResearch
• Rabbit IgG anti-488 Ab (200 nM)	A-11094	Life Technologies

NA-Not applicable; HRP-horseradish peroxidase

Supplementary Table 14. Cell Line Source

Cell Line	Vendor	Catalogue Number
HCC2218	ATCC	CRL-2343
HCC1954	ATCC	CRL-2338
HCC1419	ATCC	CRL-2326
ZR-75-30	ATCC	CRL-1504
AU565	ATCC	CRL-2351
NCI-2170	ATCC	CRL-5928
BT-474	ATCC	HTB-20
SK-BR-3	ATCC	HTB-30
NCI-N87	ATCC	CRL-5822
SKOV-3	ATCC	HTB-77
MCF7	ATCC	HTB-22
OE-19	Sigma	96071721-1VL
JIMT-1	DSMZ	ACC 541
ZR-75-1	Cedarlane	CRL-1500
MDA-MB-175-VII	AddexBio	C0006009
MDA-MB-468	AddexBio	C0006003

Supplementary Figures

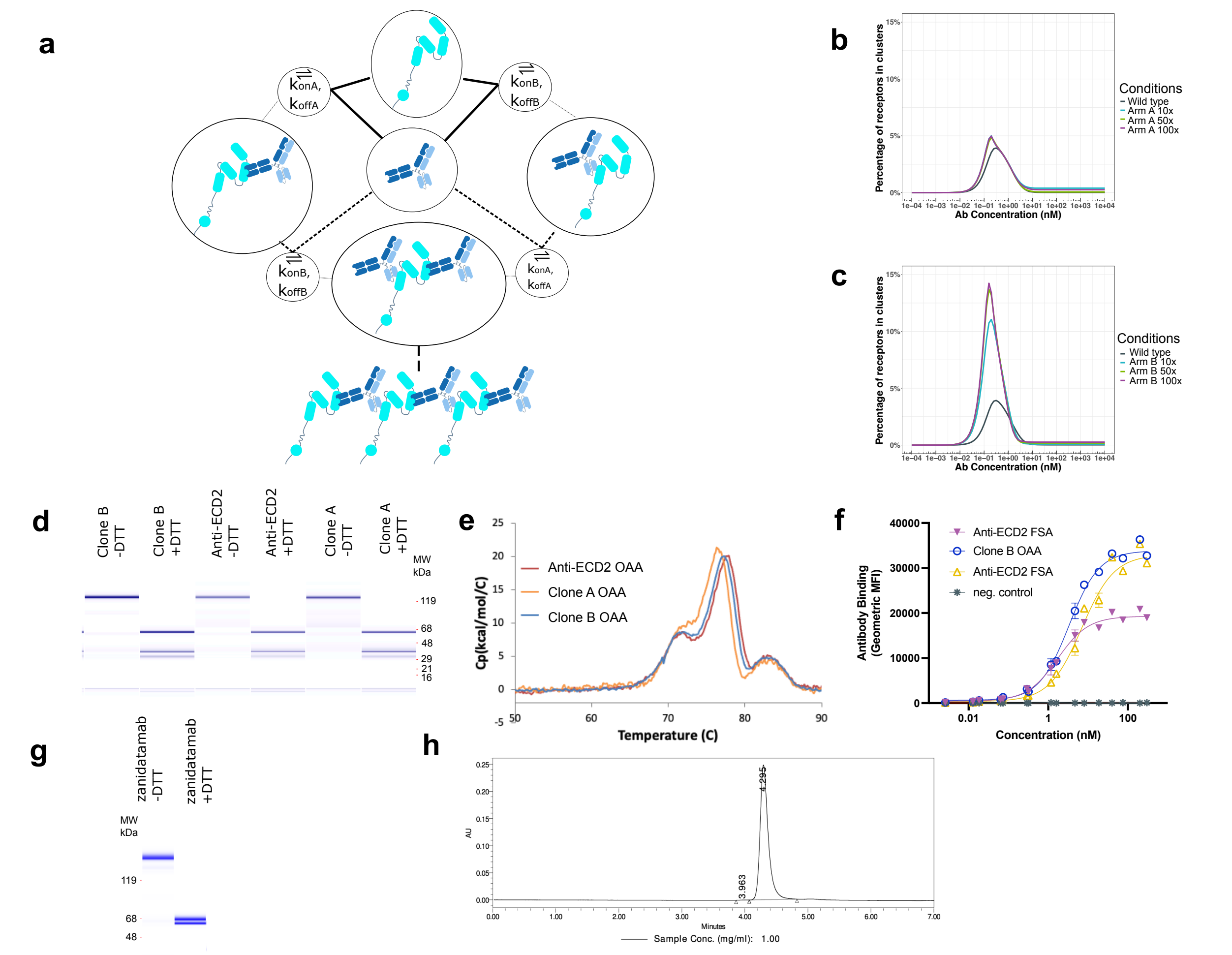

	RT	% Area	Area	Height
1	3.963	0.07	1514	242
2	4.295	99.93	2239974	248793

Supplementary Fig. 1. Kinetic modeling and engineering data of anti-ECD2 paratope. **(a)** Schematic of the computational kinetic model. A biparatopic molecule with an arm A (green) and arm B (blue) binds to two different epitopes of HER2 (cyan). The model simulates crosslinked species up to a cluster of 4 receptors with 3 antibody molecules. In this model arm A has a 10-fold higher affinity to the receptor than arm B. **(b)** Percentage of receptors in clusters in different simulation conditions where the affinity of the higher affinity arm A is increased by 10, 50 and 100-fold with respect to its starting wild-type affinity. **(c)** Percentage of receptors in clusters in different simulation conditions where the affinity of the lower affinity arm B is increased by 10, 50 and 100-fold with respect to its starting wild-type affinity. **(d)** Capillary Electrophoresis-SDS for the one-armed antibody (OAA) anti-ECD2 Fab constructs after protein A and SEC purification in the absence (-DTT) or presence (+DTT) of a reducing agent. The bands in the non-reducing lanes correspond to the monovalent OAA constructs, and the bands in the reducing lanes correspond to the two heavy chains and the light chain **(e)** DSC thermograms

for anti-HER2 OAA constructs. The first transition (lower temperature) corresponds to the CH2 domain, the second transition to the Fab, and the third transition to the CH3 domain. The heights of the peaks were normalized using the CH3 transition of the original precursor anti-ECD2 OAA as reference. The effect of the mutations is seen as shifts in the Fab transition. **(f)** Dose dependent binding of clone B OAA, the original WT anti-ECD2 OAA (anti-ECD2 OAA), the WT full-sized parental antibody (FSA; anti-ECD2 FSA), and a negative control Ab to SK-BR-3 cells. Data are mean ± SEM from $n=3$ independent experiments. **(g)** Capillary electrophoresis-SDS of zanidatamab in the absence (-DTT) or presence (+DTT) of a reducing agent. The band in the non-reducing lane is consistent with an intact Fab-scFv-Fc construct, and the three bands at the reducing lanes correspond to the scFv-Fc, intact heavy chain and intact light chain. **(h)** Ultra performance liquid chromatography-size exclusion chromatography (UPLC-SEC) analysis of zanidatamab shows a well-behaved sample with >99.9% mono-dispersity. The kinetic model was developed and simulated in MATLAB version R2019b, using the Simbiology package and data plotted using the ggplot2 visualization package in R. Source data are provided as a Source Data file.

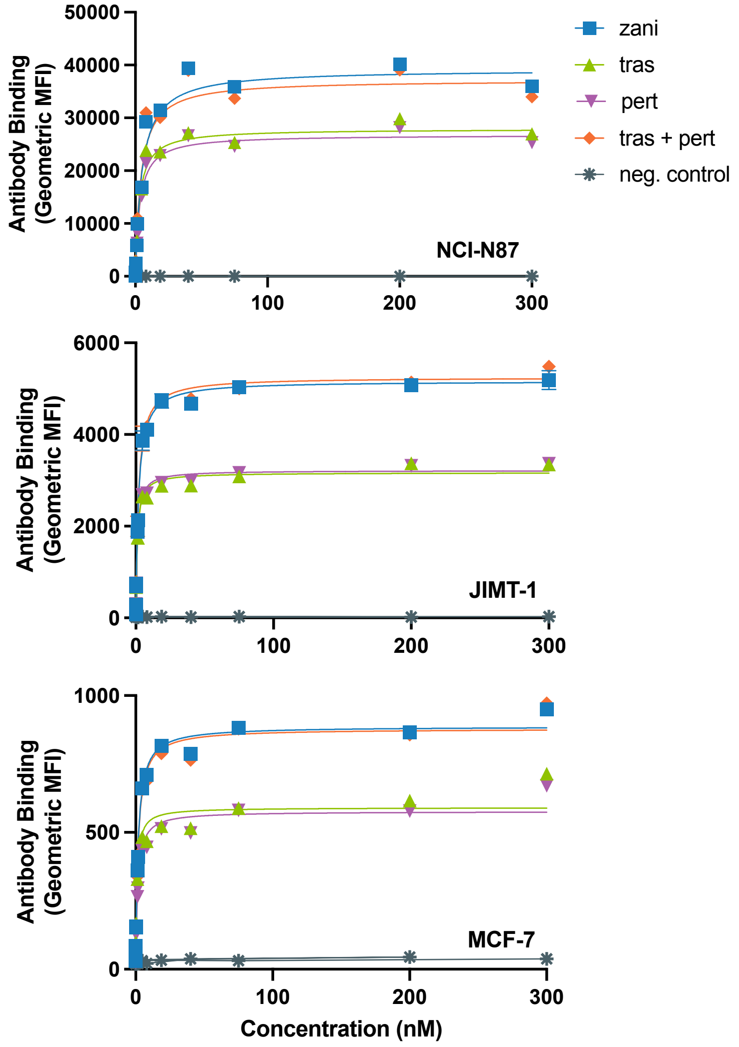

Supplementary Fig. 2. Zanidatamab binds HER2-expressing tumor cells with greater Ab saturation than trastuzumab or pertuzumab. Zanidatamab binds with increased Ab density in HER2-expressing NCI-N87 (HER2 3+), JIMT-1 (HER2 2+) and MCF7 (HER2 1+) compared to trastuzumab or pertuzumab. Flow cytometry was used to quantify the binding of zanidatamab, trastuzumab, pertuzumab, and tras + pert (1:1) to the HER2-expressing tumor cells. Data are mean ± SEM from *n=3* (NCI-N87, JIMT-1) or mean from *n= 2* (MCF-7) independent experiments. Gating strategy is shown in Supplementary Fig. 11a. Source data are provided in the Source Data file.

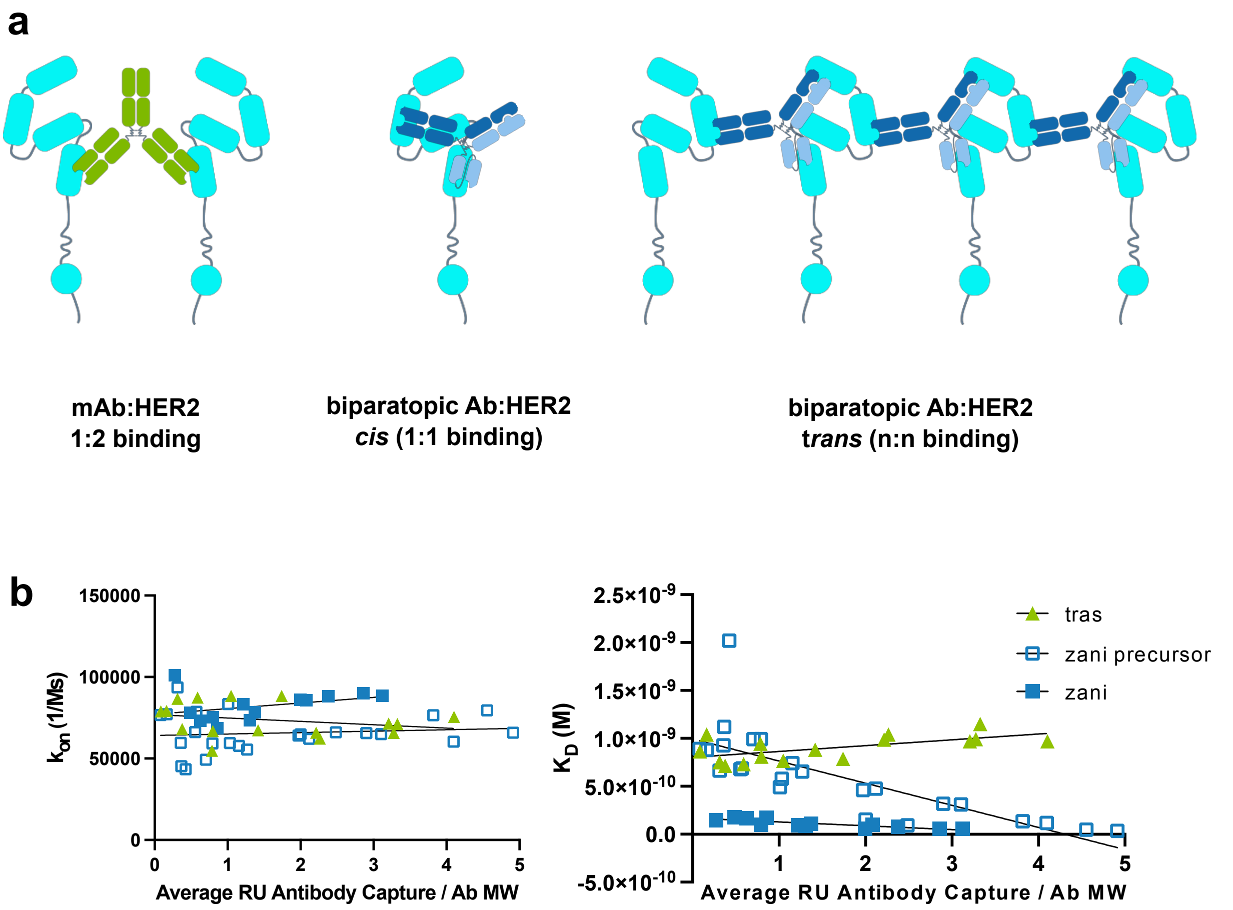

Supplementary Fig. 3. (a) Graphical illustration showing a monoclonal Ab and a putative biparatopic Ab *cis* and *trans* HER2 binding stoichiometries. (Left) Monoclonal antibody, trastuzumab, binding to HER2 ECD4 in a 1:2 Ab to HER2 ratio. (Middle) Representation of zanidatamab binding HER2 in a *cis* orientation, where HER2 ECD2 and ECD4 are engaged by a single zanidatamab molecule in a 1:1 Ab:HER2 binding stoichiometry. (Right) Representation of zanidatamab binding HER2 in a *trans* orientation, where ECD2 and ECD4 on a single HER2 molecule are engaged by two different zanidatamab molecules in a crosslinked *n:n* binding stoichiometry. (**b)** HER2 binding k_{on} and apparent K_D observed over varying Ab densities as determined by SPR. Average response units (RU) of Ab captured was corrected for molecular weight (MW). Data from two independent experiments are shown. Linear regression analysis was performed using Prism 9.4.1. Source data are provided in the Source Data file.

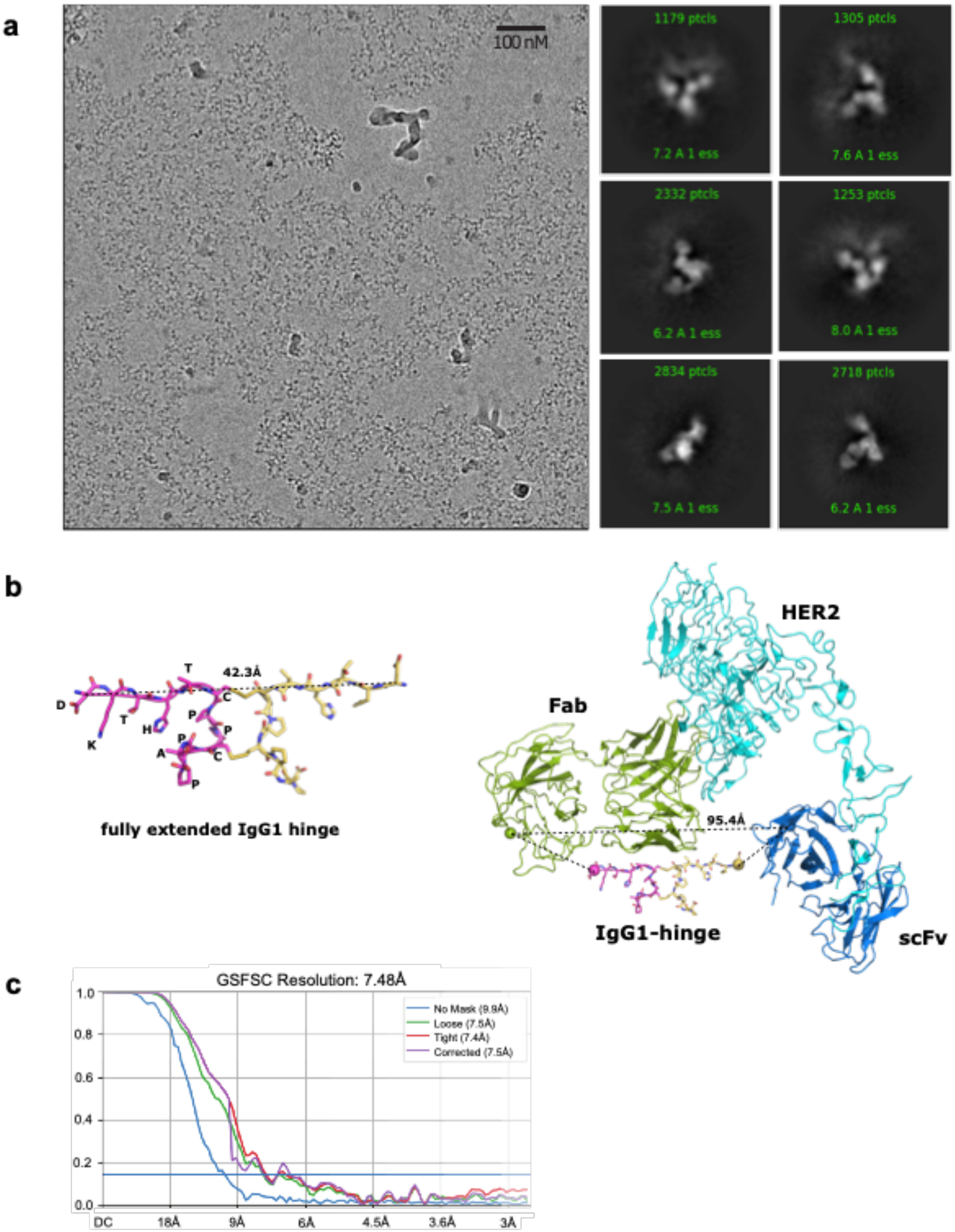

Supplementary Fig. 4. (a) Representative cryoEM micrograph out of 524 movies collected (left), and 2D class average images of HER2:zanidatamab complexes (right). Three independent data collections were carried out with similar results. (**b**) Model of a fully extended IgG1 hinge dimer $(DKTHTCPPCPAP)_2$ spanning a maximum distance of 42.3Å (left). Atomic model of HER2 (cyan) in complex with zanidatamb Fab (green) and scFv (blue) (right). The distance

between the Fab and scFv C-termini is shown in the figure (95.4Å). This distance exceeds the length of the IgG1 hinge that connects these two paratopes. Extended hinge is shown for comparison. (**c**) Gold-standard Fourier shell correlation (FSC) curve calculated from independently refined half-maps indicate an overall resolution of 7.6 Å. Solid line at 0.143 FSC.

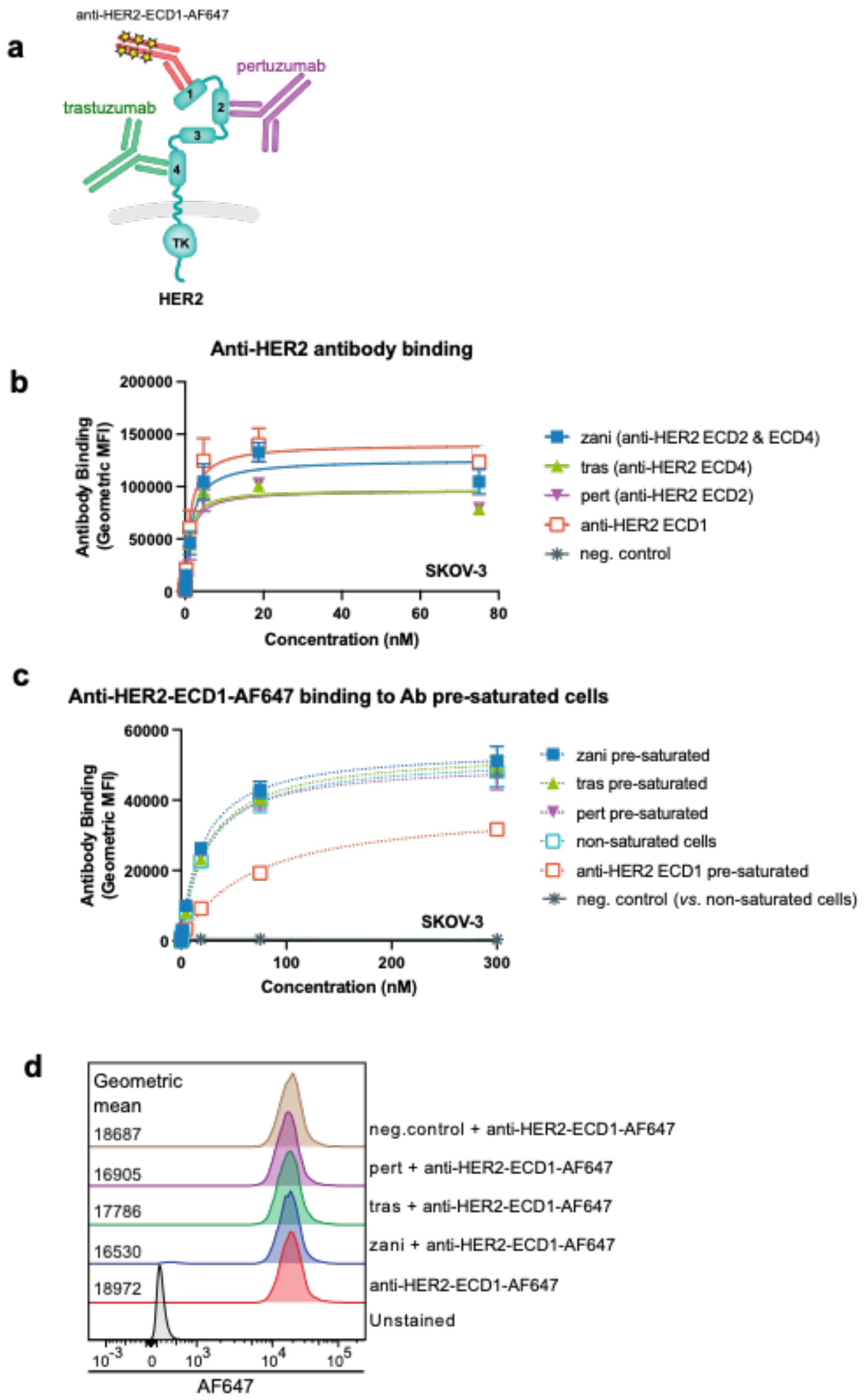

Supplementary Fig. 5. Anti-HER2 ECD1 monovalent antibody (anti-HER2-ECD1-AF647) detects HER2 equivalently in the absence or presence of zanidatamab, trastuzumab or pertuzumab pre-saturated binding. (a) Graphical illusion of antibody binding sites on HER2 ECD including trastuzumab binding to ECD4, pertuzumab binding to ECD2 and anti-

HER2-ECD1-AF647 binding to ECD1. The anti-HER2-ECD1 Ab was conjugated to AF647 by the method described at a fluor to Ab ratio of 8.9 in the confocal and dSTORM analysis of SKBR3 cells, and at a fluor to Ab ratio of 8.1 in the confocal analysis of NCI-N87 cells (Supplementary Table 12). (**b)** Concentration-dependent binding of the anti-HER2 Abs, including anti-HER2-ECD1 Ab, to SKOV-3 cells was evaluated by flow cytometry. (**c)** Anti-HER2-ECD1-AF647 Ab binding to SKOV-3 cells that were pre-incubated with saturating concentration (100 nM) of zanidatamab, trastuzumab or tras + pert was evaluated by flow cytometry. Binding of anti-HER2-ECD1-AF647 and negative control Ab to non-pre-saturated SKOV-3 cells were tested in parallel. (**d)** Binding of the anti-HER2 Abs was confirmed on SK-BR-3 cells by flow cytometry prior to the confocal and dSTORM experiments. Histograms show AF647 fluorescence in SK-BR-3 cells treated with anti-HER2 antibodies at approximately 200 nM, followed by HER2 detection with anti-HER2-ECD1-AF647 (at approximately 40 nM) assessed by flow cytometry. In **b** and **c** data are mean ± SEM from *n*=2 independent experiments. In **d** datapoints are geometric mean values from a single measurement from *n*=2 independent experiments. Gating strategy for **b**, **c** and **d** is shown in Supplementary Fig. 11 b and c. Source data are provided in the Source Data file.

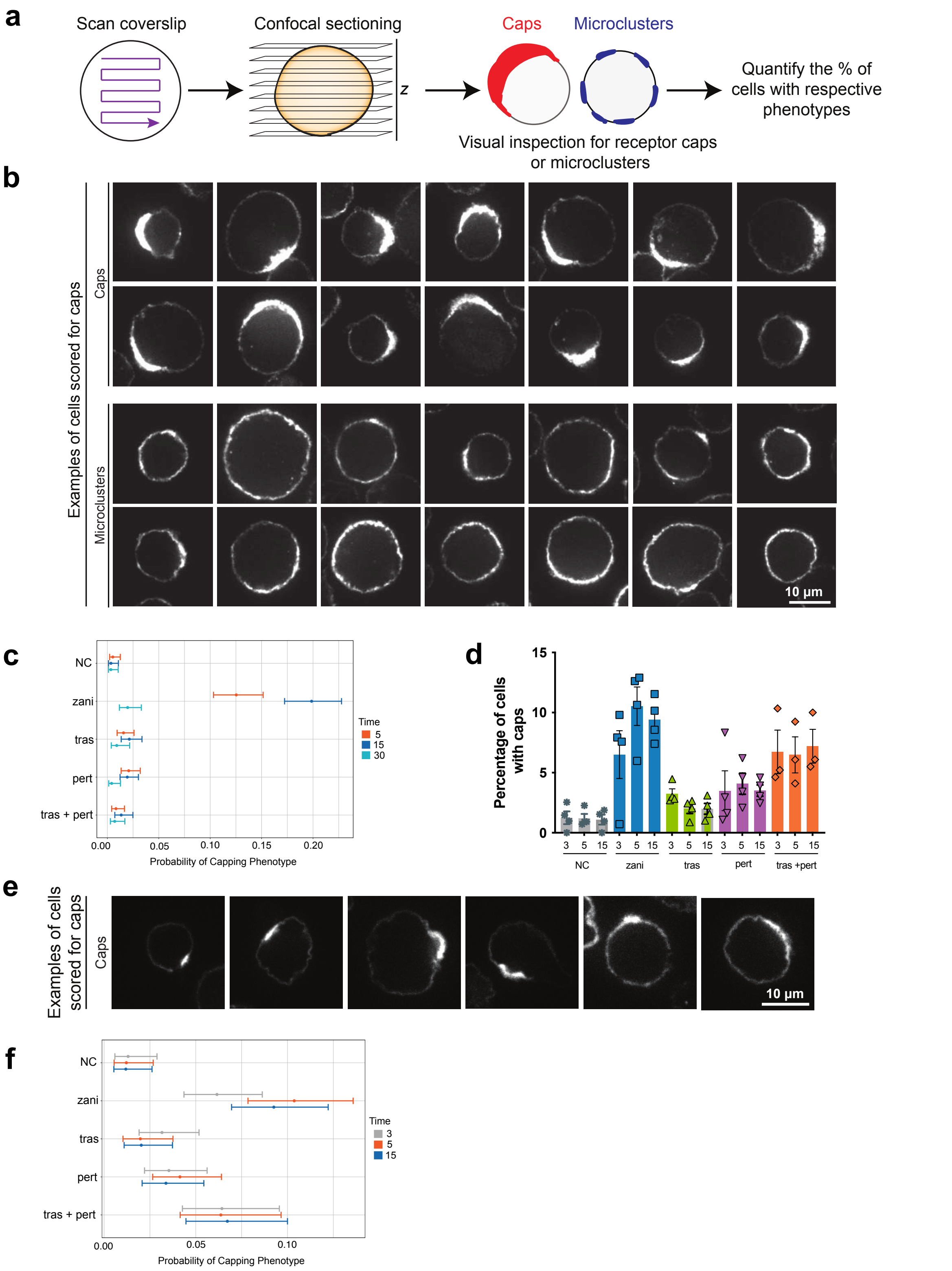

Supplementary Fig. 6. Methodological details and scoring examples of HER2 caps and microclusters analysis by confocal microscopy. (a) Overview of sampling, confocal imaging

and single cell quantification for individual phenotypes. (**b)** Examples of cells scored for HER2 caps (upper) and microclusters (lower**)** in SK-BR-3 cells following treatment with zanidatamab**. (c, f)** Model-predicted probability of capping versus no capping for each treatment and time point in SK-BR-3 (**c**) and NCI-N87 (**f**) cells. A binomial logistic regression model was fit to the discrete phenotypes of "capping" and "no capping". Variation across time points and experimental replicates (*n=3*) for each treatment was modelled by the following equation: phenotype ~ experiment + treatment*time, with p-values representing a two-sided test of parameters from this model. Multiple comparisons were accounted for using the Bonferroni correction. Data are mean ± 95% CI. Pairwise comparisons of model predicted phenotypes with zanidatamab indicate significant differences with all other treatments for the 5 and 15 min time points ($p<0.001$, df = 4) in SK-BR-3 cells (**c**). (**d**) Percentage of NCI-N87 cells with caps, determined by confocal microscopy. NCI-N87 cells were treated with the anti-HER2 and control Abs at 200 nM for 3, 5, and 15 min and imaged by confocal microscopy following detection with the anti-HER2 ECD1-AF647 OAA (fluor to Ab ratio of 8.1) at 74 nM as described. Data are mean ± SEM for three independent experiments (**e**) Examples of cells scored for HER2 caps in NCI-N87 cells following treatment with zanidatamab**. (f)** Model-predicted probability of capping versus no capping for each treatment and time point in NCI-N87 cells using a binomial logistic regression as described. Pairwise comparisons of model predicted phenotypes with zanidatamab or tras + pert indicate significant differences with negative control at 3 min ($p = 0.01$). Pairwise comparisons with zanidatamab indicate significant differences with the negative control or tras at the 5 and 15 min time points ($p < 0.001$, df = 4), or pert at the 5 ($p = 0.01$, df = 4) and 15 min ($p < 0.001$, df = 4) time points. Source data are provided in the Source Data file.

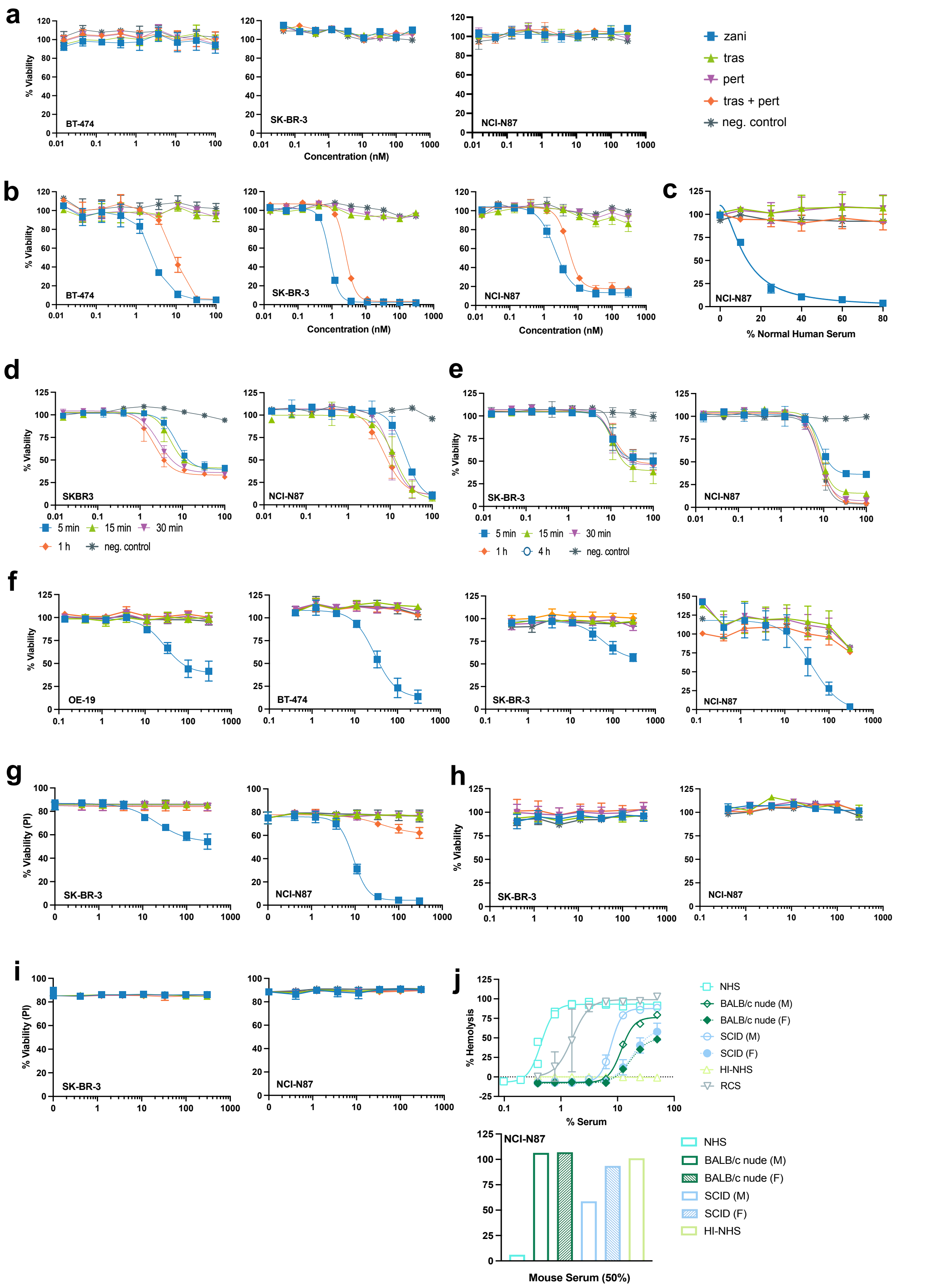

Supplementary Fig. 7. CDC activity in the presence of human, rabbit and mouse complement serum. (a) CDC in the presence of heat inactivated NHS showed that all anti-

HER2 antibodies were inactive. (**b**) CDC in the presence of rabbit complement serum showed activity with zanidatamab and tras + pert, but not trastuzumab or pertuzumab. (**c**) Zanidatamab (10 nM) elicited CDC in the presence of a dose titration of NHS (0-80%) in NCI-N87 cells. (**d**) Pre-incubation of zanidatamab with SK-BR-3 or NCI-N87 cells for 5, 15, 30 min or 1 hr, prior to addition NHS, showed CDC activity at all timepoints. (**e**) Zanidatamab elicited CDC of SK-BR-3 and NCI-N87 cells when incubated in the presence of NHS for 5, 15, 30 min, 1 and 4 hours. (**f**) CDC in the presence of NHS, where NHS was added first (5 min, 37°C), followed by anti-HER2 and control Abs (2.5 h, 37°C) by plate method (**g**) CDC in the presence of NHS, where NHS was added first (15 min, 37°C), followed by anti-HER2 and control Abs (3 h, 37°C), cells were stained with PI and viability assessed by flow cytometry. (**h**) CDC in the presence of NHS + 0.01 M EDTA measured by plate method. (**i**) CDC in the presence of NHS + 0.01 M EDTA measured by flow cytometry. (**j**) Upper: Measurement of mouse serum CDC activity using the CH50 erythrocyte lysis assay showed impaired CDC with male and female Balb/c and SCID mouse serum compared to NHS. RCS, rabbit control serum. Lower: Zanidatamab (100 nM) did not induce CDC of NCI-N87 cells in the presence of 50% female SCID, male Balb/c or female Balb/c serum. Zanidatamab induced 40% CDC of NCI-N87 in the presence of 50% male CB17 SCID mouse serum (right). Data are mean ± SEM from (*n=3*) independent experiments in **a, b** (NCI-N87), **c, d** (SK-BR-3)**, e, f** (BT-474, NCI-N87)**, g** (NCI-N87)**, h** and **i**; from *n=5* or *n=7* independent experiments in **f** (OE19) and **g** (NCI-N87), respectively. In **b** (BT-474) and **j** (upper) data are mean from *n=2* independent experiments; **j** (lower) data represent *n*=1 measurement. Gating strategies for **f** and **i** are shown in Supplementary Fig. 12c and b, respectively. Source data are provided in the Source Data file.

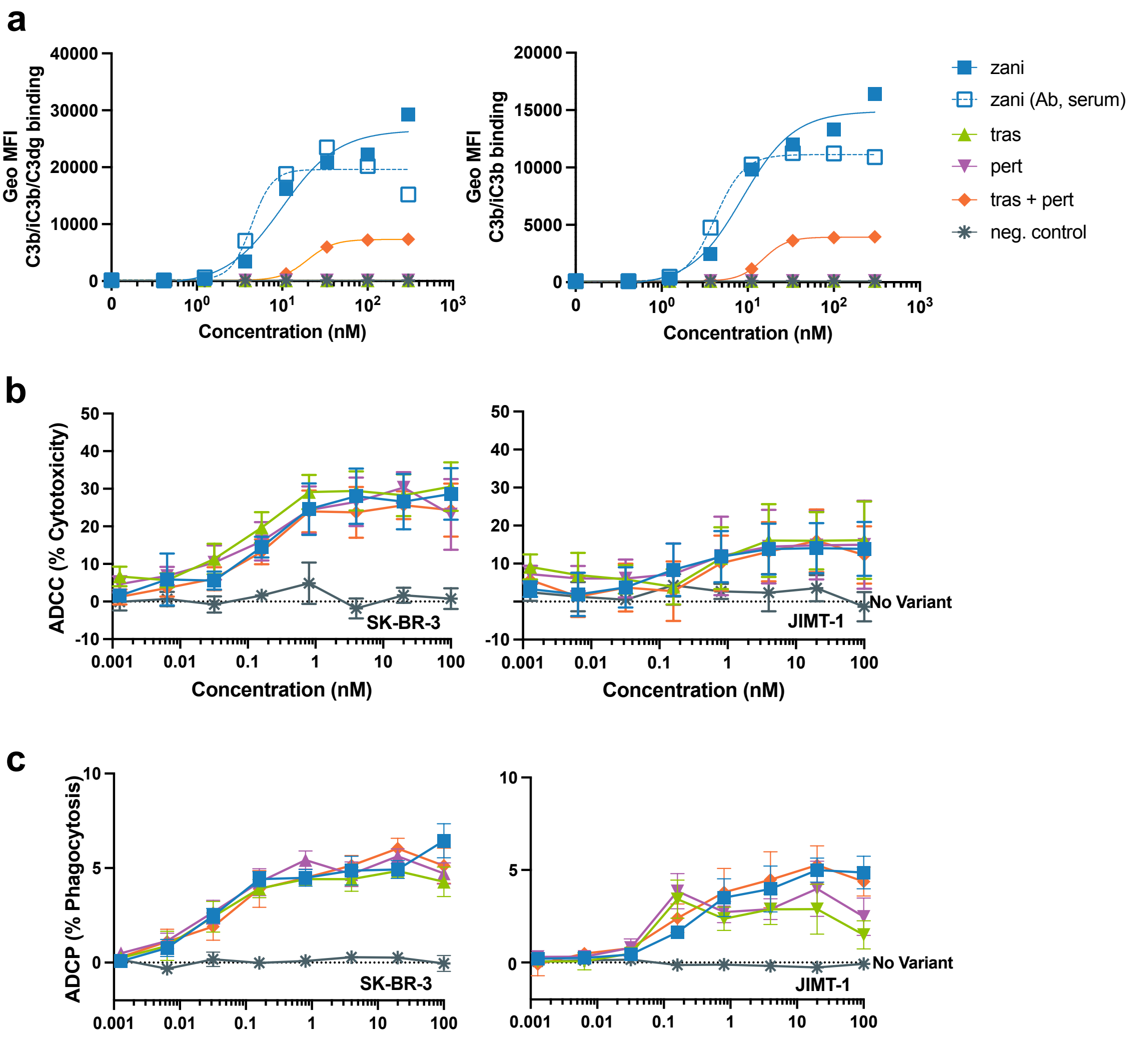

Supplementary Fig. 8. C3 fragment deposition, ADCC and ADCP. (**a**) Zanidatamab mediated the highest C3b/iC3b/C3dg (left) and C3b/iC3b (right) deposition on SK-BR-3 cells in the presence of NHS. Cells were incubated with 25% NHS (15 min, 37°C, 5% CO_2), followed by titrated amounts of anti-HER2 or negative control Abs (15 min 37°C, 5% CO_2). In zani (Ab, serum; open blue square) treatment, zanidatamab was incubated with tumor cells for 15 min and 25% NHS added for 15 min. (**b-c**) Equivalent concentration-dependent ADCC (**b**) and ADCP (**c**) activity between with zanidatamab, trastuzumab, pertuzumab and tras + pert in SK-BR-3 (HER2 3+) and JIMT-1 (HER2 2+). In **a** data are mean from *n=2* technical replicates from *n=1* experiment. Data from a *n=2* experiment tested at a single Ab concentration is provided in the Source Data file. In **b** (SK-BR3) and **c**, data are mean ± SEM from *n=3* independent experiments with biologically independent PBMC samples (**b**), or with macrophage derived from three biologically independent PBMC samples (**c**). In **b** (JIMT-1) data mean ± SEM from *n=2* independent experiments. Gating strategies for **a**, **b** and **c** are shown in Supplementary Fig. 12 d, a, and b, respectively. Source data are provided in the Source Data file.

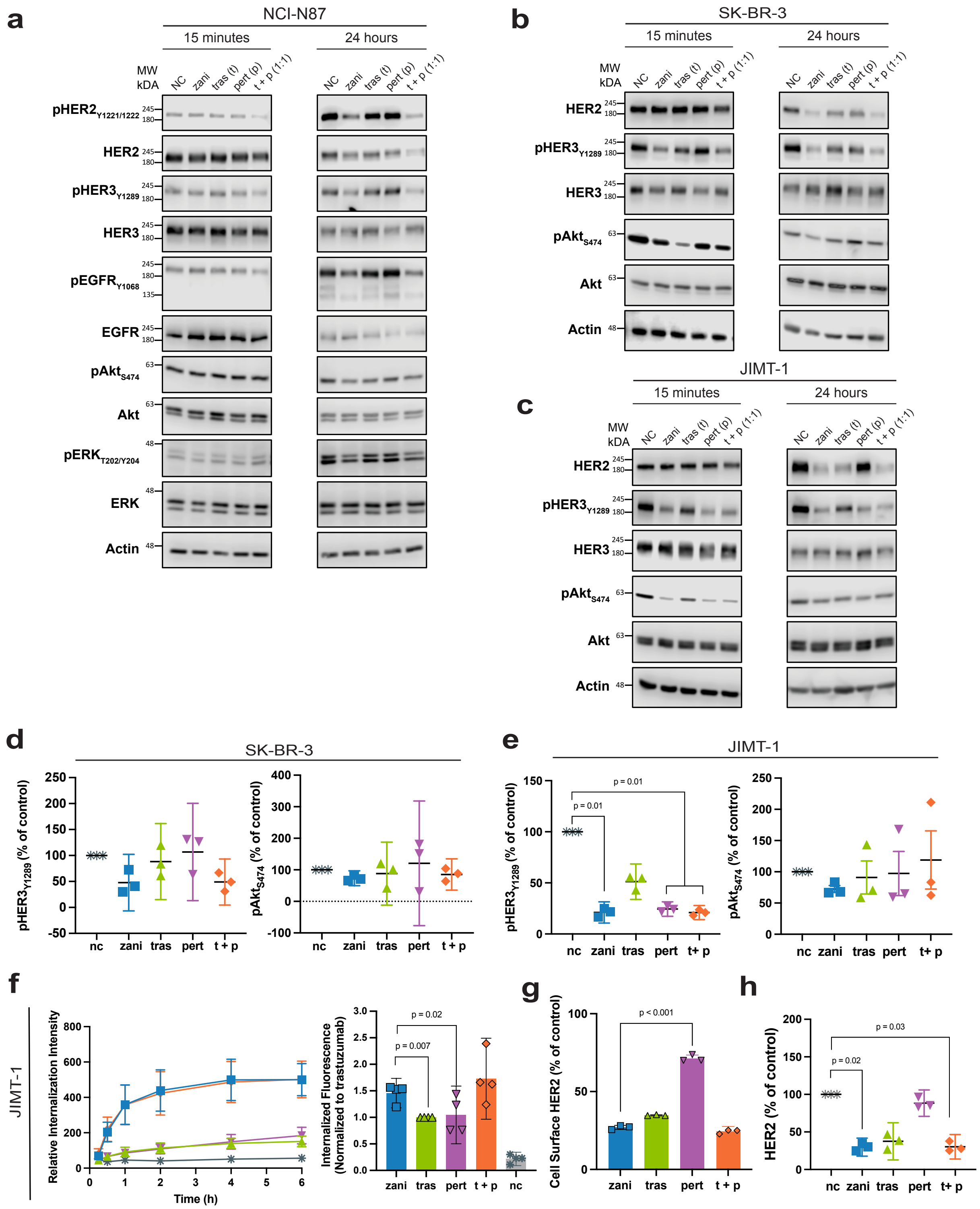

Supplementary Fig. 9. **Intracellular signaling and internalization.** Representative immunoblots of pHER3, HER3, pAKT, AKT and actin control in (**a**) NCI-N87, (**b**) SK-BR-3 and (**c**) JIMT-1 cells after 15 and 24 h after Ab treatment (representative dataset from *n=3* independent experiments). (**d-e**) Zanidatamab mediated inhibition of pHER3 and pAKT in

SK-BR-3 (**d**) and JIMT-1 (**e**) cells when compared to untreated cells following 24 h incubation, evaluated by immunoblotting. (**f**) (Left) Zanidatamab showed increasing receptor mediated internalization compared to trastuzumab or pertuzumab following 15 min to 6 h treatment in JIMT-1 cells, measured by high content microscopy. Data are mean ± SEM from n=3 independent experiments. (Right) Zanidatamab conferred significantly greater receptor mediated internalization compared to trastuzumab (adjusted $p = 0.007$) or pertuzumab (adjusted $p = 0.02$) in JIMT-1 cells following 24 h treatment, measured by flow cytometry. (**g**) Zanidatamab mediated downregulation of surface HER2 in JIMT-1 cells by evaluated by flow cytometry as described. Zanidatamab showed significantly greater HER2 downregulation compared to pertuzumab ($p < 0.001$), ($n=3$ independent experiments, mean ± 95% CI, two-sample two-sided t tests with Bonferroni correction for multiple comparisons, df = 4). (**h**) Total HER2 downregulation of JIMT-1 cells evaluated by immunoblotting showed that zanidatamab reduced total HER2 when compared to untreated cells (adjusted $p = 0.02$) following 24 h treatment. In **d** (df = 2), **e** (df = 2), and **h** (df = 2), ($n=3$ independent experiments, mean ± 95% CI, one sample two-sided t-test compared to untreated cells value of 100% with p-values adjustment using Benjamini & Hochberg false discovery, comparisons with adjusted p-values < 0.05 are shown). In **f** (right) data are mean ± 95% CI from $n=4$ independent experiments, two-way ANOVA to control for experiment with df = 2 with Bonferroni correction for multiple comparisons, data is plotted normalized to trastuzumab treatment). Gating strategy for **f** (right) and **g** is shown in Supplementary Fig. 12 e, f. Uncropped blots from **a-c** are shown in Supplementary Fig. 13 and 14. Source data are provided in the Source Data file.

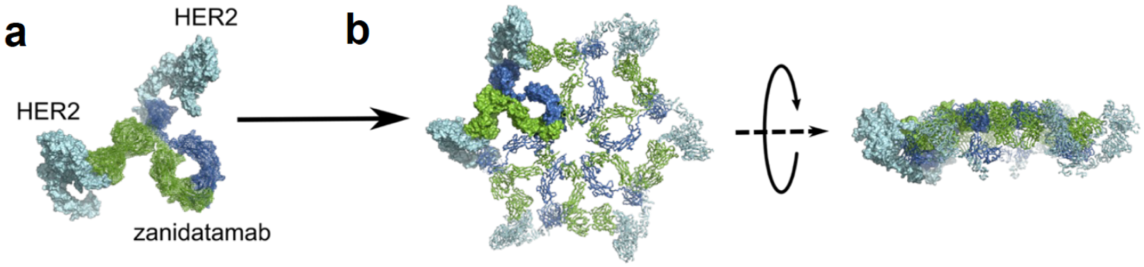

Supplementary Fig. 10. Hexamerization model of zanidatamab:HER2 complex. (**a**) A model of zanidatamab bound to two HER2 molecules (zanidatamab:2xHER2) was built as described in the Supplementary Results. (**b**) A cyclic hexamer was built from the zanidatamab:2xHER2. The IgG hexamer of zanidatamab binds in a bridging configuration to 6 copies of HER2. The orientation of the six bound HER2 molecules align parallel to each other such as may be expected if they were on a cell membrane (right panel).

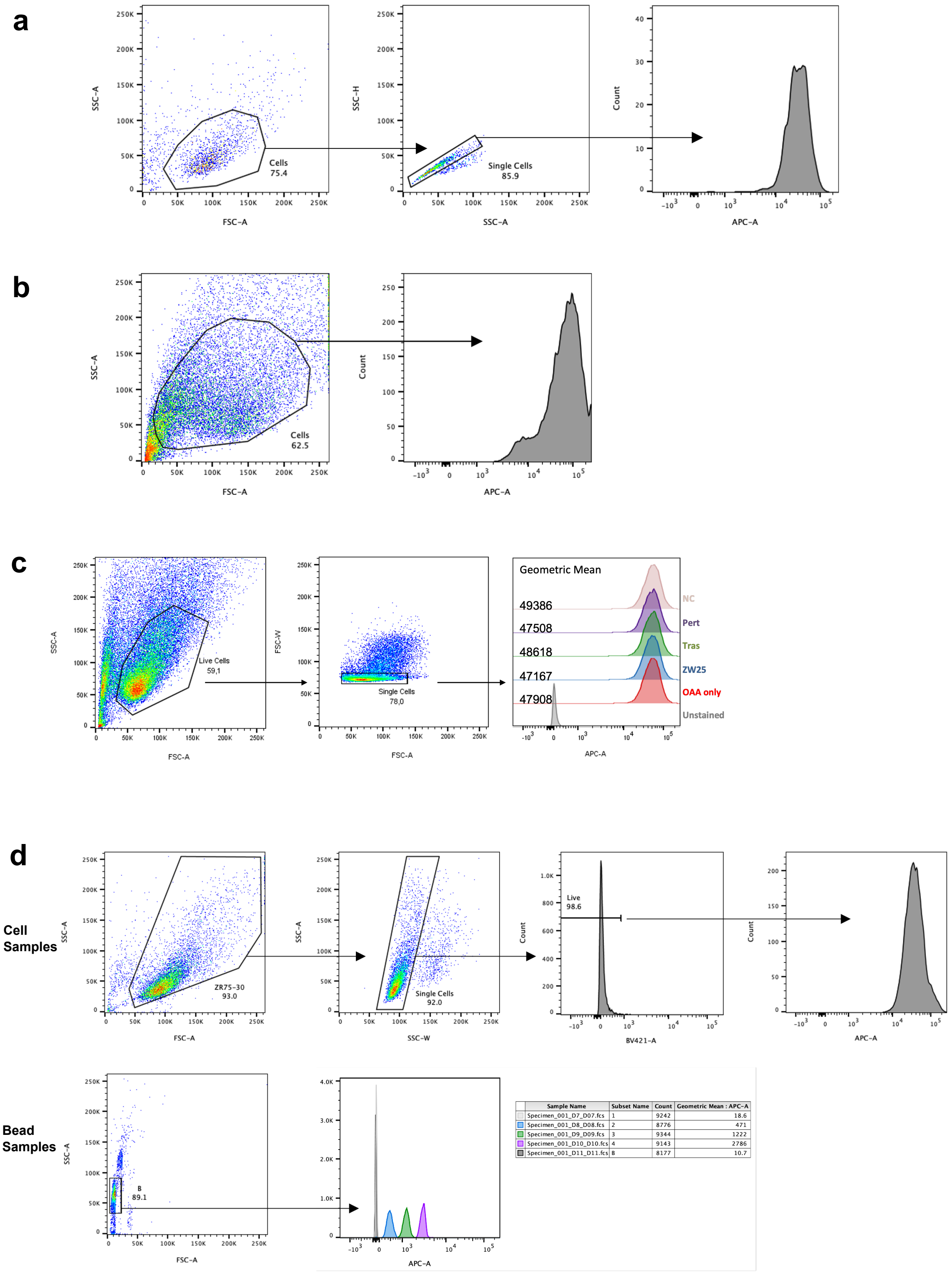

Supplementary Fig. 11. Gating strategies used for on-cell antibody binding and receptor quantification. (**a**) Gating strategy to determine on-cell antibody binding to tumor cells presented

in Fig. 1c and Supplementary Fig. 2. (**b**) Gating strategy to determine on-cell antibody binding to SKOV-3 cells presented in Supplementary Fig. 5 b, c. (**c**) Gating strategy to determine on-cell antibody binding to SK-BR-3 cells presented in Supplementary Fig. 5d. (**d**) Gating strategy to quantify HER2 on tumor cells presented in Table 1.

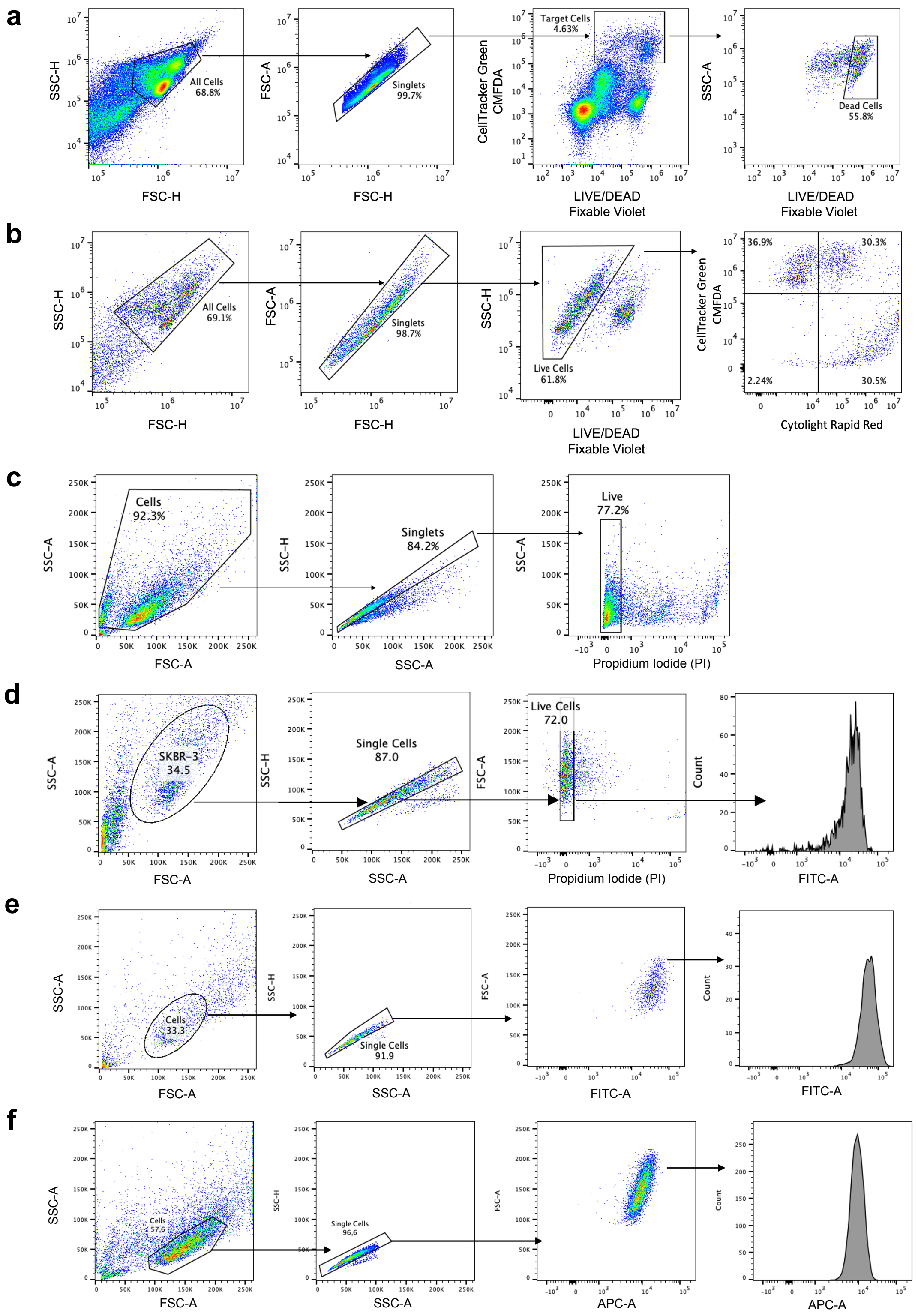

Supplementary Fig. 12. Gating strategies used for ADCC, ADCP, CDC, C1q and C3 fragment binding, internalization and receptor downregulation. (**a**) Gating strategy to determine on-cell ADCC (% cytotoxicity) presented in Fig. 4c and Supplementary Fig. 8b. (**b**)

Gating strategy to determine ADCP (% phagocytosis) presented in Fig. 4d and Supplementary Fig. 8c. (**c**) Gating strategy to determine CDC presented in Supplementary Fig. 7g, i. **(d)** Gating strategy to determine C1q or C3 fragment binding presented in Fig 4b and Supplementary Fig. 8a. (**e**) Gating strategy determine antibody-mediated receptor internalization in tumor cells presented in Fig. 5a (right) and Supplementary Fig. 9f (right). (**f**) Gating strategy to determine receptor downregulation presented in Fig. 5b and Supplementary Fig. 9g.

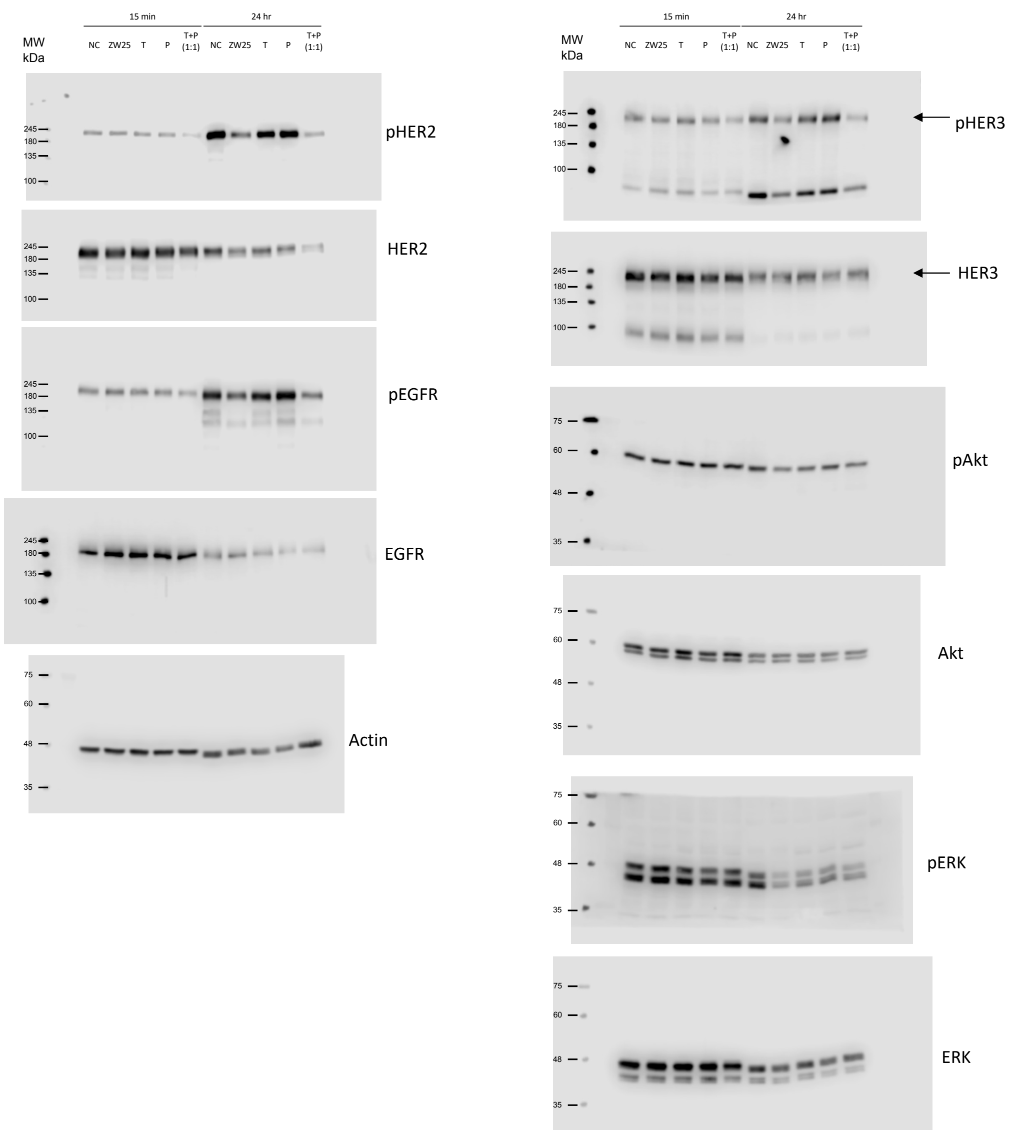

Supplementary Fig. 13. Uncropped blots of NCI-N87 cells in Supplementary Fig. 9a.

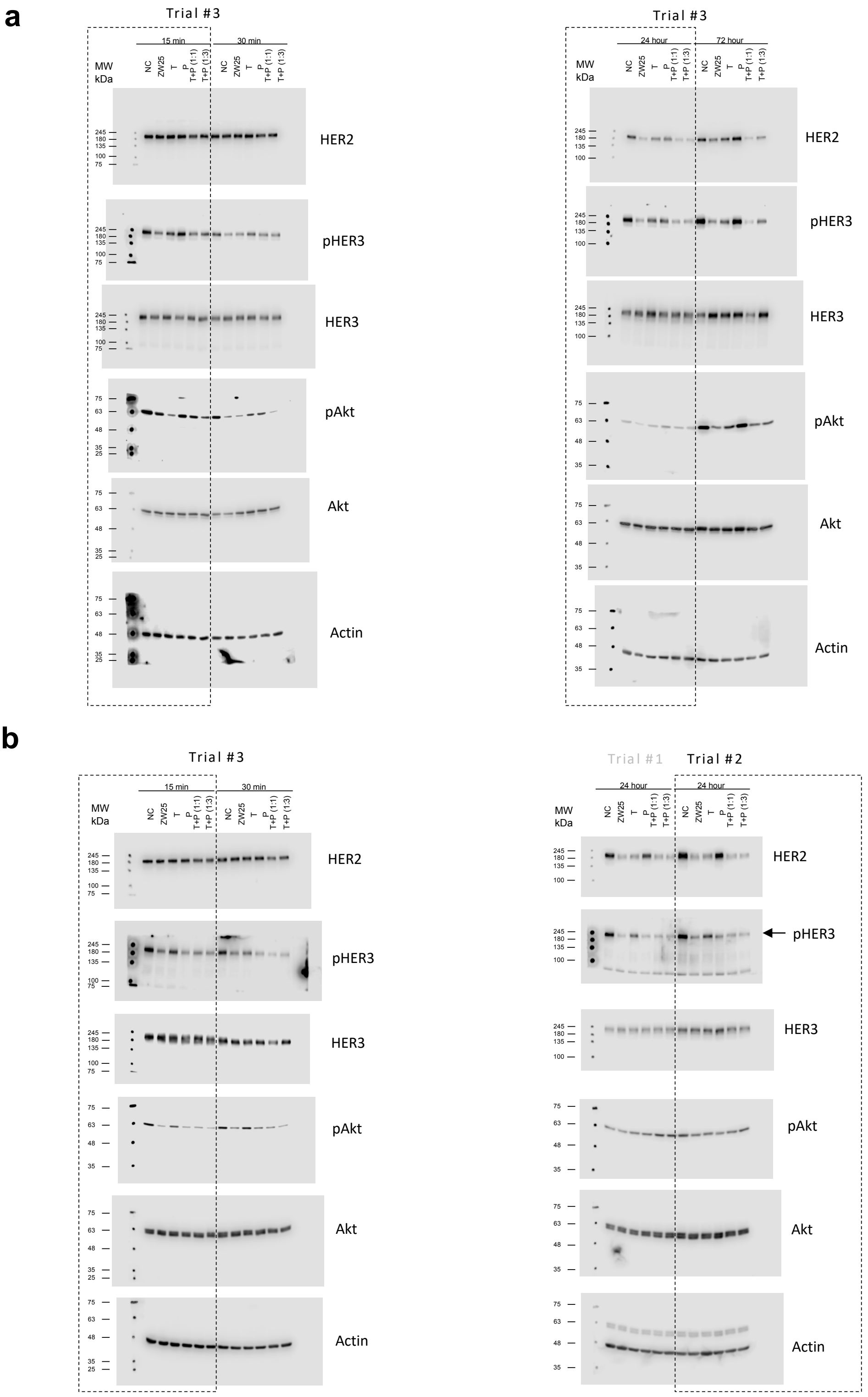

Supplementary Fig. 14. Uncropped blots of SK-BR-3 cells in Supplementary Fig, 9 b (a) and JIMT-1 cells in Supplementary Fig. 9c (b). Dashed line indicated blots presented in Supplementary Fig. 9b,c.

References

1. Diebolder CA, *et al.* Complement is activated by IgG hexamers assembled at the cell surface. *Science* **343**, 1260-1263 (2014).
