# Supplementary material for: An anti-HER2 biparatopic antibody that induces unique HER2 clustering and complement-dependent cytotoxicity
Source: Nat Commun. 2023 Mar 13;14:1394. doi: 10.1038/s41467-023-37029-3 (PMC10011572; doi:10.1038/s41467-023-37029-3)
Supplement: Supplementary file 2 — Description of Additional Supplementary Files [file 41467_2023_37029_MOESM2_ESM.pdf]

Title: Supplementary Data 1.
Description: Summary of the quantification of phospho and total protein levels in SK-BR-3, NCI-N87 and JIMT-1 cells following anti-HER2 Ab treatment

Title: Supplementary Data 2.
Description: P-values for comparison of phospho and total protein level in SKBR-3, NCI-N87 and JIMT-1 levels of anti-HER2 Ab treatment to negative control

Title: Supplementary Movie 1.
Description: Confocal cap/cluster movies
SK-BR-3 cells were stimulated in solution using either negative control or HER2 cross-linking antibodies for 5,15 or 30 minutes. Cells were fixed and surface HER2 was stained using anti-HER2-ECD1-AF647. Cells were imaged using spinning disk confocal microscope and the zstacks were acquired at 0.2 μm step size. 3D reconstruction of z-stacks showing the whole cells and HER2 clusters on the cell surface are shown. Two distinct phenotypes were observed, (a) large HER2 aggregates or 'caps' that were mainly polarized to one side of the cell, and (b) HER2 microclusters that were found on multiple sites on cell surface. Grid size is 500 nm.
