# Supplementary material for: An anti-HER2 biparatopic antibody that induces unique HER2 clustering and complement-dependent cytotoxicity
Source: Nat Commun. 2023 Mar 13;14:1394. doi: 10.1038/s41467-023-37029-3 (PMC10011572; doi:10.1038/s41467-023-37029-3)
Supplement: Supplementary file 3 — Reporting Summary [file 41467_2023_37029_MOESM3_ESM.pdf]

nature portfolio

Corresponding author(s): Nina E. Weisser

Last updated by author(s): 2023/02/27

Reporting Summary

Nature Portfolio wishes to improve the reproducibility of the work that we publish. This form provides structure for consistency and transparency in reporting. For further information on Nature Portfolio policies, see our Editorial Policies and the Editorial Policy Checklist.

Statistics

For all statistical analyses, confirm that the following items are present in the figure legend, table legend, main text, or Methods section.

n/a	Confirmed	
☐	☒	The exact sample size (*n*) for each experimental group/condition, given as a discrete number and unit of measurement
☐	☒	A statement on whether measurements were taken from distinct samples or whether the same sample was measured repeatedly
☐	☒	The statistical test(s) used AND whether they are one- or two-sided<br>*Only common tests should be described solely by name; describe more complex techniques in the Methods section.*
☒	☐	A description of all covariates tested
☐	☒	A description of any assumptions or corrections, such as tests of normality and adjustment for multiple comparisons
☐	☒	A full description of the statistical parameters including central tendency (e.g. means) or other basic estimates (e.g. regression coefficient) AND variation (e.g. standard deviation) or associated estimates of uncertainty (e.g. confidence intervals)
☐	☒	For null hypothesis testing, the test statistic (e.g. *F*, *t*, *r*) with confidence intervals, effect sizes, degrees of freedom and *P* value noted<br>*Give P values as exact values whenever suitable.*
☒	☐	For Bayesian analysis, information on the choice of priors and Markov chain Monte Carlo settings
☒	☐	For hierarchical and complex designs, identification of the appropriate level for tests and full reporting of outcomes
☒	☐	Estimates of effect sizes (e.g. Cohen's *d*, Pearson's *r*), indicating how they were calculated

Our web collection on statistics for biologists contains articles on many of the points above.

Software and code

Policy information about availability of computer code

Data collection	Flow cytometry data was collected using FACSDiva v9.<br>dSTORM was performed using a custom-built microscope with a sample drift-stabilization system that has been described previously (reference 62, Tafteh et al. 2016; reference 63 Tafteh et al. 2016).<br>StormGraph code used for the SMLM analysis (Fig 3e) is described (reference 37, Scurll et al. 2020) and has been deposited on Bitbucket https://bitbucket.org/jscurll/stormgraph_public2022/src/master/.<br>The kinetic model used to simulate crosslinked biparatopic antibody and HER2 species (Fig S1a-c) was developed and simulated in MATLAB version R2019_b, using the Simbiology package and is available upon request .
Data analysis	Flow cytometry analysis used FlowJo 10 (BD LSRFortessa X-20) or Forecyt (Sartorius Intellicyte iQue3).<br>The StormGraph code used to determine localization coordinates and quantify clustering from SMLM images (Fig 3e) is described (reference 37, Scurll et al. 2020) and is available upon request from Dr. J. Scurll (jscurll.ubc@gmail.com).<br>Results the dSTORM (Fig. 3h) and the kinetic model (Fig. S1a-c) were plotted using the ggplot2 visualization package in R.<br>Statistical analysis was performed in R v4.2.1, code is available upon request.<br>Prism software (GraphPad, v9.2.0 or v9.4.1) was used for other data analyses (Fig.1c, 2a, 3f-g, 4, 5, 6, 7).

For manuscripts utilizing custom algorithms or software that are central to the research but not yet described in published literature, software must be made available to editors and reviewers. We strongly encourage code deposition in a community repository (e.g. GitHub). See the Nature Portfolio guidelines for submitting code & software for further information.

Data

Policy information about availability of data

All manuscripts must include a data availability statement. This statement should provide the following information, where applicable:

- Accession codes, unique identifiers, or web links for publicly available datasets
- A description of any restrictions on data availability
- For clinical datasets or third party data, please ensure that the statement adheres to our policy

The atomic models generated in this study have been deposited into the Protein Data Bank with accession number PDB 8FFJ. The corresponding cryo-EM density maps generated in this study have been deposited into the Electron Microscopy Data Bank with accession numbers EMD-29044. Other atomic models used in this study are available with accession numbers 1N8Z, 1S78, 6OGE. The remaining data are available within the Article, Supplementary Information, Source Data file or available from the corresponding author upon reasonable request. Source data are provided with this paper.

Human research participants

Policy information about studies involving human research participants and Sex and Gender in Research.

Reporting on sex and gender	Not applicable.
Population characteristics	Not applicable.
Recruitment	Not applicable.
Ethics oversight	Not applicable.

Note that full information on the approval of the study protocol must also be provided in the manuscript.

Field-specific reporting

Please select the one below that is the best fit for your research. If you are not sure, read the appropriate sections before making your selection.

☒ Life sciences ☐ Behavioural & social sciences ☐ Ecological, evolutionary & environmental sciences

For a reference copy of the document with all sections, see nature.com/documents/nr-reporting-summary-flat.pdf

Life sciences study design

All studies must disclose on these points even when the disclosure is negative.

Sample size	A calculation of sample size by power analysis was used to determine animal numbers for in vivo studies that would allow for robust statistical analysis. Here, sample size estimation was performed using a pilot dataset from which variance parameters and effect size are approximated by fitting a mixed effects model. Using this model, data was simulated by sampling the fitted distributions multiple times across a range of different sample sizes and effect sizes. For each simulated dataset, the hypothesis test procedure was performed to produce a p-value, and the power is calculated as the proportion of simulations for which the p-value is below 0.05. A sample size was chosen that satisfied a desired effect size for a given study at a power of 90%. For all other studies, no sample-size calculations were performed. Sample size was determined to be adequate based on the magnitude and consistency of measurable differences between groups.
Data exclusions	Data exclusions for the SPR data (Fig 2a and Fig S3b) are described in the methods and follow standard procedures for acceptance of model fitting for kinetic values. Kinetic values that had chi-square values > 10% of the maximum binding capacity (Rmax), U-values > 25 or tc values (i.e. the flow rate-independent component values, a modification of the mass transfer constant) in the range of 10E7 – 10E9 with low SE (indicating mass transport) were excluded from the linear regression analysis. The experiment in Fig 2a is focused on measuring discrete changes in kd (off-rate) over low to high captured antibody surface densities on the chip, thus stringent acceptance criteria were applied to fitted kd (off-rate), ka (on-rate) values and subsequent Kd values. One NCI-N87 CDC experiment was not included in the n=6 data plot in figure 4a because this experiment tested a different concentration range compared to the other n=6 experiments; however, the fitted max effect data are included in the n=7 meta analysis in Table 1.
Replication	For the majority of experiments a minimum of 3 experimental replicates were performed. For supplementary experiments, a minimum of 2 and typically 3 experiments were performed. All attempts are replication were successful, with one exception. Two of three PBMC donor showed ADCC activity in JIMT-1 cells, one donor was inactive with all anti-HER2 Abs tested in JIMT-1 cells.
Randomization	Animals in the in vivo study were randomly assigned to the blinded treatment groups. For all other studies, samples were allocated to experimental group without any previous selection.
Blinding	For in vivo studies, samples were blinded to the experimentalists performing the work, measurements and initial data analysis. For the confocal experiments in Fig 3c,d and Fig Sb,e, the experimenter was not blinded and all data was independently reviewed and score verified by 1-2 additional reviewers non-blinded.

For all other studies, blinding was not relevant because no bias could be made by the subject or the tester in the experiments performed.

Reporting for specific materials, systems and methods

We require information from authors about some types of materials, experimental systems and methods used in many studies. Here, indicate whether each material, system or method listed is relevant to your study. If you are not sure if a list item applies to your research, read the appropriate section before selecting a response.

Materials & experimental systems

n/a	Involved in the study	
☐	☒	Antibodies
☐	☒	Eukaryotic cell lines
☒	☐	Palaeontology and archaeology
☐	☒	Animals and other organisms
☒	☐	Clinical data
☒	☐	Dual use research of concern

Methods

n/a	Involved in the study	
☒	☐	ChIP-seq
☐	☒	Flow cytometry
☒	☐	MRI-based neuroimaging

Antibodies

Antibodies used	All antibodies used, including catalogue number and supplier details, are detailed in Supplementary Table 11. AF647 conjugated-goat anti human IgG-Fc secondary antibody (cat no. 109-605-098, Jackson Labs); FITC-conjugated anti-C1q (cat no. Ab4223, Abcam); FITC-conjugated anti-C3/C3b/iC3b/C3dg (cat no. CL7637F, Cedarlane); Goat anti-human Fc polyclonal Ab (cat no. 109-005-098, Jackson ImmunoResearch Labs); anti-pHER3 (cat no. 2842, Cell Signaling Technology); anti-HER3 (cat no. 12708, Cell Signaling Technology); anti-pEGFR (cat no. 2234, Cell Signaling Technology); anti-EGFR (cat no. 2232, Cell Signaling Technology); anti-pHER2 (cat no. 2243, Cell Signaling Technology); anti-HER2 (cat no.2165, Cell Signaling Technology); anti-pAKT (cat no. 8599, Cell Signaling Technology); anti-AKT (cat no. 4691, Cell Signaling Technology); anti-pERK (cat no. 4370, Cell Signaling Technology); anti-ERK (cat no.4695, Cell Signaling Technology); anti-beta actin (cat no. PA5-16914, Invitrogen); HRP-conjugated goat anti-rabbit IgG (cat no. 170–6515, BioRad); Goat anti-human IgG-Fc Fab fragment AF488 conjugate (cat no. 109-547-008, Jackson ImmunoResearch); Rabbit IgG anti-488 Ab (cat no. A-11094; Life Technologies).
Validation	A list with all antibodies used and antibody source and dilution is provided in Supplementary Table 11. Validation to human protein target was performed following variable strategies: cellular localization, molecular weight of bands in western blotting experiments; binding to recombinant target protein by ELISA, and control tissues, either by vendor and/or herein.

Eukaryotic cell lines

Policy information about cell lines and Sex and Gender in Research

Cell line source(s)	Cell lines were obtained from ATCC (HCC2218, HCC1954, HCC1419, ZR-75-30, AU565, NCI- 2170, BT-474, SK-BR-3, NCI-N87, MCF7), Sigma (OE-19), DSMZ (JIMT-1), Cedarlane (ZR-75-1) and AddexBio (MDA-MB-175-VII, MDA-MB-468).
Authentication	Cell lines were authenticated by supplier and cell lines were used from source and expanded for a maximum of 20 passages.
Mycoplasma contamination	Cell lines were routinely spot check tested for mycoplamsa, all tests were negative.
Commonly misidentified lines (See ICLAC register)	MCF7 and ZR-75-1 were used as representative HER2-low cell lines. MCF7 was used for on cell binding and complement dependent cytotoxicity (CDC) assays and ZR-75-1 cell line was used in the CDC assay. Data and conclusions from the MCF7 and ZR-75-1 cell lines are supported with data from other HER2-low cell lines including JIMT-1 and MDA-MB-175-VII.

Animals and other research organisms

Policy information about studies involving animals; ARRIVE guidelines recommended for reporting animal research, and Sex and Gender in Research

Laboratory animals	Five to seven week old female athymic nude mice (NMRI-Foxn1nu, Harlan Laboratories) were used in the GXA 3054 PDX in vivo study. Six to eight week female BALB/c nude mice were used in the NCI-N87 xenograft in vivo study. Mouse strain serum was collected from six to eight week old male and female Balb/c, and male and female CB17 SCID.
Wild animals	The study did not involve wild animals.
Reporting on sex	Not applicable.
Field-collected samples	The study did not involve samples collected from the field.
Ethics oversight	All animal studies were carried out in accordance with all applicable international, national, and local laws and guidelines and protocols approved by the regional council Committee of Ethics of Animal Experts (Oncotest GmbH) and Institutional Animal Care and

Use Committee (CrownBio).
Mouse strain serum (male and female Balb/c, male and female CB17 SCID) was collected under protocols approved by the Animal Care Committee at University of British Columbia.

Note that full information on the approval of the study protocol must also be provided in the manuscript.

Flow Cytometry

Plots

Confirm that:

- [x] The axis labels state the marker and fluorochrome used (e.g. CD4-FITC).
- [x] The axis scales are clearly visible. Include numbers along axes only for bottom left plot of group (a 'group' is an analysis of identical markers).
- [x] All plots are contour plots with outliers or pseudocolor plots.
- [x] A numerical value for number of cells or percentage (with statistics) is provided.

Methodology

Sample preparation

On-cell binding assays: The target tumor cells at 40,000 cells/well were incubated in FACS buffer (PBS + 2% FBS) with primary antibodies for 1 h at 4°C. Cells were washed twice with FACS buffer and incubated with a secondary anti-human IgG antibody for 1 hr at 4°C. Following incubation, cells were washed with FACS buffer.

C1q and C3 fragment binding assays: C1q and C3 binding: The target tumor cells at 50,000/well were washed twice in serum free media and incubated in FACS buffer in the presence of pooled normal human serum and anti-HER2 antibodies for 15 min at 37°C. The target tumor cells were then incubated in FACS buffer (PBS + 2% FBS) with primary antibodies for 45 min at 4°C. Cells were then washed twice with FACS buffer.

CDC assays: The target tumor cells were incubated in FACS buffer in the presence of pooled normal human serum and anti-HER2 antibodies for 3 h at 37°C. Cells were stained with Propidium Iodide.

ADCC Assays: Human PBMCs were rested overnight in RPMI + 10% ultra low IgG FBS + 100 U/mL recombinant human IL-2 at 37°C. The following day, target tumor cells were stained with CMFDA and incubated with the rested PBMCs at an effector to target ratio of 5:1 and anti-HER2 antibodies for 4 h at 37°C. Cells were washed twice in FACS buffer and stained for viability with LIVE/DEAD Fixable Violet Dead Cell Stain.

ADCP Assays: Human monocytes were differentiated into macrophages by culturing human PBMCs in RPMI + 10% FBS + 10 ng/mL MCSF for 8 days. Macrophages were lifted and stained with Cytolight Rapid Red and co-cultured with tumor target cells stained with CellTracker Green CMFDA at an effector to target ratio of 2:1 for 1.5 h at 37°C. Cells were stained for viability using LIVE/DEAD Fixable Violet Dead Cell Stain.

Internalization Assays: Primary antibodies were coupled to anti-human IgG Fab fragment, covalently conjugated to AF488 fluorophore, at a 1:1 molar ratio for 24 h at 4°C. Target tumor cells were incubated in culture media with primary antibodies coupled to Fab-AF488 for 24 h at 37°C and 15 min at 4°C. Following incubation, cells were dissociated and washed with FACS buffer (PBS + 2% FBS). For a subset of samples, surface AF488 fluorescence was quenched with an anti-AF488 antibody for 45 min at 4°C.

HER2 Surface Downregulation Assays: Target tumor cells were seeded in 48-well plates at 50,000 cells/well. Next day, cells were treated with antibody for 24 hrs at 37°C. Following incubation, cells were harvested and stained with a non-competing, fluorescently labeled anti-HER2 antibody AF647 conjugate for 45 min at 4°C. Following incubation, cells were dissociated and washed with FACS buffer (PBS + 2% FBS).

Receptor Quantification Assays: Target tumor cells were seeded in 96-well plates at 50,000 cells/well. One drop of quantification bead sets 1-5 were added to separate eppendorf tubes. Target tumor cells were prepared with a violet viability stain and stained with or without a saturating level of fluorescently labeled anti-HER2 antibody AF647 conjugate and violet viability stain. Similarly, quantification beads sets 1-4 were incubated with anti-HER2 antibody AF647 conjugate and violet viability stain. Quantification bead set 5 (blank) was prepared in FACS buffer (PBS + 2% FBS) only. All samples were incubated for 30 min on ice and subsequently washed with FACS buffer.

Instrument

BD LSRFortessa X-20, Sartorius Intellicyte iQue3

Software

BD FACSDiva v9, Forecyt, FlowJo v10

Cell population abundance

All flow cytometry analysis was done on pure cultures of individual cell lines, not mixed cell populations. No statements are made about population abundance.

On cell Ab, C1q and C3 fragment binding: In all the experiments, a control sample lacking primary antibody was included and at least 2,000 events were acquired.

CDC: In all experiments, a control sample lacking antibody was used and at least 10,000 events in the singlet cell gate were

acquired.

ADCC: All flow cytometry analysis was done on pure cultures of individual cell lines in co-culture with human PBMCs. In all experiments, a control sample lacking antibody was used and at least 1500 events in the dead cell gate were acquired.

ADCP: All flow cytometry analysis was done on pure cultures of individual cell lines (stained with CellTracker Green CMFDA) in co-culture with mature human macrophages (stained with Cytolight Rapid Red). In all experiments, a control sample lacking antibody was used and at least 2000 events in the CMFDA+ Cytolight Red+ gate were acquired in duplicate. Human macrophage purity after 8 days of maturation in 10 ng/mL of M-CSF was determined by CD14 and CD11b expression by flow cytometry. Macrophage purity was determined to be 80% ± 11% (mean ± SD) for all experiments.

Internalization and receptor depletion assays: All flow cytometry analysis was done on pure cultures of individual cancer cell lines. In all experiments, a control sample lacking primary antibody was included; however, only the MFI for the stained cell population was used for calculating fold-over internalization values and % cell surface HER2. No comparison of fluorescent values with stained/unstained samples were done. At least 1,000 events (SK-BR-3, JIMT-1) and 500 events (NCI-N87) were acquired.

Receptor quantification assays: MFI values of stained and unstained cell populations along with MFI from quantification beads were input into Bang Laboratories' calibration template to obtain calculated ABC (antigen binding capacity). To obtain receptor density or final ABC values, ABC (unstained) was subtracted from ABC (stained) for each individual cancer cell line. In all experiments, a control sample lacking primary antibody was included and at least 1,500 events were acquired.

Gating strategy

On-cell binding and CDC assays: All samples were first gated using forward scatter and side scatter to identify events corresponding to cells, and then using side scatter height vs. area to enrich for single cells. Events were further gated on live cells (negative for viability dye) for all experiments except Fig S5d).

C1q and C3 fragment binding assays: Cells were gated based on size and granularity using SSC-A vs FSC-A to eliminate debris. Single cells were sub-gated using SSC-H vs SSC-A. Live cells were determined by negativity for Propidium Iodide (PI). FITC fluorescence geometric mean was determined in the singlet, live cell population.

ADCC assays: All samples were first gated using forward scatter and side scatter to identify events corresponding to cells, and then using side scatter height vs area to enrich for single cells. Cytotoxicity of cells was determined by gating on viability dye and CFSE positive events.

ADCP assays: All samples were first gated using forward scatter and side scatter to identify events corresponding to cells, and then using side scatter height vs area to enrich for single cells. Events were further gated on live cells (negative for viability dye). Phagocytic activity was determined by gating on Cell Tracker Red positive and CellTracker Green positive events. (a) Cells were gated based on size and granularity using SSC-H vs FSC-H to eliminate debris. (b) Single cells were sub-gated using FSC-A vs FSC-H. (c) Target cells were determined by positivity for CMFDA. (d) Dead cells were determined by positivity for Fixable Violet (Live/Dead Stain). Values inside the plots represent the percentages from the parent gate. Values above the plots represent the event count of the parent gate.

Internalization Assays: All samples were first gated using forward scatter and side scatter to identify events corresponding to cells, and then using side scatter height vs. side scatter area to enrich for single cells. AF488 fluorescence geometric mean was determined in the single cell population.

HER2 Surface Downregulation Assays: All samples were first gated using forward scatter and side scatter to identify events corresponding to cells, and then using side scatter height vs. side scatter area to enrich for single cells. AF647 fluorescence geometric mean was determined in the single cell population.

Receptor quantification assays: All cell samples were first gated using forward scatter and side scatter to identify events corresponding to cells, and then using side scatter height vs. side scatter area to enrich for single cells. Gating was performed on live cells by selecting for cells negative for violet viability stain. AF647 fluorescence geometric mean was determined in the single cell population. Bead samples were similarly gated using forward scatter and side scatter to identify events corresponding to beads, followed by AF647 fluorescence geometric mean determination for each bead set.

☒ Tick this box to confirm that a figure exemplifying the gating strategy is provided in the Supplementary Information.
